# Screening for cold tolerance genes in *C. elegans*, whose expressions are affected by anticancer drugs camptothecin and leptomycin B

**DOI:** 10.1038/s41598-024-55794-z

**Published:** 2024-03-05

**Authors:** Misaki Okahata, Natsumi Sawada, Kenji Nakao, Akane Ohta, Atsushi Kuhara

**Affiliations:** 1 Graduate School of Frontier Biosciences, Osaka University Suita, Osaka, Japan; 2 Graduate School of Natural Science, Konan University, Kobe, Hyogo Japan; 3 Faculty of Science and Engineering, Konan University, Kobe, Hyogo Japan; 4 Institute for Integrative Neurobiology, Konan University, Kobe, Hyogo Japan; 5 Biomolecular Research Laboratories, Pharmaceutical Research Division, Takeda Pharmaceutical Company Limited, Fujisawa, Japan; 6 PRIME, AMED, Japan Agency for Medical Research and Development, Tokyo, Japan

**Keywords:** *Caenorhabditis elegans*, Cold tolerance, Camptothecin, Leptomycin B, Genetics, Genetic interaction, Epistasis

## Abstract

Temperature is a vital environmental factor affecting organisms’ survival as they determine the mechanisms to tolerate rapid temperature changes. We demonstrate an experimental system for screening chemicals that affect cold tolerance in *Caenorhabditis elegans*. The anticancer drugs leptomycin B and camptothecin were among the 4000 chemicals that were screened as those affecting cold tolerance. Genes whose expression was affected by leptomycin B or camptothecin under cold stimuli were investigated by transcriptome analysis. Abnormal cold tolerance was detected in several mutants possessing genes that were rendered defective and whose expression altered after exposure to either leptomycin B or camptothecin. The genetic epistasis analysis revealed that leptomycin B or camptothecin may increase cold tolerance by affecting a pathway upstream of the insulin receptor DAF-2 that regulates cold tolerance in the intestine. Our experimental system combining drug and cold tolerance could be used for a comprehensive screening of genes that control cold tolerance at a low cost and in a short time period.

## Introduction

Temperature is an important environmental factor influencing biological responses. In general, environmental temperature affects the lifespan of animals^1–3^. Previous studies have revealed the involvement of environmental information such as temperature and food cues in the lifespan of *Caenorhabditis elegans*. The expression of an insulin-like peptide, *ins-6* is regulated by food cues, thereby affecting the lifespan^4^. *C. elegans* has a shorter lifespan when cultivated at higher temperatures as compared to low temperatures. However, *C. elegans* exhibits an extended lifespan following cultivation at high temperatures, when *cbs-1*, the orthologue of human cystathionine β-synthase, is specifically overexpressed in the intestine^5^. At lower temperatures, lifespan extension is controlled by the cold-sensitive Transient Receptor Potential (TRP) channel, TRPA-1 and adiponectin receptor PAQR-2 that functions as a regulator, linking low temperatures and autophagy^6,7^, *C. elegans* homologue of p23 co-chaperone/prostaglandin E synthase-3, *daf-41,* interacts with insulin signaling, heat shock factors, and steroid signaling pathways, and is responsible for controlling lifespan at both high and low temperatures^8^. In low-temperature conditions, animals maintain homeostasis by altering lipid composition, including the ratio of unsaturated fatty acids in their body^9–12^. Our previous studies suggested that cold tolerance and temperature acclimation in *C. elegans* can be further investigated to elucidate neural circuits and tissue networks^11,13–22^. The mechanisms involved in controlling cold tolerance and temperature acclimation have not yet been fully elucidated.

To identify novel mechanisms regulating cold tolerance or temperature acclimation, in this study, we conducted a large-scale screening of chemicals which affect cold tolerance or temperature acclimation. Results of the drug screening indicated that the anticancer drugs leptomycin B (LMB) and camptothecin (CPT) affect cold tolerance and temperature acclimation in *C. elegans*. During screening of antifungal antibiotics, Leptomycin A and B produced by *Streptomyces* sp. have been observed to induce cell elongation of the fission yeast *Schizosaccharomyces pombe*^23^. Genetic analysis using a fission yeast mutant has led to the isolation of CRM1 (chromosome region maintenance), which is resistant to leptomycin B, and exhibits abnormal deformation of nuclear chromosome domains under restrictive temperatures^24,25^. LMB inhibits nuclear export of proteins such as Rev proteins, with a leucine-rich nuclear export signal (NES), by functioning as an inhibitor of CRM1/exportin1 directly binding to the NES^26–29^. Genome-wide RNAi screening in *C. elegans* has demonstrated that silencing *xpo-1*, cording exportin1 in humans, causes enhanced autophagy by increasing the activity of the nuclear HLH-30/TFEB transcription factor, which is involved in the transcriptional modulation of autophagy^30^.

The anticancer drug camptothecin (CPT) was isolated from the tree *Camptotheca acuminata* in China. An analysis of cultured cells indicated that CPT acts as an inhibitor of topoisomerase I and inhibits DNA synthesis^31^. Topoisomerase I forms a complex with DNA by a covalent bond during DNA replication. CPT interacts with the topoisomerase I–DNA complex, thereby inhibiting DNA replication^31,32^. The budding yeast and fission yeast are sensitive to CPT, however, CPT exerted no influence on cording topoisomerase I in the yeast mutant with *top1* deletion^33–35^. Genetic analysis of yeast using CPT has enabled the isolation of various genes involved in DNA replication and repair. *Saccharomyces cerevisiae* with a mutation in RAD52 was hypersensitive to CPT^34,36^. The budding yeast mutant lacking serine 129 in histone 2A (H2A ser129) appeared to be a hypersensitive phenotype to CPT. H2A ser129 efficiently repairs DNA double-stranded breaks and is a crucial component^37^. Yeasts with mutations in Tof1 and Csm3, which function as checkpoint-mediator proteins during DNA replication, were also found to be hypersensitive to CPT^38^. The abnormality of yeast *tof1Δ* which results in its hypersensitivity to CPT, is suppressed by Sir1-4 mutation. CPT resistance is promoted by of the inhibited deacetylation of histone H4-K16 caused by the inactivation of SIR protein^39^.

In this study, the effect of LMB and CPT on the cold tolerance and temperature acclimation in *C. elegans* was identified following the screening of ~ 4000 drugs. Genes with altered expression following CPT or LMB exposure were identified through RNA sequencing analysis. Several novel genes which influenced cold tolerance, were identified by conducting cold tolerance test of mutants defective in genes, whose expression is altered by CPT or LMB exposure. This experimental system involving chemicals could be used as a model for screening genes regulating cold tolerance.

## Results

### Drug screening using the experimental system of cold tolerance

**Figure 1:**
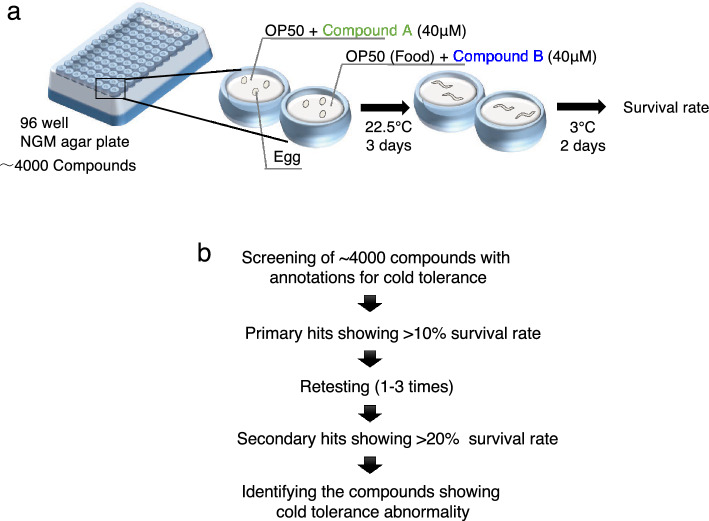
Screening for chemicals that affect cold tolerance (**a**) After cultivation at 22.5 °C from egg to adult stage in NGM containing approximately 4000 different medicinal substances added to 96-well plates, the wild-type animals were exposed to cold stimuli at 3 °C. (**b**) Cold tolerance tests were conducted on animals cultivated in NGM containing ~ 4000 different medicinal substances. In the first screening, we isolated medicinal substances that were found to increase the survival rate of wild-type animals by 10%. In the second screening, we retested the cold tolerance using the chemical substances isolated by the first screening, 1–3 times under the same experimental conditions, to further isolate the chemicals that increased the survival rate by 20%.

As mentioned earlier, *C. elegans* exhibits cold tolerance depending on the cultivation temperature. Animals cultivated at 25 °C cannot survive at 2 °C; however, animals cultivated at 15 °C can survive at 2 °C^13,40^. With the objective of identifying novel genes that control cold tolerance, we established an experimental system taking advantage of the phenomenon of cold tolerance in wild-type *C. elegans* strains exposed to various medicinal substances. The wild-type strains were cultivated at 22.5 °C in a nematode growth medium (NGM) impregnated with approximately 4000 medicinal substances (Fig. 1a,b). After cultivation at 22.5 °C for 3 days, the animals were rapidly exposed to a low-temperature stimulus of 3 °C (Fig. 1a). Although several chemicals caused larval arrest, these animals were transferred directly to 3 °C in the larval stages in the first screening. Wild-type strains cultivated at 22.5 °C could not survive at 3 °C, whereas those cultivated in NGM impregnated with various medicinal substances exhibited abnormal increments of cold tolerance. As a first screening, medicinal substances that enhanced the survival rate of wild-type strains by > 10% were isolated (Fig. 1b), because almost all wild-type animals cultivated at ≥ 22 °C cannot survive at 2 °C^13^. Among the 4000 compounds, animals cultivated in NGM supplemented with LMB, CPT, or dihydrolotenone exhibited enhancedcold tolerance in the first screening. Because these three compounds induced larval arrest, animals grown at 22.5 °C were cultivated to young adults in NGM without added chemicals, after which they were transferred to new NGM with chemicals in the second screening. Moreover, in the second screening, we selected chemicals that increased cold tolerance by > 20%, a more severe criterion than that in the first screening. After exposing the animals grown at 22.5 °C to 3 °C, the survival rates of LMB- or CPT-treated animals increased by > 20% (Fig. 1b), whereas the animals exposed to dihydrolotenone did not exhibit higher survival rates. Therefore, we proceeded with the experiment to investigate the effects of LMB or CPT on cold tolerance.

### Effects of LMB or CPT on development and cold tolerance

**Figure 2:**
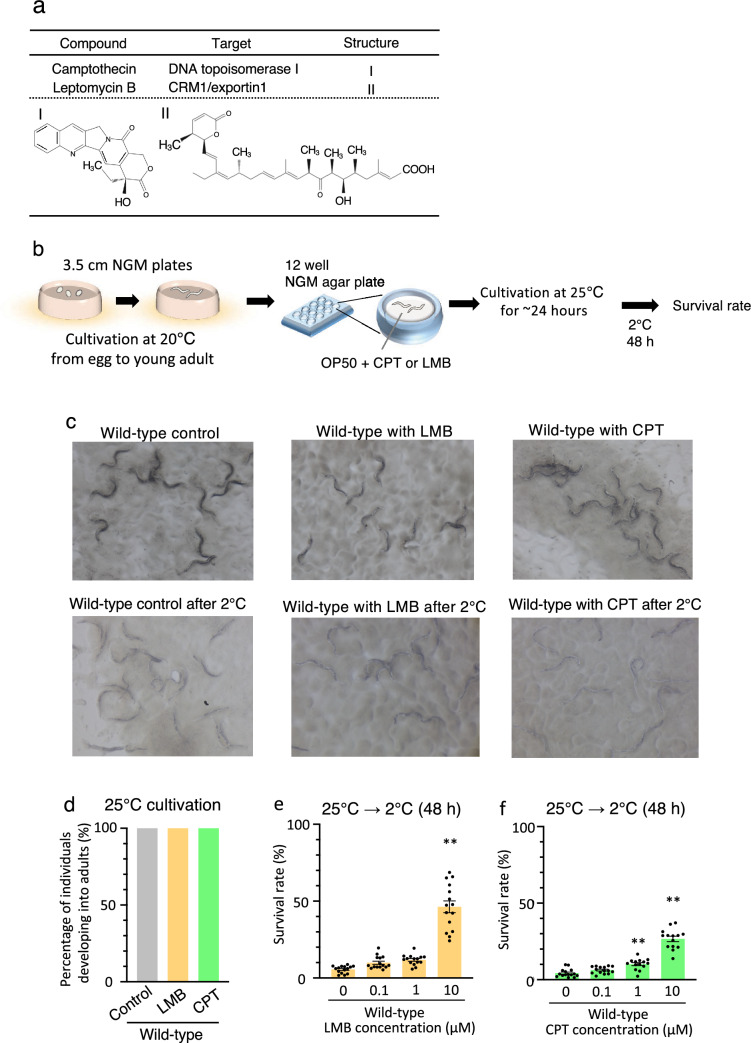
Cold tolerance analysis of animals exposed to LMB and CPT. (**a**) The target molecules and chemical structures of CPT and LMB and the phenotypes of chemical-exposed animals. CPT interacts with the topoisomerase I–DNA complex to inhibit DNA replication. LMB in conjunction with leucine-rich NES inhibits the nuclear export of proteins. (**b**) After cultivation at 20 °C from the egg to young adult stage in 3.5-cm-diameter NGM plates, the animals were transferred to 12-well NGM plates containing the chemical substances and cultivated at 25 °C for ~ 24 h. After treatment with chemicals, the animals were exposed to cold stimuli at 2 °C. (**c**) Animals were cultivated at 20 °C until young adult stage and transferred to NGM plates containing 10 µM LMB or 10 µM CPT at 25 °C for ~ 24 h. (Top): Animals were observed without cold stimulation. (Bottom): Animals were observed after cold stimulation. (**d**) Animals were cultivated at 20 °C until young adult stage and transferred to NGM plates with LMB or CPT at 25 °C for ~ 24 h. The animals were allowed to develop into adults under LMB or CPT exposure. Wild-type control n = 300, wild-type with CPT n = 293, wild-type with LMB n = 325. Comparisons were performed using Fisher’s exact test. *P < 0.05, **P < 0.01. (**e, f**) Cold tolerance was dependent upon the concentration of the chemical substances. Wild-type animals were cultivated on agar medium plates impregnated with 0, 0.1, 1, and 10 µM chemical substances and exposed to 2 °C for 48 h. The survival rates of animals under treatment with both CPT or LMB were significantly enhanced by the addition of 10 µM chemicals compared to that of animals without treatment of chemicals. Number of assays ≥ 14. Error bar indicates SEM. Comparisons were performed using Dunnett’s test. *P < 0.05, **P < 0.01. (SEM: standard error of the mean).

Previous studies have reported that CPT inhibits DNA replication by interacting with the DNA–topoisomerase I complex^31,32^, and LMB inhibits the nuclear export of proteins by inhibiting CRM1/exportin1, which encodes a nuclear transporter^26–29^(Fig. 2a). Leptomycins belong to the unsaturated, branched-chain fatty acids with 3-lactone rings at the end, as evident from the structural analysis (Fig. 2a)^23^. As the eggs of *C. elegans* strains cultivated in NGM impregnated with LMB or CPT failed to hatch, or their growths were arrested at the larval stage, we considered that LMB and CPT prevent adult development during embryogenesis or larval stages. Hence, *C. elegans* were allowed to proliferate from the egg stage to the young adult stage in NGM without chemicals, then transferred to NGM impregnated with LMB or CPT and incubated for approximately 24 h (Fig. 2b). Thus, it was evident that although *C. elegans* failed to hatch from eggs or that the larvae failed to survive in the drug-infused medium, in the adult stage, they could survive in this medium for 24 h (Fig. 2c [top] and d), suggesting that LMB or CPT does not inhibit the survival of *C. elegans* at the adult stage.

To further investigate the effects of LMB or CPT on cold tolerance in the adult stage, *C. elegans* cultivated at 25 °C for appropriately 24 h after exposure to either LMB or CPT in the young adult stage were subjected to a 2 °C cold stimulus (Fig. 2b). To determine the concentrations at which LMB or CPT affects the cold tolerance phenotype, wild-type strains of *C. elegans* cultivated at 25 °C were exposed to 0, 0.1, 1, and 10 µM LMB or CPT, respectively, and were then subjected to a cold stimulus of 2 °C. The wild-type strains cultivated at 25 °C failed to survive at 2 °C (Fig. 2e [0 µM] and Fig. 2f [0 µM]); however, the wild-type strains exposed to 10 µM LMB and 1 and 10 µM CPT exhibited a significant increase in cold tolerance (Fig. 2c [bottom], Fig. 2e [10 µM] and Fig. 2f [1 and 10 µM]). These findings suggest that at least 10 µM LMB or 1 µM CPT affects the cold tolerance of *C. elegans*.

### Exploring genes that affect cold tolerance under LMB exposure

**Figure 3:**
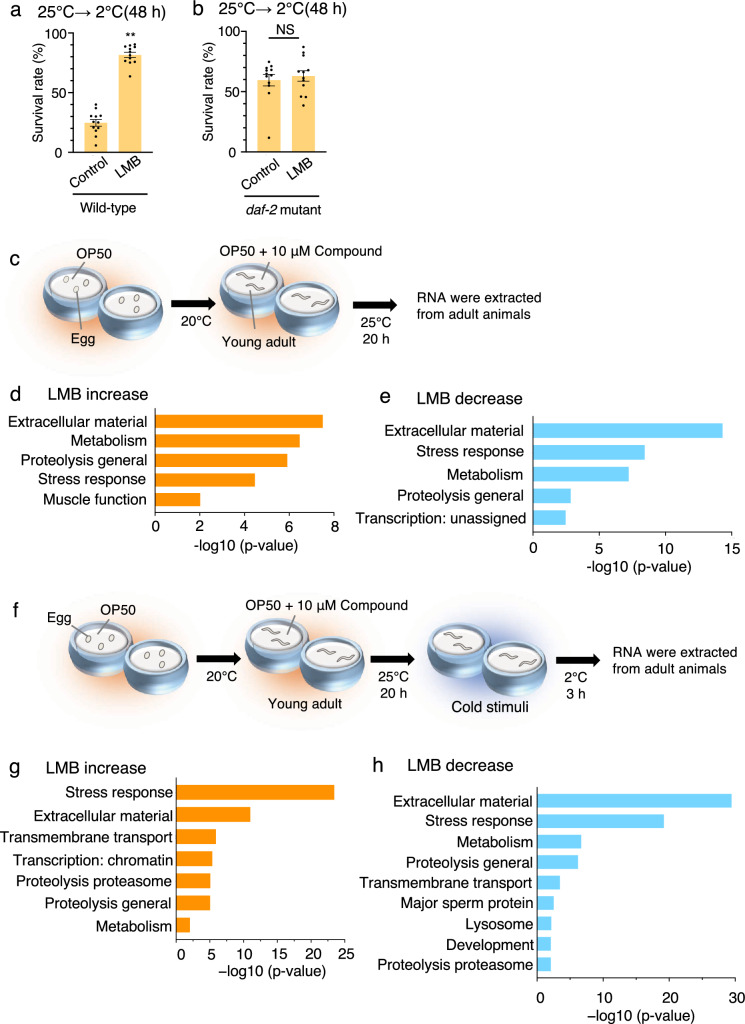
RNA sequencing analysis of wild-type animals treated with LMB. (**a**) Wild-type animals cultivated at 25 °C were exposed to 2 °C for 48 h. Wild-type control animals shown in Fig. 3a are the same as those shown in Fig. 5a, because these experiments were conducted simultaneously. Number of assays was ≥ 12. Error bar indicates SEM. Comparisons were performed using the unpaired t-test (Welch). *P < 0.05, **P < 0.01. (**b**) *daf-2* mutants cultivated at 25 °C were exposed to 2 °C for 48 h. *daf-2* mutant controls shown in Fig. 3b are the same as those shown in Fig. 5b, because these experiments were conducted simultaneously. Number of assays was ≥ 12. Error bar indicates SEM. Comparisons were performed using the unpaired t-test (Welch). *P < 0.05, **P < 0.01. (SEM: Standard error of the mean). (**c**) RNA sequencing analysis was performed to compare the RNA expression levels of wild-type strains with and without treatment of chemicals, for screening downstream molecules affected by the chemicals. Wild-type animals were cultivated at 20 °C from the egg to young adult stage and then transferred to chemical-added plates and cultivated at 25 °C for 20 h. The expression levels of RNA extracted from animals cultivated on chemical-added plates were compared with those from animals cultivated on NGM plates without chemicals. (**d**) When wild-type animals were cultivated at 25 °C under LMB exposure for 20 h, the expression levels of genes involved in extracellular material, metabolism, proteolysis general, stress response, and muscle function were significantly increased. The differentially expressed genes from the LMB treatment (logFC > 1, Wald test, P < 0.01) were submitted to WormCat analysis for annotation and visualization of enrichment data. The categories showing p-value < 0.01 were indicated (Fisher’s exact test). The X-axis represents the − log10 *P*-value. (**e**) When wild-type animals were cultivated at 25 °C under LMB exposure for 20 h, the expression levels of genes involved in extracellular material, stress response, metabolism, proteolysis general and transcription: unassigned were decreased. The differentially expressed genes from the LMB treatment (logFC <  − 1, Wald test, P < 0.01) were submitted to WormCat analysis for annotation and visualization of enrichment data. The categories showing p-value < 0.01 were indicated (Fisher’s exact test). The X-axis represents the − log10 p-value. (**f**) To screen for downstream molecules affected by the chemicals under cold stimuli, the RNA expression levels of wild-type strains were compared with and without treatment of chemicals under 2 °C exposure. After cultivation at 20 °C from the egg to young adult stage, the wild-type animals were transferred to chemical-added plates. After cultivation at 25 °C for 20 h, animals were exposed to 2 °C for 3 h. The expression levels of RNA extracted from animals cultivated on chemical-added plates were compared with those from animals cultivated on NGM plates without chemicals. (**g**) When wild-type animals were exposed to cold stimuli at 2 °C with LMB exposure, the expression levels of genes involved in stress response, extracellular material, transmembrane transport, transcription: chromatin, proteolysis proteasome, proteolysis general, and metabolism were increased. The differentially expressed genes from the LMB treatment (logFC > 1, Wald test, *P* < 0.01) were submitted to WormCat analysis for annotation and visualization of enrichment data. The categories showing *p*-value < 0.01 were indicated (Fisher’s exact test). The X-axis represents the − log10 *p*-value. (**h**) When wild-type animals were exposed to cold stimuli at 2 °C with LMB exposure, the expression levels of genes involved in extracellular material, stress response, metabolism, proteolysis general, transmembrane transport, major sperm protein, lysosome, development, and proteolysis proteasome were decreased. The differentially expressed genes from the LMB treatment (logFC <  − 1, Wald test, P < 0.01) were submitted to WormCat analysis for annotation and visualization of enrichment data. The categories showing *p*-value < 0.01 were indicated (Fisher’s exact test). The X-axis represents the − log10 p-value.

We have previously reported that the thermosensory neuron ASJ secretes insulin after receiving temperature information, which is received by the insulin receptor DAF-2 in the intestine^13^. To investigate the pathway in which LMB affects cold tolerance at 25 °C, we examined the genetic relationship between LMB and the insulin receptor *daf-2*, which is involved in cold tolerance to 25 °C. LMB increased cold tolerance in the wild-type animals (Fig. 3a). The cold tolerance of *daf-2* mutants treated with LMB and that of *daf-2* mutant control were similar, indicating that LMB failed to affect cold tolerance in the *daf-2* mutant (Fig. 3b). These results indicate that the genetic pathway inhibited by LMB is located upstream of the *daf-2*-mediated pathway involved in the cold tolerance of animals cultivated at 25 °C.

To identify the genes affected by LMB, which regulate cold tolerance, we performed mRNA sequencing to screen for genes with altered expression levels after exposure to LMB. Because 10 µM LMB increased the cold tolerance of animals cultivated at 25 °C (Fig. 2e), we extracted RNA from wild-type strains cultivated at 25 °C without exposure to chemicals and from wild-type strains cultivated on plates treated with 10 µM LMB and then compared the expression levels of all genes in *C. elegans* (Fig. 3c). LMB-exposed *C. elegans* strains exhibited significantly increased expression of genes associated with extracellular material, metabolism, proteolysis general, stress response, and muscle function (Fig. 3d) and a downregulation of genes associated with extracellular material, stress response, metabolism, proteolysis general, and transcription: unassigned (Fig. 3e). To determine the genes whose expression levels were altered by LMB under the 2 °C cold stimulus, we extracted RNA from animals cultivated at 25 °C in LMB-added medium and then exposed to 2 °C for 3 h (Fig. 3f). *C. elegans* strains exposed to the cold stimulus after cultivation in LMB-infused medium exhibited a significantly increased expression of genes related to stress response, extracellular material, transmembrane transport, transcription: chromatin, proteolysis proteasome, proteolysis general, and metabolism (Fig. 3g) and a decreased expression of genes related to extracellular material, stress response, metabolism, proteolysis general, transmembrane transport, major sperm protein, lysosome, development, and proteolysis proteasome (Fig. 3h). As there was no significant increase in the expression of genes associated with transmembrane transport, transcription: chromatin, and proteolysis proteasome (Fig. 3d and g), and there was no significant decrease in the expression of genes associated with transmembrane transport, major sperm protein, lysosome, development, and proteolysis proteasome in the animals cultivated at 25 °C (Fig. 3e and h), these genes could have been altered by the cold stimulus and their expression could have been affected by LMB exposure. A total of 209 genes exhibited increased expression levels, and 162 genes exhibited decreased expression levels after LMB exposure in the absence of low-temperature stimulation (Supplementary Table 1 and 2). A total of 1204 genes exhibited increased expression levels, and 561 genes exhibited decreased expression levels compared with genes in animals cultivated in NGM without LMB when subjected to the cold stimulus after LMB exposure. Of the genes with altered expression levels, 38 or 8 genes showed increased or decreased expression levels, respectively, in animals transferred to 2 °C without LMB exposure, suggesting that these are not the genes whose expression was altered by LMB exposure. These findings indicate that the expression levels of 1166 or 553 genes are increased or decreased, respectively, when exposed to the cold stimulus of 2 °C, and that this altered expression was due to LMB exposure (Supplementary Tables 3 and 4).

**Figure 4:**
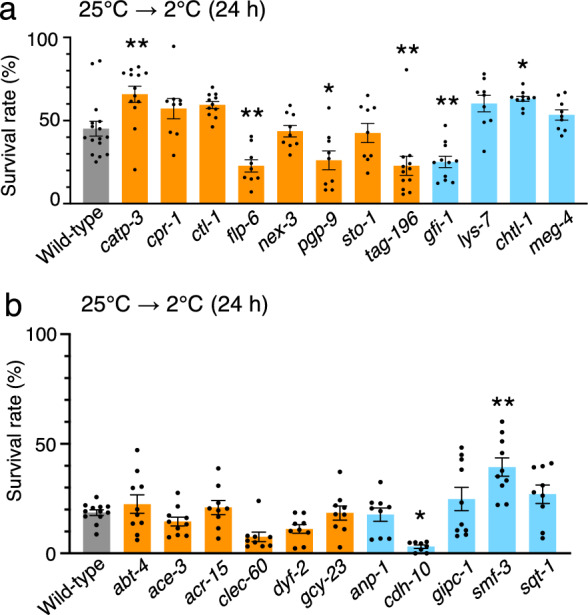
Cold tolerance of mutants defective in genes whose expression was altered by LMB. (**a**) Cold tolerance of mutants defective in genes whose expression levels were altered by LMB exposure. Number of assays was ≥ 9. Error bar indicates SEM. Comparisons were performed using Dunnett’s test. *P < 0.05, **P < 0.01. Wild-type animals shown in Fig. 4a are the same as those shown in Fig. 6a, because these experiments were conducted simultaneously. Orange or blue bars indicate upregulated or downregulated genes, respectively. (**b**) Cold tolerance of mutants defective in genes whose expression levels were altered by cold stimuli at 2 °C under LMB exposure. Number of assays was ≥ 8. Error bar indicates SEM. Comparisons were performed using Dunnett’s test. *P < 0.05, **P < 0.01. Wild-type animals shown in Fig. 4b are the same as those shown in Fig. 6b, because these experiments were conducted simultaneously. Orange or blue bars indicate upregulated or downregulated genes, respectively.

To screen the genes involved in cold tolerance, we evaluated the cold tolerance of mutants defective in the genes that had altered expression levels due to LMB exposure in the 25 °C cultivation condition (Fig. 3c and 4a). When the mutants were grown for 3 days at 25 °C, we evaluated the cold tolerance of those mutants that could grow into adults in sufficient numbers (≥ 30 animals) to allow for cold tolerance analysis. The survival rate of wild-type animals was ~ 45%. In contrast, we observed significantly greater cold tolerance in mutants defective in *catp-3* (cation-transporting ATPase) and *chtl-1* (choline transporter) and lower cold tolerance in mutants defective in *flp-6* (FMRF-like peptide), *pgp-9* (P-glycoproteins related), *tag-196* (cathepsin F), and *gfi-1* (protein with ET module) (Fig. 4a).

We next evaluated the cold tolerance of mutants defective in genes with altered expression levels after exposure to the 2 °C cold stimulus after having been cultivated at 25 °C in the presence of LMB (Fig. 3f). Wild-type control animals cultivated at 25 °C and exposed to 2 °C for 24 h exhibited ~ 20% survival rates. Mutants defective in *smf-3* (divalent cation transporter) exhibited increased cold tolerance, whereas mutants defective in *cdh-10* (cadherin family) exhibited decreased cold tolerance (Fig. 4b). Although mutants defective in *flp-6*, *pgp-9*, *tag-196*, *gfi-1*, and *cdh-10* were characterized by decreased cold tolerance, ~ 99% of every individual mutant survived when cultivated at 25 °C without cold stimulation, indicating that these mutants exhibited decreased survival rates upon cold stimulation at 2 °C (Supplementary Fig. 1). The results of mRNA sequencing and cold tolerance test suggested that LMB affects the cold tolerance of wild-type animals by altering the expression of genes, whose mutants exhibited abnormal cold tolerance.

### Exploring genes that affect cold tolerance under CPT exposure

**Figure 5:**
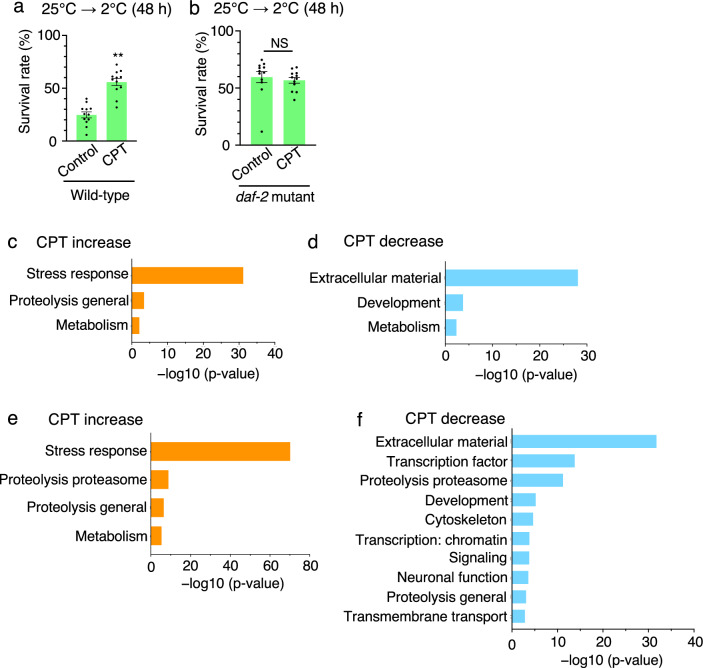
RNA sequencing analysis of wild-type animals treated with CPT. (**a**) Wild-type animals cultivated at 25 °C were exposed to 2 °C for 48 h. Wild-type control animals shown in Fig. 5a are the same as those shown in Fig. 3a, because these experiments were conducted simultaneously. Number of assays was ≥ 12. Error bar indicates SEM. Comparisons were performed using the using the unpaired t-test (Welch). *P < 0.05, **P < 0.01. (**b**) *daf-2* mutants cultivated at 25 °C were exposed to 2 °C for 48 h. *daf-2* mutant controls shown in Fig. 5b are the same as those shown in Fig. 3b, because these experiments were conducted simultaneously. Number of assays was ≥ 12. Error bar indicates SEM. Comparisons were performed using the unpaired t-test (Welch). *P < 0.05, **P < 0.01. (**c**) When wild-type animals were cultivated at 25 °C under CPT exposure for 20 h, the expression levels of genes involved in stress response, proteolysis general and metabolism were significantly increased. The differentially expressed genes from the CPT treatment (logFC > 1, Wald test, P < 0.01) were submitted to WormCat analysis for annotation and visualization of enrichment data. The categories showing p-value < 0.01 were indicated (Fisher’s exact test). The X-axis represents the − log10 p-value. (**d**) When wild-type animals were cultivated at 25 °C under CPT exposure for 20 h, the expression levels of genes involved in extracellular material, development and metabolism were decreased. The differentially expressed genes from the CPT treatment (logFC <  − 1, Wald test, P < 0.01) were submitted to WormCat analysis for annotation and visualization of enrichment data. The categories showing p-value < 0.01 were indicated (Fisher’s exact test). The X-axis represents the − log10 p-value. (**e**) When wild-type animals were exposed to cold stimuli at 2 °C with CPT exposure, the expression levels of genes involved in stress response, proteolysis proteasome, proteolysis general, and metabolism were increased. The differentially expressed genes from the CPT treatment (logFC > 1, Wald test, P < 0.01) were submitted to WormCat analysis for annotation and visualization of enrichment data. The categories showing p-value < 0.01 were indicated (Fisher’s exact test). The X-axis represents the − log10 p-value. (**f**) When wild-type animals were exposed to cold stimuli at 2 °C with CPT exposure, the expression levels of genes involved in extracellular material, transcription factor, proteolysis proteasome, development, cytoskeleton, transcription: chromatin, signaling, neuronal function, proteolysis general, and transmembrane transport were decreased. The differentially expressed genes from the CPT treatment (logFC <  − 1, Wald test, P < 0.01) were submitted to WormCat analysis for annotation and visualization of enrichment data. The categories showing p-value < 0.01 were indicated (Fisher’s exact test). The X-axis represents the − log10 p-value.

Cold tolerance in the intestine is regulated by the insulin receptor DAF-2 when animals are cultivated at 25 °C^13^. We examined the genetic relationship between the cold tolerance pathway affected by CPT and the insulin receptor *daf-2*, which is involved in cold tolerance to 25 °C. Although CPT positively influenced the cold tolerance in the wild-type population (Fig. 5a), the cold tolerance of the *daf-2* mutant exposed to CPT was similar to that of the *daf-2* mutant control (Fig. 5b), suggesting that cold tolerance in the *daf-2* mutant is not affected by CPT. This result indicates that the genetic pathway inhibited by CPT is located upstream of the *daf-2*-mediated pathway involved in the cold tolerance of animals cultivated at 25 °C.

As mentioned earlier, 10 µM CPT increased the cold tolerance of animals cultivated at 25 °C (Fig. 2f). To examine the genes whose expression was altered by CPT, we compared the expression levels of all genes between animals cultivated on plates treated with 10 µM CPT and animals cultivated on plates without CPT (Fig. 3c). We observed significantly higher expression of genes related to stress response, proteolysis general, and metabolism in animals exposed to CPT (Fig. 5c), whereas the expression of genes related to extracellular material, development, and metabolism (Fig. 5d) reduced. We extracted RNA from animals cultivated at 25 °C in CPT-added medium and then exposed to 2 °C to examine the genes whose expression levels were altered by CPT under the 2 °C cold stimulus (Fig. 3f). We observed that the genes related to stress response, proteolysis proteasome, proteolysis general, and metabolism were upregulated (Fig. 5e), whereas genes related to extracellular material, transcription factor, proteolysis proteasome, development, cytoskeleton, transcription: chromatin, signaling, neuronal function, proteolysis general, and transmembrane transport downregulated (Fig. 5f). As we found no significant increase in the expression of genes related to proteolysis proteasome (Fig. 5c and e) and no significant decrease in the expression of genes related to transcription factor, proteolysis proteasome, cytoskeleton, transcription: chromatin, signaling, neuronal function, proteolysis general, and transmembrane transport (Fig. 5d and f) in the absence of the cold stimulus, the expression of these genes could be affected by CPT exposure and the cold stimulus. Although CPT exposure led to increased expression levels of 166 genes and decreased expression levels of 117 genes in the absence of cold stimulation (Supplementary Tables 5 and 6), the expression levels of 717 genes increased and those of 741 genes decreased at 2 °C under CPT exposure compared with those in animals not exposed to CPT. Of the genes with altered expression levels, 22 or 7 genes showed increased or decreased expression levels, respectively, in animals transferred to 2 °C without CPT exposure, suggesting that these are not the genes whose expression was altered by CPT exposure. These data indicated that CPT affected the expression levels of 695 or 734 genes under the cold stress of 2 °C (Supplementary Tables 7 and 8).

**Figure 6:**
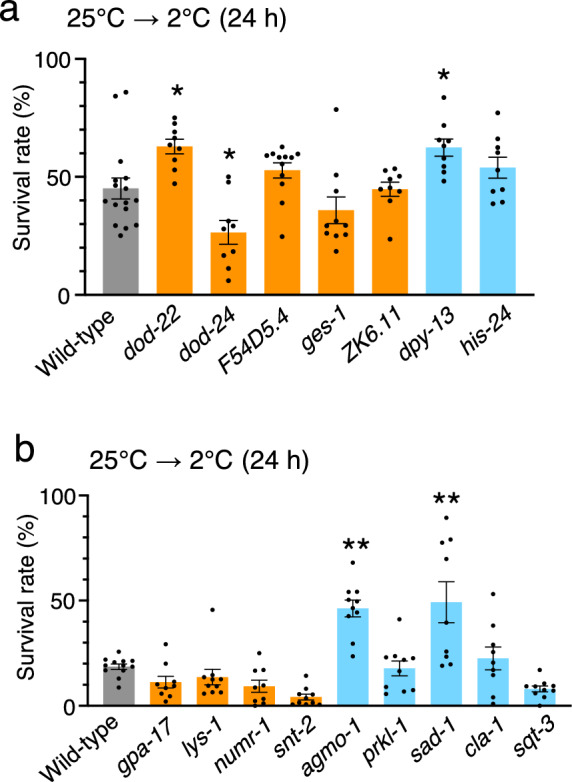
Exploring cold tolerance pathways affected by CPT. (**a**) Cold tolerance of the mutants defective in genes whose expression levels were altered by CPT exposure. Number of assays was ≥ 9. Error bar indicates SEM. Comparisons were performed using Dunnett’s test. *P < 0.05, **P < 0.01. Wild-type shown in Fig. 6a is the same as that shown in Fig. 4a, because these experiments were conducted simultaneously. Orange or blue bars indicate upregulated or downregulated genes, respectively. (**b**) Cold tolerance of the mutants defective in genes whose expression levels were altered by cold stimuli at 2 °C under CPT exposure. Number of assays was ≥ 9. Error bar indicates SEM. Comparisons were performed using Dunnett’s test. *P < 0.05, **P < 0.01. Wild-type shown in Fig. 6b is the same as that shown in Fig. 4b, because these experiments were conducted simultaneously. Orange or blue bars indicate upregulated or downregulated genes, respectively.

To explore the genes responsible for the increase in the cold tolerance of animals under CPT exposure, we evaluated the cold tolerance of mutants defective in genes with altered expression in animals cultivated at 25 °C under CPT exposure (Fig. 3c). We evaluated the cold tolerance of mutants that could grow in sufficient numbers of adult animals at 25 °C. The cold tolerance of animals cultivated and grown from the egg to adult stage at 25 °C, and then exposed to 2 °C, significantly improved in mutants defective in *dod-22* (downstream of *daf-16*) and *dpy-13* (cuticle collagens) compared with that of wild-type animals (Fig. 6a). The mutant defective in *dod-24* (downstream of *daf-16*) exhibited decrement of cold tolerance (Fig. 6a), although it survived at 25 °C without the cold stimulus of 2 °C (Supplementary Fig. 1).

We evaluated the cold tolerance of mutants defective in genes with altered expression levels after exposure to the 2 °C cold stimulus after being grown at 25 °C under CPT exposure (Fig. 3f) by cultivating the animals at 25 °C and then exposing them to 2 °C for 24 h. We observed that the cold tolerance was significantly higher in mutants defective in *agmo-1* (alkylglycerol monooxygenase) and *sad-1* (serine/threonine kinase) (Fig. 6b). These data suggest that CPT affects the cold tolerance of wild-type animals by altering the expression of genes, whose mutants exhibited abnormal cold tolerance.

## Discussion

Temperature is an important environmental information, and organisms are equipped with biological mechanisms to adapt to rapid temperature changes. In this study, we established an experimental system involving screening for genes that regulate cold tolerance in the presence of some chemicals. After screening ~ 4000 drugs, we observed that the anticancer drugs LMB and CPT affected cold tolerance.

LMB acts as an inhibitor of CRM1/exportin1 and also inhibits the nuclear export of proteins with NES^26–29^. Previous studies have used mass spectrometry to reveal the proteins transported by CRM1/exportin1 from the nucleus to the cytoplasm^41,42^. To identify the target genes for LMB that regulate cold tolerance, we compared the genes reported to regulate the cytoplasm–nucleus transport by *xpo-1*^42^ with the currently recognized genes, whose expression was altered when subjected to cold stimulation at 2 °C in the presence of LMB. The 20 genes whose expression was altered by LMB exposure overlapped with genes involved in the cytoplasm–nucleus transport by *xpo-1* (Supplementary Table 9)^41,42^. These data indicate that LMB could inhibit the cytoplasm–nucleus transport of the genetic products, whose expression is altered by cold stimulation via *xpo-1*.

**Figure 7:**
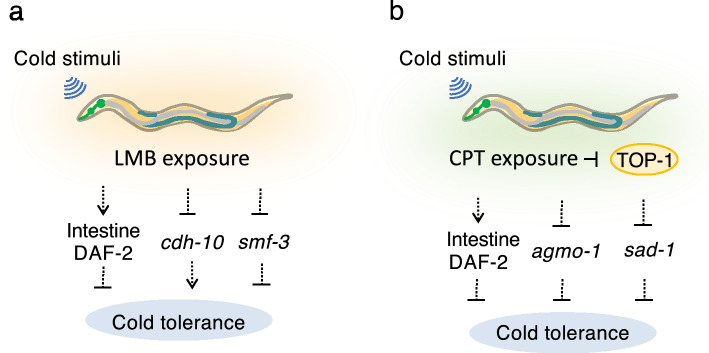
Exploring cold tolerance pathways affected by CPT. (**a**) Molecular model of the cold tolerance pathway affected by LMB. (**b**) Molecular model of the cold tolerance pathway affected by CPT.

Cold tolerance is regulated by the insulin receptor DAF-2 in the intestine^13^. LMB increased the cold tolerance of wild-type animals, but not of *daf-2* mutants (Fig. 3a and b). These observations indicated that LMB affected cold tolerance upstream of DAF-2 (Fig. 7a). The expression levels of *cdh-10* (cadherin) and *smf-3* (divalent cation transporter) markedly decreased after the 2 °C cold stimulation under LMB exposure compared with that in animals not exposed to LMB. The expression of these genes remained unchanged in the *daf-2* mutant^14^. These findings indicate that *cdh-10* and *smf-3* are not the genes that regulate cold tolerance by changing the expression downstream of the insulin receptor DAF-2 in the intestine (Fig. 7a).

CPT acts as an inhibitor of topoisomerase I and thus inhibits DNA replication^31^. The expression levels of genes involved in stress responses significantly increased at 2 °C after CPT exposure (Fig. 5e). Specifically, we observed markedly increased expression levels of genes categorized as pathogen, detoxification, C-type lectin, and heavy metal in this study (Supplementary Table10). These results propose a model in which CPT inhibits the activity of the *tpo-1* gene product, which encodes the sole homolog of topoisomerase I in the *C. elegans* genome. This causes DNA damage through abnormal maintenance of DNA topology via topoisomerase I, which finally causes an increased expression of genes involved in the stress response.

Wild-type animals cultivated at 25 °C under CPT exposure exhibited higher cold tolerance than wild-type control animals (Fig. 5a), whereas the cold tolerance of *daf-2* mutant animals remained unaffected (Fig. 5b), suggesting that CPT affects cold tolerance in the upstream pathway of DAF-2 in the intestine (Fig. 7b). The expression levels of *agmo-1* (alkylglycerol monooxygenase) and *sad-1* (serine/threonine kinase) significantly decreased at 2 °C after CPT exposure compared with that in animals not exposed to CPT. As previously reported, the expression levels of these genes remained unaltered in the *daf-2* mutant^14^. These results indicate that *agmo-1* and *sad-1* are not the genes that regulate cold tolerance by changing the expression downstream of the insulin receptor DAF-2 in the intestine (Fig. 7b).

When animals were exposed to CPT or LMB during the egg-laying period, they laid many dead eggs, suggesting that LMB or CPT inhibited normal germ cell formation. According to a previous study, DAF-16 belongs to the FOXO family of transcription factors and is localized in the nucleus in intestinal cells, and the lifespan also increases in germline-ablated animals^43^. DAF-16/FOXO regulates cold tolerance downstream of the insulin receptor DAF-2, which functions in the intestine^13^, and the mutants of *dod-22* and *dod-24*, which function downstream of *daf-16*, exhibited abnormal cold tolerance. This finding suggests the possibility that chemicals affect the germline, altering the expression of genes downstream of *daf-16* in the intestine and consequently influencing cold tolerance.

We investigated the cold tolerance of mutants defective in genes whose expression was altered by the chemicals. The loss-of-function mutants of *gfi-1*, *chtl-1*, *cdh-10*, *smf-3*, *dpy-13*, *agmo-1*, and *sad-1* exhibited abnormal cold tolerance, suggesting that the reduced function or defects in these genes cause abnormal cold tolerance. Although the expression levels of *catp-3*, *flp-6*, *pgp-9*, *tag-196*, *dod-22*, and *dod-24* were increased by the chemicals, the loss-of-function mutants exhibited abnormal cold tolerance. If the drug-induced expression of these genes is the cause of the abnormal cold tolerance, a balance in the expression of these genes may be necessary to achieve normal cold tolerance.

Through our experimental system aimed at screening drugs responsible for cold tolerance in *C. elegans,* we identified the anticancer drugs LMB or CPT as those affecting cold tolerance, after screening almost 4000 drugs. We comprehensively investigated genes with altered expression levels due to LMB or CPT exposure by performing RNA analysis. The genes regulating cold tolerance were screened by conducting a cold tolerance test on mutants of the genes with varied expression following CPT or LMB exposure. Meanwhile, the target genes of the drugs or the pathways regulating cold tolerance could not be comprehensively identified in this study. In the future, an experimental system combining chemical agents and the phenotype of cold tolerance could be set up for a comprehensive screening of genes controlling cold tolerance at low cost and in a short time.

## Materials and methods

### Strains

The culturing and handling *C. elegans* was performed in accordance with standard procedures described by Brenner^44^. All animal experiments related to this study were approved by the Animal Review Committee of Konan University(K-23-20). The experiments were performed in accordance with ARRIVE guidelines. The *C. elegans* strains used in this study were: N2 Bristol England; CB1370 *daf-2(e1370)*; CB6738 *lys-7(ok1384)*; CB184 *dpy-13(e184)*; FX00830 *pgp-9(tm830)*; JH3248 *meg-4(ax2081)*; JM104*1 ges-1(ca6ca7)*; VC913 *nex-3(gk385)*; RB1067 *his-24(ok1024)*; RB1197 *ctl-1(ok1242)*; RB1262 *cpr-1(ok1344)*; RB1415 *catp-3(ok1612)*; RB1467 *chtl-1(ok1695)*; RB1573 *dod-22(ok1918)*; RB1656 *F54D5.4(ok2046)*; RB1994 *dod-24(ok2629)*; RB2016 *gfi-1(ok2669)*; RB939 *tag-196(ok822)*; *sto-1(tm1503)*; VC2324 *flp-6(ok3056)*; *ZK6.11(ok3738)*; *RB817 abt-4(ok633)*; PR1300 *ace-3(dc2)*; RB1172 *acr-15(ok1214)*; *clec-60 (tm2319)*; SP1234 *dyf-2(m160)*; RB924 *gcy-23(ok797)*; RB804 *anp-1(ok608)*; RB2160 *cdh-10(ok2920)*; BA1091 *gipc-1(hc192)*; RB1074 *smf-3(ok1035)*; BE103 *sqt-1(sc103)*; RB1800 *gpa-17(ok2334)*; RB1893 *lys-1(ok2445)*; RB1749 *numr-1(ok2239)*; *snt-2(tm1711)*; LC144 *agmo-1(e3016)*; RB2346 *prkl-1(ok3182)*; CX5156 *sad-1(ky289)*; VC387 *cla-1(ok618)*; BE63 *sqt-3(sc63).*

### Cold tolerance test for drug screening

A chemical library for the drug screening was prepared in association with Takeda Pharmaceutical Company. Culture plates for drug screening were prepared as follows: 150 µl of NGM^44^ was added per well to 96-well plates. Enriched *Escherichia coli* OP50 and compounds were added to the NGM agar to achieve a final concentration of 40 μM chemicals, and were then dried for ~ 4–8 h, Several adult animals (P0) were placed onto the NGM plates in the form of 224 × 224 mm square dishes and incubated for ~ 6 days to ensure the collection of sufficient number of eggs for evaluating cold tolerance, until F1 animals reached the adult stage and the food supply was depleted. The F1 adult animals were harvested by washing with S-basal buffer^45^. To obtain the eggs, the collected F1 animals were dissolved in 3.5 ml egg-isolation solution (hypochlorous acid 1 mL + water 2.15 mL + 10N NaOH 0.35 mL). Following the removal of adult carcasses by repeated centrifugation and washing, the solution containing only F2 eggs was transferred to 96-wells plates containing NGM and compounds, so that each well contained ~ 30–60 eggs. Following cultivation at 22.5 °C for 3 days, the plates were transferred to a freezer at 3 °C for 48 h after initially placing them on ice for 15 min, The number of live and dead animals were counted after ~ 2–12 h after removing the plates from the refrigerator after exposure to the cold stimulus, to calculate survival rates. For the secondary screening for the drugs that inhibited animal growth, animals initially cultured in NGM medium without added chemicals until they grew into young adults, were placed into a 3-cm dish containing chemical-added NGM, and maintained for 24 h. The plates after being placed on ice for 15 min, were transferred to a freezer at 3 °C for 48 h. After exposure to the cold stimulus, the number of live and dead animals were counted to calculate survival rates.

### Cold tolerance assay

Cold tolerance tests were conducted as described previously^13,20,46^. Following cultivation in 2% (w/v) agar NGM plates containing *Escherichia coli* OP50 in 3.5-cm-diameter plastic dishes, well-conditioned adults (P0) were incubated for ~ 20 h until they had laid ~ 100 eggs. The growth of F1 animals was synchronized by removing the P0 animals. The F1 animals were incubated from the egg to adult stage at 25 °C for ~ 50 h. The plates were then immediately chilled on ice for 20 min, following which they were transferred to a 2 °C refrigerator (CRB-41A, Hitachi, Japan) for 24 h. Following exposure to cold stimulus, the assay plates were incubated at 15 °C until movement was spotted in the nematodes. Survival rates were calculated by counting the number of live and dead animals. More than three plates were used per day to conduct the cold tolerance and temperature acclimation tests. The live and dead animals were counted on plates containing more than 30 nematodes. All data were obtained by assaying the results from at least three independent days.

### Cold tolerance assay with chemicals

Animals were cultivated on 3.5-cm-diameter plastic plates containing 2% (w/v) agar NGM plates with *Escherichia coli* OP50. Single or several well-conditioned adult animals (P0) were placed at the desired temperature and incubated for 15–20 h or 3–5 h, until they had laid ~ 100 eggs. P0 animals were then removed to synchronize the growth of F1 animals. The F1 animals were incubated from the egg to and allowed to grow to the young adult stage at 20 °C for ~ 67 h. Using the M9 buffer, the animals were collected in a 1.5-ml tube and were washed at least two times with the M9 buffer. Chemical stock (10 mM, 1 mM or 0.1 mM) prepared in pure dimethyl sulfoxide (DMSO), was then added to the NGM agar to achieve a final concentration of 10 μM, 1 μM or 0.1 μM chemical with 0.1% DMSO as the solvent. The NGM agar without chemicals was prepared by adding DMSO to NGM to a final concentration of 0.1% DMSO and was used as control. The animals that had been washed with the M9 buffer were transferred to NGM plates containing either CPT or LMB, and were allowed to dry for approximately 10 min. The plates were placed in a light-shielding container. In the cold tolerance test of wild-type animals exposed to 0, 0.1, 1, or 10 µM LMB or CPT, the animals after cultivation at 20 °C from egg to young adult in NGM without chemicals were transferred to 25 °C after exposure to drugs and were kept for 24 hours. Since *daf-2(e1379)* undergoes dauer arrest at 25° C, wild-type or *daf-2(e1379)* animals were cultivated at 15 °C from the egg to young adult stage, and then were transferred to 25 °C for 24 h after exposure to drugs for the cold tolerance test for cultivation at 25 °C. The plates were transferred to a 2 °C refrigerator (CRB-41A, Hitachi, Japan) for 48 h after first chilling them on ice for 20 min. Following exposure to cold stimuli, the assay plates were incubated at 15 °C until movement was observed in the nematodes. The number of live and dead animals were counted to determine the survival rates. Each cold tolerance and temperature acclimation test was conducted using more than three plates per day. Plates containing more than 30 nematodes were used for the counting of the live and dead animals. All data were obtained by assaying the results from at least three independent days.

### RNA sequencing analysis

Total RNA was extracted from wild-type N2 strains treated with either CPT or LMB. Animals were cultivated in 2% (w/v) agar NGM plates containing *E. coli* OP50 in 3.5-cm-diameter plastic dishes. Several well-conditioned adults (P0) were incubated until they had laid ~ 100 eggs. P0 animals were then removed to synchronize the growth of F1 animals, which were then incubated from the egg to young adult stage at 20 °C for ~ 67 h. Young adult animals were collected using M9 buffer from the cultivation plates, placed into 1.5-ml tubes, and washed twice with M9 buffer. The washed animals were further transferred to NGM plates impregnated with 10 µM LMB or CPT. Control animals were maintained on 3.5-cm plates without transferring to NGM supplemented with LMB or CPT. After collecting and washing the animals twice with M9 buffer (22 mM KH_2_PO_4_, 42 mM Na_2_HPO_4_, 85 mM NaCl, and 1 mM MgSO_4_) following cultivation at 25 °C for 20 h, the animals were transferred to 2 °C for 3 h. The Maxwell RSC Simply RNA tissue kit (Promega; AS1340) with Maxwell RSC Instrument (Promega; AS4500) was used to extract total RNA in accordance with the manufacturer’s instructions. Sequencing libraries were prepared using a TruSeq Stranded mRNA Sample Prep Kit (Illumina) in accordance with the manufacturer’s instructions. After quantifying the libraries using Bioanalyzer (Agilent) they were sequenced on an Illumina NovaSeq 6000 instrument to a depth of at least 13.3 mega read 60 million reads using 150-bp paired end reads. RNA-seq data have been deposited in the DRA of DDBJ with the accession number DRA015100 DRA016968. CLC Genomics Workbench 21 (Qiagen, Germany) was used to analyze the raw RNA-seq readings.

The online tool for annotation and visualization, WormCat was used for gene set enrichment analysis^47^. The gene sets whose expression were significantly upregulated (*p* < 0.01, logFC > 1) or downregulated (*p* < 0.01, logFC < -1) by treatment of CPT or LMB were categorized. The gene set was mapped to the *C. elegans* “Whole genome v2,” following the standard WormCat Flow analysis parameters.

### Analysis of the effects of CPT and LMB on development

Following cultivation in 2% (w/v) agar NGM plates containing *Escherichia coli* OP50 in 3.5-cm-diameter plastic dishes, the single or several well-conditioned adults (P0) were placed at the desired temperature and incubated until they had laid ~ 100 eggs for 15–20 h or 3–5 h. After removing the P0 animals, the F1 animals were incubated from the egg to young adult stage at 15 °C for ~ 120 h or 20 °C for ~ 67 h. The animals were collected in a 1.5-ml tubes using M9 buffer, and were then washed at least two times with the M9 buffer. DMSO was added to NGM to obtain a final concentration of 0.1% DMSO to obtain NGM without chemicals for the control experiment. The washed animals were transferred to NGM plates to which either 10 µM CPT or 10 µM LMB had been added, and were dried up for approximately 10 min. Light-shielding containers were used to cultivate animals at each temperature for 24 h. Percentage of individuals developing into adults were calculated by counting the number of adult animals with egg, and those without eggs or immotile.

### Statistical analysis

All error bars in the figures indicate SEM (standard error of the mean). All statistical analyses were conducted using parametric tests, that is, Dunnett’s test, or the unpaired t-test (Welch) using the assumption that all cold tolerance data follow normal distribution. Multiple comparisons were performed using one-way analysis of variance, with comparisons tested using Dunnett’s test. The leftmost groups of the bar graphs were compared with other groups by performing Dunnett’s test. Comparisons between two groups were performed using the unpaired t-test (Welch). The statistical analysis for Fig. 2d and Supplementary Fig. 1 was performed using Fisher’s exact test (*P < 0.05, **P < 0.01). The tests were performed using Mac statistical analysis version 3 (Esumi, Japan) and GraphPad Prism 9 (GraphPad Software, USA). GraphPad Prism 9 (GraphPad Software, USA) was used for preparing all graphs.

### Ethical approval

All animal experiments related to this study were approved by the Animal Review Committee of Konan University(K-23–20). The experiments were performed in accordance with ARRIVE guidelines. All animal experiments conducted in this research were performed in accordance with the Japanese Act on Welfare and Management of Animals (Act No. 105 of October 1, 1973; latest revisions Act No. 51 of June 2, 2017, Effective June 1, 2018). The experimental protocols were approved by the Institutional Animal Care and Use Committees of Konan University.

### Supplementary Information

Supplementary Information 1.

Supplementary Information 2.

Supplementary Information 3.

## Data Availability

RNA-seq data have been deposited and released in the DDBJ BioProject with the accession number PRJDB14678 (https://ddbj.nig.ac.jp/resource/bioproject/PRJDB14678). The datasets generated during in this study can be accessed from the corresponding author on reasonable request.
