# Supplementary material for: Screening for cold tolerance genes in C. elegans, whose expressions are affected by anticancer drugs camptothecin and leptomycin B
Source: Sci Rep. 2024 Mar 5;14:5401. doi: 10.1038/s41598-024-55794-z (PMC10914781; doi:10.1038/s41598-024-55794-z)
Supplement: Supplementary file 2 — Supplementary Information 2. [file 41598_2024_55794_MOESM2_ESM.pdf]

Supplementary Information for

Screening for cold tolerance genes in *C. elegans*, whose expressions are affected by anticancer drugs camptothecin and leptomycin B

Misaki Okahata[1-4,#], Natsumi Sawada[2,#], Kenji Nakao[5], Akane Ohta[2-4*], Atsushi Kuhara[2-4, 6,*]

[1]Graduate School of Frontier Biosciences, Osaka University Suita, Osaka, Japan
[2]Graduate School of Natural Science, Konan University, Kobe, Hyogo, Japan
[3]Faculty of Science and Engineering, Konan University, Kobe, Hyogo, Japan
[4]Institute for Integrative Neurobiology, Konan University, Kobe, Hyogo, Japan
[5]Biomolecular Research Laboratories, Pharmaceutical Research Division, Takeda Pharmaceutical Company Limited, Fujisawa, Japan
[6]PRIME, AMED, Japan Agency for Medical Research and Development, Tokyo, Japan

[#]These authors contributed equally to this study
*Correspondence and requests should be addressed to A.K. and A.O.
o_akaneiro@me.com (A.O.), atsushi_kuhara@me.com (A.K.)

This file includes:

Supplementary Figures 1-4
Supplementary Results and Discussion
Supplementary Methods
Supplemental References
Supplementary Table 1-10

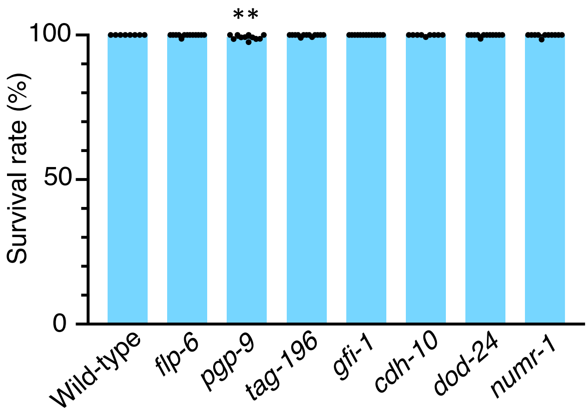

Supplementary Figure1

The survival rate without cold stimulation of mutants showing an abnormality of decreased cold tolerance at 25°C cultivation condition.

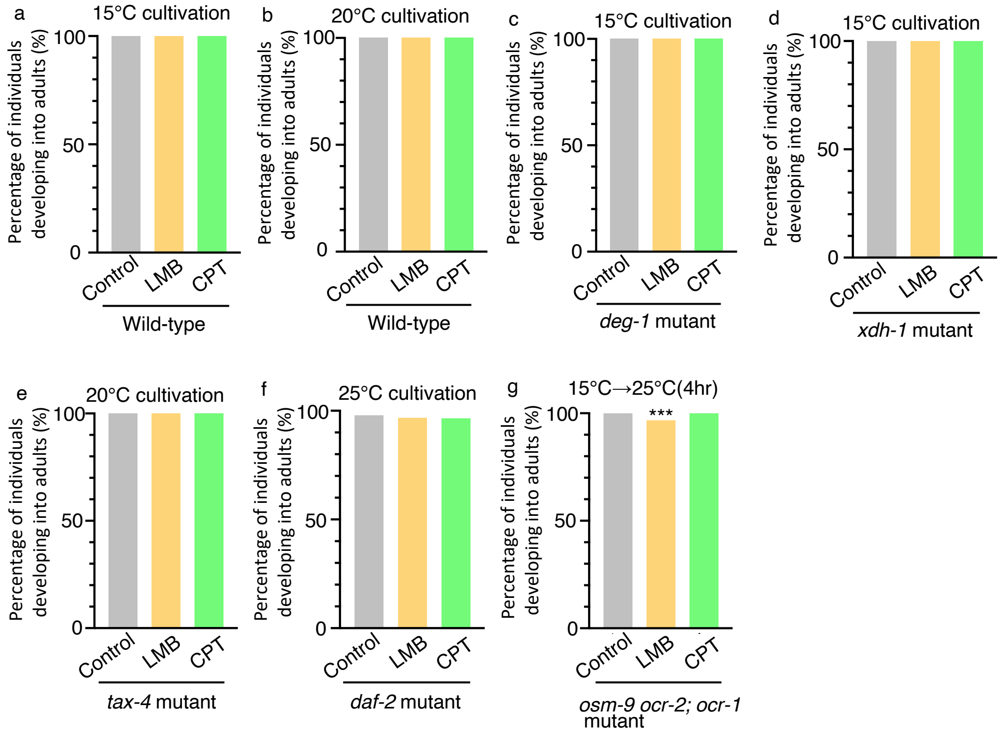

Supplementary Figure2

(a-g) After cultivating from eggs to young adults at the desired temperature, the animals were exposed to the chemical for 24 hours. Comparisons were performed using Fisher's exact test. *P < 0.05, **P < 0.01.

(a) Percentage of wild-type adults under chemical exposure at 15°C cultivation. Wild-type control n = 122, wild-type with CPT n = 153, wild-type with LMB n = 103

(b) Percentage of wild-type adults under chemical exposure at 20°C cultivation. Wild-type control n = 222, wild-type with CPT n = 202, wild-type with LMB n = 209

(c) Percentage of *deg-1* mutant adults under chemical exposure at 15°C cultivation. *deg-1* mutant controls n = 403, *deg-1* mutants with CPT n = 457, *deg-1* mutants with LMB n = 287

(d) Percentage of *xdh-1* mutant adults under chemical exposure at 15°C cultivation. *xdh-1* mutant controls n = 330, *xdh-1* mutants with CPT n = 359, *xdh-1* mutants with LMB n = 340

(e) Percentage of *tax-4* mutant adults under chemical exposure at 20°C cultivation. *tax-4* mutant controls n = 124, *tax-4* mutants with CPT n = 125, *tax-4* mutants with LMB n = 106

(f) Animals were cultivated from eggs to young adults at 15°C and then transferred to 25°C for 24 hours. Percentage of individual *daf-2* mutant animals that developed into adults when they were exposed to the chemical after cultivation at 25°C for 24 hours. *daf-2* mutant controls n = 141, *daf-2* mutants with CPT n = 175, *daf-2* mutants with LMB n = 156

(g) Percentage of *osm-9 ocr-2; ocr-1* mutant adults under chemical exposure at 15°C to 24°C for 4 h. *osm-9 ocr-2; ocr-1* mutant controls n = 374, *osm-9 ocr-2; ocr-1*mutants with CPT n = 358, *osm-9 ocr-2; ocr-1* mutants with LMB n = 252

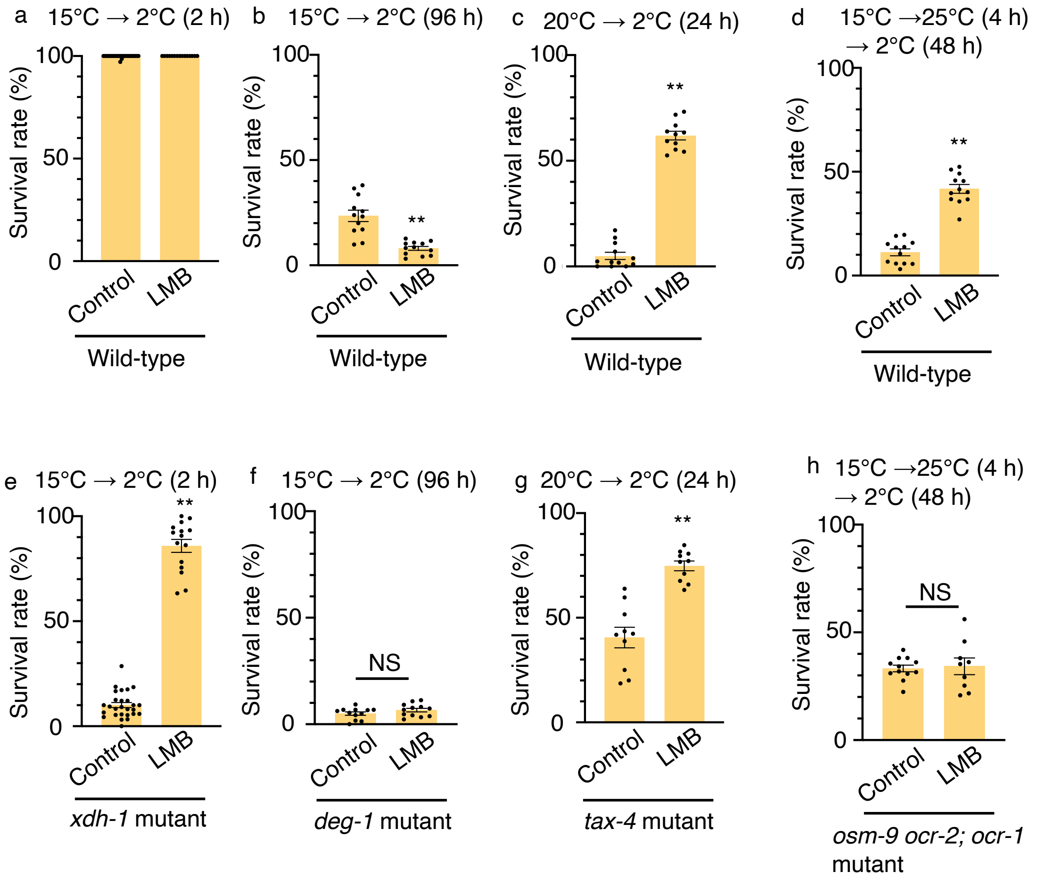

Supplementary Figure3

(a) Wild-type animals cultivated at 15°C were exposed to 2°C for 2 h. Wild-type control animals were subjected to cold tolerance test in NGM without chemical addition. Wild-type control animals shown in Supplementary Figure 3a are the same as those shown in Supplementary Figure 4a, because these experiments were conducted simultaneously. Number of assays ≥ 15. Error bar indicates SEM. Comparisons were performed using the unpaired t-test (Welch). *P < 0.05, **P < 0.01.

(b) Wild-type animals cultivated at 15°C were exposed to 2°C for 96 h. Wild-type control animals shown in Supplementary Figure 3b are the same as those shown in Supplementary Figure 4b, because these experiments were conducted simultaneously. Number of assays ≥ 12. Error bar indicates SEM. Comparisons were performed using the unpaired t-test (Welch). *P < 0.05, **P < 0.01.

(c) Wild-type animals cultivated at 20°C were exposed to 2°C for 24 h. Wild-type control animals shown in Supplementary Figure 3c are the same as those shown in Supplementary Figure 4e, because these experiments were conducted simultaneously. Number of assays ≥ 11. Error bar indicates SEM. Comparisons were performed using the unpaired t-test (Welch). *P < 0.05, **P < 0.01.

(d) 15°C-cultivated wild-type animals were transferred to 25°C for 4 h, and exposed to 2°C for 48 h. Wild-type control animals shown in Supplementary Figure 3d are the same as those shown in Supplementary Figure 4f, because these experiments were conducted simultaneously. Number of assays ≥ 12. Error bar indicates SEM. Comparisons were performed using the unpaired t-test (Welch). *P < 0.05, **P < 0.01.

(e) *xdh-1* mutant cultivated at 15°C were exposed to 2°C for 2 h. *xdh-1* mutant controls were subjected to cold tolerance test in NGM without chemical addition. *xdh-1* mutant controls shown in Supplementary Figure 3e are the same as those shown in Supplementary Figure 4g, because these experiments were conducted simultaneously. Number of assays ≥ 15. Error bar indicates SEM. Comparisons were performed using the unpaired t-test (Welch). *P < 0.05, **P < 0.01.

(f) *deg-1* mutants cultivated at 15°C were exposed to 2°C for 96 h. *deg-1* mutant controls shown in Supplementary Figure 3f are the same as those shown in Supplementary Figure 4h, because these experiments were conducted simultaneously. Number of assays ≥ 12. Error bar indicates SEM. Comparisons were performed using the unpaired t-test (Welch). *P < 0.05, **P < 0.01.

(g) *tax-4* mutants cultivated at 20°C were exposed to 2°C for 24 h. *tax-4* mutant controls shown in Supplementary Figure 3g are the same as those shown in Supplementary Figure 4j, because these experiments were conducted simultaneously. Number of assays ≥ 10. Error bar indicates SEM. Comparisons were performed using the unpaired t-test (Welch). *P < 0.05, **P < 0.01.

(h) 15°C-cultivated *osm-9 ocr-2; ocr-1* mutants were transferred to 25°C for 4 h, and exposed to 2°C for 48 h. *osm-9 ocr-2; ocr-1* mutants shown in Supplementary Figure 3h are the same as those shown in Supplementary Figure 4k, because these experiments were conducted simultaneously. Number of assays ≥ 9. Error bar indicates SEM. Comparisons were performed using the unpaired t-test (Welch). *P < 0.05, **P < 0.01.

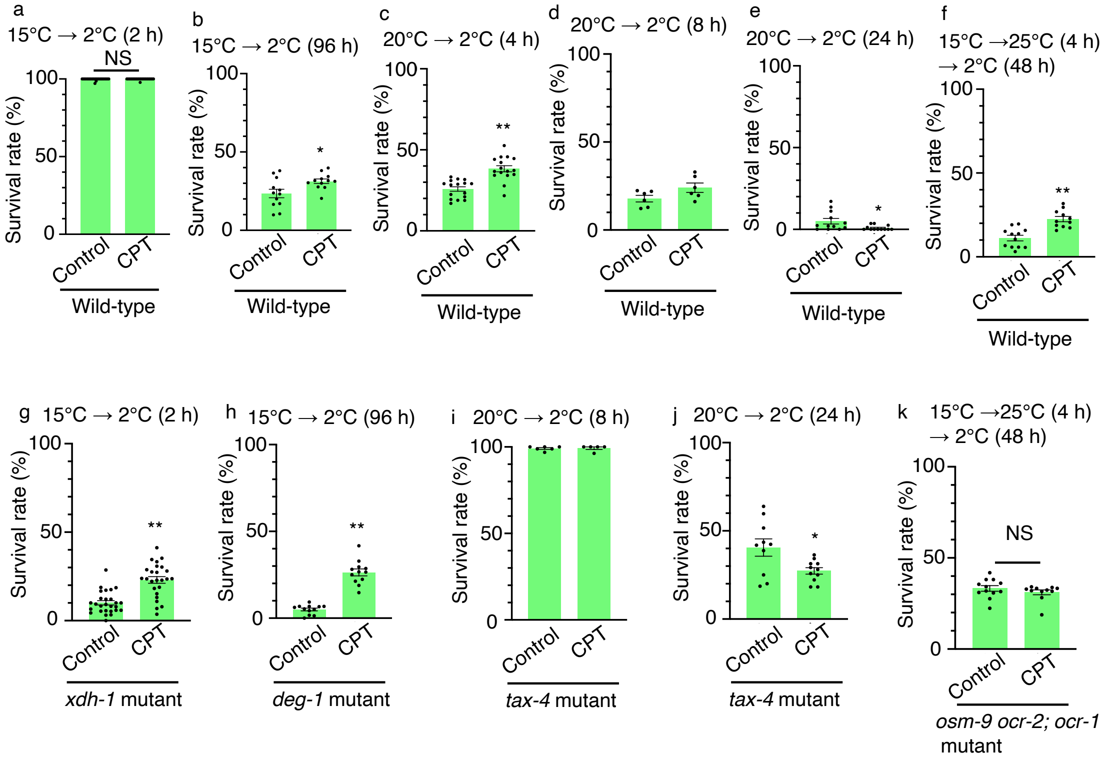

Supplementary Figure4

(a) Wild-type animals cultivated at 15°C were exposed to 2°C for 2 h. Wild-type control animals were subjected to cold tolerance test in NGM without chemical addition. Wild-type controls shown in Supplementary Figure4a are the same as those shown in Supplementary Figure3a, because these experiments were conducted simultaneously. Number of assays ≥ 25. Error bar indicates SEM. Comparisons were performed using the using the unpaired t-test (Welch). *P < 0.05, **P < 0.01.

(b) Wild-type animals cultivated at 15°C were exposed to 2°C for 96 h. Wild-type control animals shown in Supplementary Figure4b are the same as those shown in Supplementary Figure3b, because these experiments were conducted simultaneously. Number of assays ≥ 12. Error bar indicates SEM. Comparisons were performed using the using the unpaired t-test (Welch). *P < 0.05, **P < 0.01.

(c) Wild-type animals cultivated at 20°C were exposed to 2°C for 4 h. Number of assays ≥ 16. Error bar indicates SEM. Comparisons were performed using the unpaired t-test (Welch). *P < 0.05, **P < 0.01.

(d) Wild-type animals cultivated at 20°C were exposed to 2°C for 8 h. Number of assays ≥ 5. Error bar indicates SEM. Comparisons were performed using the unpaired t-test (Welch). *P < 0.05, **P < 0.01.

(e) Wild-type animals cultivated at 20°C were exposed to 2°C for 24 h. Wild-type control animals shown in Supplementary Figure4e are the same as those shown Supplementary

Figure3c, because these experiments were conducted simultaneously. Number of assays ≥ 12. Error bar indicates SEM. Comparisons were performed using the unpaired t-test (Welch). *P < 0.05, **P < 0.01.

(f) 15°C-cultivated wild-type animals were transferred to 25°C for 4 h, and exposed to 2°C for 48 h. Wild-type controls shown in Supplementary Figure4f are the same as those shown in Supplementary Figure3d, because these experiments were conducted simultaneously. Number of assays ≥ 11. Error bar indicates SEM. Comparisons were performed using the unpaired t-test (Welch). *P < 0.05, **P < 0.01.

(g) *xdh-1* mutants cultivated at 15°C were exposed to 2°C for 2 h. *xdh-1* mutant controls were subjected to cold tolerance test in NGM without chemical addition. *xdh-1* mutant controls shown in Supplementary Figure4g are the same as those shown in Supplementary Figure3e, because these experiments were conducted simultaneously. Number of assays ≥ 25. Error bar indicates SEM. Comparisons were performed using the unpaired t-test (Welch). *P < 0.05, **P < 0.01.

(h) *deg-1* mutants cultivated at 15°C were exposed to 2°C for 96 h. *deg-1* mutant controls shown in Supplementary Figure4h are the same as those shown in Supplementary Figure3f, because these experiments were conducted simultaneously. Number of assays ≥ 12. Error bar indicates SEM. Comparisons were performed using the unpaired t-test (Welch). *P < 0.05, **P < 0.01.

(i) *tax-4* mutant cultivated at 20°C were exposed to 2°C for 8 h. Number of assays ≥ 5. Error bar indicates SEM. Comparisons were performed using the unpaired t-test (Welch). *P < 0.05, **P < 0.01.

(j) *tax-4* mutants cultivated at 20°C were exposed to 2°C for 24 h. *tax-4* mutant controls shown in Supplementary Figure4j are the same as those shown in Supplementary Figure3g, because these experiments were conducted simultaneously. Number of assays ≥ 10. Error bar indicates SEM. Comparisons were performed using the unpaired t-test (Welch). *P < 0.05, **P < 0.01.

(k) 15°C-cultivated *osm-9 ocr-2; ocr-1* mutants were transferred to 25°C for 4 h, and exposed to 2°C for 48 h. *osm-9 ocr-2; ocr-1* mutants shown in Supplementary Figure4k are the same as those shown in Supplementary Figure3h, because these experiments were conducted simultaneously. Number of assays ≥ 11. Error bar indicates SEM. Comparisons were performed using the unpaired t-test (Welch). *P < 0.05, **P < 0.01.

Supplementary Results and Discussion

Various genes regulating cold tolerance and temperature acclimation have been previously isolated by us, and we have also proposed their intracellular mechanisms or inter-tissue networks that depend on cultivation temperature [1-6]. To investigate the genetic relationship between the known pathways involved in regulation of cold tolerance or temperature acclimation, and the target site of LMB or CPT, the effect of LMB or CPT was examined in various mutants defective with respect to the genes responsible for regulating cold tolerance or temperature acclimation. Before conducting cold tolerance or temperature acclimation assay, we confirmed that the mutants were not killed by LMB or CPT (Supplementally Fig. 2a-g). The animals were cultivated and allowed to grow from eggs to young adults on NGM plates devoid of LMB or CPT, following which they were transferred to NGM with LMB for 24 hours. In all mutant strains, more than 95% of individual animals succeeded to develop into adults, even following LMB or CPT exposure (Supplementally Fig. 2a-g). We thus measured the cold tolerance of these adult animals which were exposed to LMB, excluding the animals that had developmental abnormalities.

Although LMB was identified as a chemical affecting cold tolerance at 25°C, LMB also affected cold tolerance or temperature acclimation of wild-type cultivated at different cultivation temperature (Supplementary Fig. 3a-d). The DEG/ENaC-type thermoreceptor DEG-1 and xanthine dehydrogenase XDH-1 regulate cold tolerance in the animals cultivated at 15°C [6]. The temperature information received by DEG-1 in sensory neuron ASG transmits to the interneurons AVH and AIN, wherein XDH-1 acts as a modulator of the neuronal activity of AVH and AIN[6,7]. Animals cultivated at 20°C transduce the signal regarding the temperature information in the thermosensory neuron ASJ, through the cGMP-dependent channel TAX-4, a negative factor for cold tolerance[1]. The TRPV channels OSM-9, OCR-2, and OCR-1 regulate temperature acclimation from 15°C. to 25°C in the thermosensory neuron ADL[3-5]. We measured cold tolerance or temperature acclimation of mutants defective in genes involved in these pathways under LMB exposure (Supplementary Figure 3e-h). The cold tolerance of *xdh-1* mutant or *tax-4* mutants was increased by LMB exposure (Supplementary Figure 3e and g), suggesting that LMB might affect genes involved in cold tolerance downstream of XDH-1 in AVH and AIN or TAX-4 in ASJ. The temperature acclimation of *osm-9 ocr-2; ocr-1*mutant was not affected by LMB exposure (Supplementary Figure 3h), indicating that LMB might impact temperature acclimation upstream of OSM-9, OCR-2, and OCR-1 in ADL.

CPT affected not only 25 °C, but also cold tolerance at 15 and 20 °C or cold acclimation from 15 °C to 25 °C (Supplementary Figure 4a-f). We measured cold tolerance or temperature acclimation of mutants defective in genes involved in cold tolerance pathways

under CPT exposure (Supplementary Figure 4g-k). CPT increased cold tolerance in both *xdh-1* and *deg-1* mutants in animals cultivated at 15°C (Supplementary Figure 4g and h) or decreased cold tolerance in *tax-4* mutant in animals cultivated at 20°C (Supplementary Figure 4j). This data implies that CPT possibly affects cold tolerance downstream of XDH-1 and DEG-1 in ASG or TAX-4 in ASJ. The temperature acclimation of *osm-9 ocr-2; ocr-1*mutant was not affected by CPT exposure (Supplementary Figure 4k), indicating that CPT might affect the molecular pathway for temperature acclimation upstream of OSM-9, OCR-2, and OCR-1 in ADL.

Supplementary Methods

Strains

The culturing and handling *C. elegans* was performed in accordance with standard procedures described by Brenner [8]. The *C. elegans* strains used in this study were: N2 Bristol England; KHR067 *xdh-1(ok3234)*; *TU38 deg-1 (u38)*; FK127 *tax-4(p678)*; FG125 *osm-9(ky10) ocr-2(ak47); ocr-1(ak46)*

Cold tolerance and temperature acclimation assay with chemicals

Animals were cultivated on 3.5-cm-diameter plastic plates containing 2% (w/v) agar NGM plates with *Escherichia coli* OP50. Single or several well-conditioned adult animals (P0) were placed at the desired temperature and incubated for 15–20 h or 3–5 h, until they had laid ~100 eggs. P0 animals were then removed to synchronize the growth of F1 animals. The F1 animals were incubated from the egg to and allowed to grow to the young adult stage at 15°C for ~120 h or 20°C for ~67 h. Using the M9 buffer, the animals were collected in a 1.5-ml tube and were washed at least two times with the M9 buffer. Chemical stock (10 mM, 1 mM or 0.1 mM) prepared in pure dimethyl sulfoxide (DMSO), was then added to the NGM agar to achieve a final concentration of 10 µM, 1 µM or 0.1 µM chemical with 0.1% DMSO as the solvent. The NGM agar without chemicals was prepared by adding DMSO to NGM to a final concentration of 0.1% DMSO and was used as control. The animals that had been washed with the M9 buffer were transferred to NGM plates containing either CPT or LMB, and were allowed to dry for approximately 10 min. The plates were placed in a light-shielding container. In the cold tolerance test of wild-type animals exposed to 0, 0.1, 1, or 10 µM LMB or CPT, the animals after cultivation at 20°C from egg to young adult in NGM without chemicals were transferred to 25°C after exposure to drugs and were kept for four hours. In the cold tolerance test of wild-type, *xdh-1(ok3234)*, *deg-1(u38)*, or *tax-4(p678)*, the

animals were cultivated from egg to young adult stage at the desired temperature. Since *daf-2(e1379)* undergoes dauer arrest at 25° C, wild-type or *daf-2(e1379)* animals were cultivated at 15°C from the egg to young adult stage, and then were transferred to 25°C for 24 h after exposure to drugs for the cold tolerance test for cultivation at 25°C. In the temperature acclimation test of *ocr-2(ak47) osm-9(ky10)*; *ocr-1(ak46)*, the animals were cultivated at 15°C for ~20 h after exposure to drugs, and then transferred to 15°C for 4 h. After cultivation at each temperature, the animals were exposed to chemicals and transferred to desired temperature for 24 h. The plates were transferred to a 2°C refrigerator (CRB-41A, Hitachi, Japan) for 2, 4, 24, 48, or 96 h after first chilling them on ice for 20 min. Following exposure to cold stimuli, the assay plates were incubated at 15°C until movement was observed in the nematodes. The number of live and dead animals were counted to determine the survival rates. Each cold tolerance and temperature acclimation test was conducted using more than three plates per day. Plates containing more than 30 nematodes were used for the counting of the live and dead animals. All data were obtained by assaying the results from at least three independent days.

Supplemental References

1. Ohta, A., Ujisawa, T., Sonoda, S. & Kuhara, A. Light and pheromone-sensing neurons regulates cold habituation through insulin signalling in Caenorhabditis elegans. *Nat Commun*. **5**, 4412 (2014).
2. Sonoda, S., Ohta, A., Maruo, A., Ujisawa, T. & Kuhara, A. Sperm Affects Head Sensory Neuron in Temperature Tolerance of Caenorhabditis elegans. *Cell Rep*. **16**, 56-65 (2016).
3. Ujisawa, T. *et al.* Endoribonuclease ENDU-2 regulates multiple traits including cold tolerance via cell autonomous and nonautonomous controls in Caenorhabditis elegans. *Proc Natl Acad Sci U S A*. **115**, 8823-8828 (2018).
4. Okahata, M., Wei, A. D., Ohta, A. & Kuhara, A. Cold acclimation via the KQT-2 potassium channel is modulated by oxygen in Caenorhabditis elegans. *Sci Adv*. **5**, eaav3631 (2019).
5. Ohnishi, K. *et al.* OSM-9 and OCR-2 TRPV channels are accessorial warm receptors in Caenorhabditis elegans temperature acclimatisation. *Sci Rep*. **10**, 18566 (2020).
6. Takagaki, N. *et al.* The mechanoreceptor DEG-1 regulates cold tolerance in Caenorhabditis elegans. *EMBO Rep*. **21**, e48671 (2020).

7. Okahata, M., Motomura, H., Ohta, A. & Kuhara, A. Molecular physiology regulating cold tolerance and acclimation of Caenorhabditis elegans. *Proc Jpn Acad Ser B Phys Biol Sci*. **98**, 126-139 (2022).
8. Brenner, S. The genetics of Caenorhabditis elegans. *Genetics*. **77**, 71-94 (1974).

Supplementary Table1

RNA sequencing analysis of animals cultivated at 25°C for 20 hours under LMB exposure

(logFC>1 p<0.01)

Name	Max group means	Fold change	Log fold change	P-value
alh-4	111.33	2.15	1.11	5.21E-04
aqp-2	325.81	2.83	1.50	3.08E-09
asp-1	2324.78	2.01	1.01	2.05E-10
atn-1	50.15	2.75	1.46	2.79E-04
cah-3	80.73	5.84	2.55	2.39E-04
ccg-1	346.83	2.41	1.27	4.79E-06
cle-1	13.89	6.51	2.70	1.39E-03
cpi-1	1119.64	2.39	1.26	5.36E-07
col-8	451.79	2.91	1.54	1.13E-07
col-19	277.44	2.84	1.50	7.21E-07
col-20	520.30	3.63	1.86	3.46E-09
col-42	216.09	2.08	1.05	2.87E-03
col-95	1134.62	2.12	1.08	1.57E-07
col-106	1274.42	2.10	1.07	1.10E-07
col-119	1365.36	2.27	1.18	1.47E-10
col-124	518.14	3.48	1.80	3.74E-10
col-140	1449.78	2.63	1.39	3.83E-11
col-165	57.16	4.09	2.03	6.10E-03
col-178	802.97	2.04	1.03	1.24E-06
col-181	1601.19	2.02	1.01	8.43E-07
col-184	1210.60	2.34	1.23	1.54E-10
cpn-3	554.97	2.52	1.33	5.30E-06
cpn-4	132.97	2.66	1.41	7.10E-03
cpr-1	249.54	2.60	1.38	3.70E-05
cpr-3	320.39	21.18	4.40	4.57E-14
cpr-4	2571.10	14.07	3.81	0
cpr-5	210.30	2.04	1.03	7.36E-03
cpz-2	116.42	2.57	1.36	1.89E-04
ctl-1	80.93	2.17	1.12	8.00E-03
dhs-14	57.71	5.96	2.57	2.47E-03
dlg-1	45.34	3.42	1.77	5.11E-06
dpy-14	450.74	3.81	1.93	2.41E-12
dpy-17	276.12	4.22	2.08	1.65E-08
far-3	81.91	8.46	3.08	1.83E-03
flp-6	150.19	3.22	1.69	4.28E-04
gem-4	28.19	3.74	1.90	7.65E-03

gln-3	337.29	2.52	1.33	1.36E-08
gst-5	90.26	10.44	3.38	6.01E-04
gst-24	227.27	155.97	7.29	2.04E-03
hbl-1	27.78	12.92	3.69	1.07E-05
hch-1	24.46	7.28	2.86	4.07E-03
hil-7	113.95	4.42	2.15	2.01E-04
hpd-1	257.57	2.35	1.23	7.80E-06
hsp-43	319.53	3.53	1.82	1.66E-08
lys-3	107.96	11.99	3.58	9.13E-05
lys-4	439.14	22.65	4.50	2.50E-12
nex-1	456.82	2.16	1.11	2.29E-06
nex-2	62.84	3.75	1.91	6.67E-05
nex-3	94.63	3.95	1.98	2.03E-04
nfm-1	17.83	7.33	2.87	7.52E-03
nmy-1	47.73	2.05	1.04	1.90E-04
odc-1	53.98	7.05	2.82	3.48E-04
pab-2	58.03	2.07	1.05	9.50E-04
pcp-3	23.39	3.88	1.95	1.79E-03
pfn-2	222.28	2.87	1.52	2.74E-05
pgp-9	14.50	3.30	1.72	8.10E-03
pmp-4	25.98	6.51	2.70	1.33E-03
pqn-31	43.88	14.61	3.87	2.32E-03
skr-3	144.61	10.97	3.46	2.75E-05
sto-1	65.35	4.00	2.00	1.55E-03
tag-196	109.28	2.13	1.09	1.16E-03
C08E8.4	85.46	77.29	6.27	8.59E-03
gpx-5	89.07	3.15	1.65	3.45E-03
dohh-1	103.16	4.42	2.15	1.20E-04
ccdc-55	90.77	2.29	1.19	8.73E-03
C18E9.9	91.76	3.92	1.97	8.07E-03
fipr-22	148.84	93.86	6.55	5.83E-03
C39E9.11	149.50	2.11	1.07	1.04E-03
C49F5.7	162.69	4.44	2.15	1.97E-03
C53B4.3	30.10	8.07	3.01	2.37E-03
C54G4.7	32.91	8.86	3.15	1.42E-03
ttr-44	173.24	3.59	1.84	1.73E-04
D1086.1	75.08	4.38	2.13	5.43E-03
D1086.3	249.45	7.32	2.87	2.05E-08
D1086.6	64.77	2.75	1.46	9.80E-03
E01G4.3	95.12	2.66	1.41	9.85E-03

F07H5.13	76.53	5.68	2.51	5.26E-03
F08B12.4	738.82	2.88	1.52	4.51E-08
F08G2.5	224.95	18.78	4.23	2.68E-07
oac-14	63.52	4.92	2.30	9.66E-05
F15G9.1	111.51	3.94	1.98	2.29E-04
cut-3	62.27	3.05	1.61	6.31E-03
thn-2	308.74	12.07	3.59	6.33E-10
sac-1	137.05	2.03	1.02	1.33E-03
F32D8.14	83.50	2.60	1.38	6.88E-03
clec-62	66.08	3.20	1.68	6.17E-03
wdr-48	73.62	2.03	1.02	2.44E-03
F36F2.2	56.98	13.92	3.80	2.86E-03
F53B2.8	98.02	3.41	1.77	2.50E-03
F55B11.3	133.13	2.26	1.18	2.19E-03
F56H9.2	1214.36	2.80	1.48	2.66E-08
F57C2.4	221.86	3.52	1.81	5.95E-03
F57F5.1	1173.52	2.10	1.07	5.97E-11
F57G12.1	73.03	3.41	1.77	7.16E-05
ttr-50	278.71	2.16	1.11	8.78E-05
F58D5.5	41.89	2.45	1.29	4.48E-03
amph-1	41.59	2.94	1.55	3.27E-03
dgat-2	83.66	8.09	3.02	1.07E-04
cyp-31A2	107.62	2.12	1.08	3.50E-03
ttr-51	1695.72	3.89	1.96	1.11E-16
cdr-4	385.19	35.55	5.15	2.46E-10
cpg-7	103.01	2.64	1.40	6.55E-03
M01G12.9	23.52	11.87	3.57	5.24E-03
M28.10	200.89	2.68	1.42	4.15E-05
ugt-62	265.77	8.33	3.06	1.02E-12
clec-190	214.72	9.25	3.21	4.81E-07
R11A8.1	28.91	4.38	2.13	6.01E-03
T01D3.6	194.60	10.05	3.33	0
sqst-1	172.63	2.73	1.45	9.14E-08
T13F3.6	106.50	3.89	1.96	2.19E-03
sptl-3	55.77	2.17	1.12	7.72E-03
T23F11.6	95.34	4.82	2.27	4.87E-03
T27D12.1	36.83	2.96	1.57	6.97E-03
T28B8.1	129.52	2.15	1.10	3.90E-03
W01F3.2	196.28	2.81	1.49	5.22E-05
W02D9.6	188.79	3.43	1.78	3.35E-03

W04A8.4	88.11	2.44	1.29	9.20E-03
Y47H10A.5	640.97	38.59	5.27	0
efhd-1	18.10	5.12	2.36	8.95E-03
ttr-25	160.40	3.21	1.68	1.19E-03
ttr-26	147.78	5.84	2.55	1.57E-04
Y54E5A.5	292.34	3.13	1.65	2.13E-05
Y57G11B.5	470.41	3.02	1.59	1.08E-08
ebp-1	70.65	5.67	2.50	8.60E-04
fbxa-114	38.82	6.48	2.70	8.25E-03
MTCE.7	254.84	2.03	1.02	3.44E-03
faah-2	49.55	2.38	1.25	7.25E-03
valv-1	154.47	6.44	2.69	9.84E-04
C06G3.6	32.30	3.45	1.79	6.15E-03
fbxa-163	97.23	113.13	6.82	3.82E-03
catp-3	49.59	3.54	1.82	5.70E-06
C17H12.8	688.36	2.57	1.36	8.74E-10
clec-266	273.13	2.24	1.16	6.76E-05
C25E10.8	119.05	5.12	2.36	4.75E-03
tdo-2	81.20	4.25	2.09	2.29E-05
C33G8.2	38.36	14.25	3.83	3.90E-03
C34H4.2	195.47	7.98	3.00	2.84E-12
C42D4.1	2013.74	4.30	2.10	0
C50F7.5	1486.22	11.51	3.53	0
C53B7.3	152.34	3.20	1.68	1.63E-04
cebp-1	72.37	5.84	2.55	1.84E-04
E04F6.8	302.29	2.68	1.42	6.45E-04
E04F6.9	409.62	6.29	2.65	2.53E-07
numr-2	422.23	339.90	8.41	3.63E-04
numr-1	397.91	321.53	8.33	4.22E-04
F09E5.7	69.92	2.32	1.21	5.76E-03
upb-1	297.27	3.52	1.81	1.00E-10
jmjd-3.1	11.45	6.48	2.70	8.30E-03
F20A1.10	207.25	7.39	2.89	1.18E-04
F21C10.10	323.72	7.97	3.00	1.72E-11
F22H10.2	484.39	42.74	5.42	2.28E-05
F22H10.3	896.88	4.09	2.03	1.10E-09
dct-6	25.48	2.66	1.41	7.71E-03
F29B9.8	53.47	2.70	1.43	2.68E-03
F37C4.5	688.76	3.31	1.73	0
sand-1	144.64	2.24	1.17	3.21E-04

msra-1	120.99	3.97	1.99	5.64E-04
F46H5.7	73.94	2.31	1.21	1.41E-04
F53A9.1	355.42	109.63	6.78	4.36E-03
F53B1.8	13.32	6.48	2.70	9.98E-03
mff-2	94.83	3.75	1.91	3.02E-03
eme-1	54.93	2.51	1.33	8.57E-03
K11H12.8	138.68	2.47	1.30	6.11E-04
asns-2	81.11	10.78	3.43	4.43E-07
R02F2.1	719.39	2.95	1.56	0
cyp-33C8	44.55	6.10	2.61	4.51E-04
T12B3.3	174.47	2.73	1.45	1.54E-05
hex-1	73.91	2.18	1.12	3.00E-03
pqbp-1.1	87.35	2.77	1.47	2.54E-03
T22B7.7	95.35	14.37	3.85	4.19E-06
T22B11.4	13.00	11.87	3.57	5.32E-03
T23B3.2	169.51	5.97	2.58	1.55E-04
T23F2.3	186.68	4.38	2.13	7.98E-03
T24C4.4	150.61	9.20	3.20	3.29E-04
T28A11.2	187.58	3.62	1.86	2.79E-04
W01A11.1	141.83	2.25	1.17	1.22E-03
W05H9.1	282.83	2.20	1.14	7.58E-06
W05H9.3	601.46	4.45	2.15	0
W09C3.7	43.63	6.09	2.61	9.70E-03
Y22D7AL.15	113.51	9.26	3.21	1.37E-03
spp-23	295.02	17.59	4.14	2.65E-05
Y39G10AR.11	45.39	3.76	1.91	3.29E-04
Y58A7A.3	77.10	10.51	3.39	4.88E-09
Y58A7A.5	121.40	19.25	4.27	4.38E-05
acp-6	1031.37	3.55	1.83	0
Y97E10AR.6	116.29	2.15	1.11	2.00E-03
Y102A11A.3	16.49	6.96	2.80	1.60E-04
ZK1055.7	252.49	29.65	4.89	2.12E-14
ZK1290.5	41.86	12.56	3.65	4.23E-03
D1086.11	115.01	2.12	1.08	4.41E-03
T26H5.9	217.15	4.34	2.12	8.39E-04
C18H7.11	34.58	6.88	2.78	5.39E-03
ttr-45	1086.61	6.13	2.62	0
C08A9.10	219.87	3.45	1.79	2.32E-04
rpr-1	397.78	26.37	4.72	2.35E-04
C25F9.11	112.77	17.34	4.12	1.13E-03

Y37H2A.14	279.05	29.92	4.90	4.07E-07
F15B9.10	118.87	5.20	2.38	3.82E-06
F33H12.7	726.36	64.85	6.02	4.17E-10
slrp-1	226.06	2.17	1.11	1.81E-03
paxt-1	108.83	2.19	1.13	6.26E-03
zip-9	111.36	2.49	1.31	7.89E-03
msa-1	193.72	3.77	1.91	4.76E-04
pgph-2	159.22	3.83	1.94	5.69E-05
ipla-2	18.74	3.90	1.96	4.72E-03
nog-1	371.37	4.18	2.06	0
ifo-1	18.33	4.97	2.31	6.94E-04
pgph-3	77.77	8.19	3.03	2.19E-04
ipla-7	79.59	11.09	3.47	1.37E-07

Supplementary Table2

RNA sequencing analysis of animals cultivated at 25°C for 20 hours under LMB exposure

(logFC<-1 p<0.01)

Name	Max group means	Fold change	Log fold change	P-value
clu-1	55.37	-3.16	-1.66	1.24E-07
col-3	800.44	-6.31	-2.66	2.16E-13
col-10	341.04	-12.54	-3.65	1.55E-09
col-13	71.83	-20.50	-4.36	9.64E-04
col-17	220.96	-265.43	-8.05	9.87E-04
col-39	78.28	-9.88	-3.30	9.77E-05
col-41	81.89	-123.84	-6.95	3.88E-03
col-73	263.80	-282.03	-8.14	6.37E-04
col-90	76.07	-95.08	-6.57	6.79E-03
col-93	134.43	-4.58	-2.19	4.30E-04
col-94	105.84	-9.78	-3.29	1.61E-04
col-107	138.25	-12.11	-3.60	9.28E-06
col-125	285.77	-11.70	-3.55	5.00E-08
col-144	511.77	-24.35	-4.61	9.44E-15
col-159	59.35	-18.49	-4.21	1.49E-03
col-166	290.44	-19.98	-4.32	8.88E-11
col-167	304.57	-10.77	-3.43	7.32E-09
cyn-8	27.45	-4.95	-2.31	2.62E-03
dpy-4	283.60	-312.06	-8.29	5.87E-04
dpy-5	218.95	-215.98	-7.75	1.37E-03
dpy-13	258.93	-280.23	-8.13	6.90E-04
dpy-21	16.49	-2.44	-1.29	3.80E-03
elo-2	195.44	-3.24	-1.70	8.71E-07
gfi-1	66.38	-4.80	-2.26	2.90E-07
grl-16	56.70	-8.83	-3.14	2.47E-05
gst-13	60.76	-4.46	-2.16	5.09E-03
hil-5	315.63	-2.16	-1.11	2.09E-04
hsp-1	1916.76	-2.13	-1.09	4.91E-10
hsp-60	262.16	-2.54	-1.35	1.90E-07
imb-1	61.03	-2.35	-1.23	1.25E-04
iars-2	33.52	-2.91	-1.54	6.06E-04
laf-1	52.50	-2.34	-1.22	1.35E-04
lec-9	251.95	-2.08	-1.05	2.82E-03
lys-2	268.85	-2.10	-1.07	9.61E-05

lys-7	564.43	-4.91	-2.30	2.10E-14
mcm-3	228.55	-2.13	-1.09	4.99E-07
mes-1	10.86	-6.43	-2.68	8.01E-03
pqn-27	49.11	-2.59	-1.37	1.17E-03
ram-2	150.52	-3.85	-1.95	5.45E-05
rhr-1	72.07	-3.35	-1.75	1.31E-04
rol-8	67.59	-84.22	-6.40	8.05E-03
rps-2	1144.41	-2.34	-1.23	4.05E-10
sft-4	130.02	-2.20	-1.13	1.64E-03
sqt-2	92.78	-104.73	-6.71	5.42E-03
sur-5	42.56	-2.82	-1.49	2.15E-03
tfg-1	116.48	-2.34	-1.23	1.68E-04
vit-1	430.79	-2.41	-1.27	8.99E-14
vit-4	2141.96	-3.27	-1.71	0
vit-5	3753.82	-2.13	-1.09	6.25E-11
B0024.4	48.39	-5.67	-2.50	1.14E-03
B0035.13	49.16	-4.03	-2.01	4.50E-03
pho-11	126.33	-5.11	-2.35	2.57E-07
ugt-22	23.96	-4.70	-2.23	4.49E-03
cbl-1	26.65	-7.32	-2.87	5.61E-03
C29F3.7	31.73	-6.72	-2.75	1.79E-03
C31C9.2	145.08	-2.08	-1.06	1.25E-03
C32H11.4	50.80	-10.45	-3.39	8.28E-04
dod-24	118.62	-42.72	-5.42	1.54E-05
C47E12.3	40.37	-2.69	-1.43	3.85E-03
C49C3.4	29.25	-4.23	-2.08	5.62E-05
sams-1	60.87	-2.19	-1.13	9.60E-03
din-1	10.92	-2.32	-1.22	4.53E-03
F09B12.3	48.77	-5.76	-2.53	2.02E-04
F11A5.9	37.70	-2.80	-1.49	5.48E-03
F17H10.1	22.50	-7.32	-2.87	4.21E-03
clec-65	194.18	-4.76	-2.25	8.75E-10
tatn-1	44.43	-6.00	-2.59	1.58E-04
F52B5.3	21.43	-2.16	-1.11	8.61E-03
F53F1.4	286.29	-5.50	-2.46	1.41E-05
cut-2	270.41	-2.73	-1.45	5.41E-05
atad-3	55.69	-2.31	-1.21	4.28E-03
F55G11.4	156.99	-9.41	-3.23	2.30E-08

cysl-2	62.92	-3.04	-1.60	5.26E-03
LLC1.2	136.77	-4.16	-2.06	5.67E-06
R04D3.3	144.61	-2.07	-1.05	2.69E-04
gpx-2	80.14	-3.21	-1.68	8.32E-04
R10H10.3	81.19	-2.43	-1.28	8.15E-03
ucr-2.2	50.87	-2.89	-1.53	7.83E-03
mics-1	91.98	-2.25	-1.17	2.81E-03
alh-13	25.48	-11.44	-3.52	8.78E-04
T24B8.5	453.07	-9.31	-3.22	1.87E-09
T25C12.3	40.21	-4.04	-2.02	1.15E-09
flh-3	46.42	-3.22	-1.69	3.41E-03
Y16B4A.2	8.89	-5.48	-2.46	2.29E-03
Y18D10A.11	235.29	-2.59	-1.37	5.37E-06
mrpl-22	45.91	-4.84	-2.27	9.16E-03
dhhc-8	35.11	-2.97	-1.57	7.32E-03
Y40H7A.10	70.62	-7.38	-2.88	1.08E-04
Y53F4B.9	31.15	-4.52	-2.18	6.98E-05
Y54G11A.3	65.44	-2.51	-1.33	1.35E-03
Y105E8A.14	43.17	-3.68	-1.88	4.70E-03
Y106G6H.6	94.77	-2.15	-1.10	1.70E-03
clec-186	56.85	-2.70	-1.43	9.30E-03
ZK1067.3	56.77	-2.91	-1.54	6.09E-03
trpp-3	89.66	-4.22	-2.08	4.73E-03
ZK1307.1	67.31	-5.21	-2.38	5.56E-04
C05D12.3	15.04	-3.73	-1.90	3.76E-03
C01B10.6	78.83	-5.47	-2.45	9.37E-07
hpo-9	59.87	-2.11	-1.07	6.59E-03
sams-3	60.54	-3.43	-1.78	2.60E-04
C07D8.6	276.23	-2.05	-1.04	3.00E-05
mlt-10	23.65	-5.19	-2.38	1.77E-03
C17E7.4	114.08	-2.44	-1.29	1.37E-03
C17H11.6	13.89	-4.22	-2.08	9.57E-03
dyci-1	83.60	-2.10	-1.07	1.09E-03
C23H5.8	45.39	-14.19	-3.83	2.73E-03
C26B9.5	29.30	-6.10	-2.61	3.07E-03
got-2.1	55.49	-3.01	-1.59	3.39E-03
acdh-1	121.67	-3.51	-1.81	1.70E-04
F10E7.5	177.49	-2.15	-1.10	3.46E-03

pud-2.1	1022.42	-2.43	-1.28	1.03E-05
pud-4	289.55	-4.20	-2.07	1.41E-05
pud-1.1	290.11	-144.30	-7.17	3.66E-03
pud-2.2	880.26	-2.50	-1.32	1.58E-07
pud-3	422.34	-3.65	-1.87	4.84E-07
ilys-5	240.30	-2.07	-1.05	3.70E-05
asp-13	35.89	-14.19	-3.83	2.77E-03
F28B4.3	10.84	-2.65	-1.41	8.57E-03
phf-10	22.51	-4.46	-2.16	5.09E-03
chtl-1	22.56	-4.06	-2.02	2.41E-03
F41F3.3	512.22	-2.89	-1.53	5.42E-06
lin-42	11.05	-6.88	-2.78	7.29E-03
F53A9.8	185.70	-2.76	-1.47	9.27E-03
F53B1.4	29.43	-5.15	-2.37	9.65E-03
F56A4.2	426.62	-14.83	-3.89	9.99E-16
F57F4.4	86.41	-3.23	-1.69	6.36E-09
K10C2.1	12.40	-26.55	-4.73	2.02E-04
asp-14	134.62	-47.64	-5.57	2.27E-05
R08E3.1	22.84	-5.36	-2.42	1.28E-06
R08E5.3	48.15	-5.52	-2.46	1.84E-04
hacd-1	131.39	-2.24	-1.16	1.60E-03
R09F10.8	97.07	-3.22	-1.69	3.83E-04
cra-1	61.32	-2.51	-1.33	3.17E-05
T10F2.2	58.70	-2.88	-1.52	6.07E-03
W10C8.5	104.01	-2.54	-1.34	1.14E-03
Y4C6B.1	69.32	-2.27	-1.18	3.33E-03
clec-209	425.26	-12.39	-3.63	3.33E-16
pud-1.2	1360.62	-2.07	-1.05	1.15E-04
Y34B4A.6	244.49	-2.05	-1.03	5.72E-04
Y34B4A.9	131.57	-2.91	-1.54	7.18E-04
Y47D7A.13	134.66	-7.89	-2.98	1.50E-05
Y47G6A.18	44.74	-3.22	-1.69	5.54E-03
Y50D4C.5	29.60	-3.08	-1.62	9.33E-03
Y51F10.7	147.30	-13.97	-3.80	4.39E-06
Y54F10AM.8	38.22	-2.91	-1.54	6.24E-03
iffb-1	68.99	-2.53	-1.34	2.63E-05
clec-85	125.60	-4.75	-2.25	9.24E-06
mrps-25	82.43	-4.03	-2.01	4.46E-03

cct-8	194.77	-2.33	-1.22	5.08E-08
ZK180.5	104.70	-2.62	-1.39	3.74E-04
F52E1.14	379.27	-2.30	-1.20	1.25E-05
ctsa-2	33.49	-16.51	-4.05	1.87E-03
acox-1.4	15.59	-10.33	-3.37	9.19E-03
myrf-1	13.60	-4.70	-2.23	6.66E-03
meg-4	24.15	-3.65	-1.87	2.24E-03
hphd-1	42.71	-3.12	-1.64	7.34E-03
vrp-1	80.81	-3.09	-1.63	5.11E-03
oxa-1	44.99	-2.80	-1.49	5.94E-03
slc-25A46	41.86	-2.76	-1.46	7.97E-03
endu-2	44.90	-2.47	-1.30	5.75E-03
pola-1	56.12	-2.14	-1.10	4.33E-05
bckd-1B	100.16	-2.07	-1.05	3.54E-03

Supplementary Table3

RNA sequencing analysis of animals exposed to LMB at 2°C for 3 hr.

(LogFC>1, p<0.01)

Name	Max group mean	Log_2 fold change	Fold change	P-value
abf-2	55.07	2.53	5.78	6.93.E-10
abf-6	14.89	1.24	2.36	2.30.E-03
abhd-14	15.48	1.17	2.25	1.80.E-03
abt-4	24.06	1.45	2.74	4.74.E-11
abu-8	21.39	1.30	2.46	6.06.E-03
acbp-6	10.94	1.31	2.48	3.27.E-03
acc-1	1.54	2.01	4.03	8.48.E-04
acd-2	4.93	1.04	2.06	5.33.E-03
ace-3	6.28	1.37	2.58	3.70.E-04
acox-1.3	7.48	1.20	2.30	9.49.E-04
acp-6	830.60	1.44	2.72	3.16.E-13
acr-15	3.83	1.37	2.58	2.34.E-03
acr-18	3.50	1.64	3.12	3.24.E-04
acs-2	89.27	2.06	4.18	4.11.E-07
akt-2	17.95	1.12	2.18	6.85.E-07
alh-5	11.95	1.07	2.10	6.01.E-04
amt-1	32.14	2.31	4.95	8.29.E-19
anr-36	2.77	2.05	4.13	2.97.E-03
anr-45	1.05	2.26	4.79	5.49.E-03
aqp-6	3.54	1.40	2.65	4.10.E-03
arl-13	3.44	1.74	3.34	5.99.E-04
arrd-11	3.24	3.58	11.98	6.05.E-06
arrd-8	12.00	3.81	14.05	6.63.E-10
arrd-9	5.45	2.40	5.30	5.05.E-05
asah-2	74.97	1.38	2.59	3.40.E-13
asm-2	11.19	1.73	3.31	1.65.E-07
asns-2	48.33	1.79	3.45	1.79.E-15
asp-1	2558.93	1.52	2.86	4.03.E-10
asp-12	22.57	1.98	3.94	3.45.E-11
asp-6	2017.06	1.13	2.19	1.65.E-07
asp-9	5.32	5.30	39.40	9.55.E-09
B0205.14	15.41	2.20	4.61	1.71.E-03
B0207.2	3.15	2.06	4.18	9.74.E-04
B0244.5	1.95	2.49	5.61	2.07.E-04
B0281.3	47.36	2.21	4.63	1.03.E-14
B0310.3	2.02	1.73	3.32	6.62.E-03
B0348.1	9.18	5.19	36.39	5.85.E-04
B0361.4	2.59	1.64	3.11	4.77.E-03
B0403.3	18.63	1.74	3.33	1.40.E-08
B0454.5	39.84	1.16	2.24	5.40.E-05
B0457.6	37.04	1.16	2.24	1.51.E-04
B0462.5	9.33	2.04	4.12	7.96.E-06
B0507.8	3.85	1.85	3.60	1.79.E-03
B0511.11	3.68	1.45	2.72	1.74.E-03
bath-45	3.38	2.27	4.81	1.90.E-04
bath-46	5.64	1.43	2.70	5.28.E-03
bath-47	14.30	2.38	5.19	1.07.E-13

bcmo-2	15.00	1.63	3.09	1.76.E-10
BE0003N10.6	4.47	1.56	2.94	1.45.E-03
best-21	20.20	1.87	3.66	1.46.E-08
best-8	3.16	1.22	2.33	4.38.E-03
bro-1	3.70	1.73	3.31	4.84.E-03
btb-16	16.11	1.33	2.52	8.24.E-05
btb-9	3.75	1.30	2.46	8.58.E-03
C01B4.7	3.88	1.43	2.70	1.35.E-03
C01G10.17	11.23	3.28	9.71	6.09.E-05
C01G10.4	16.36	4.58	23.87	2.55.E-06
C01G10.5	22.05	4.47	22.21	4.65.E-08
C03A7.2	1.27	2.65	6.27	6.38.E-03
C04C11.23	4.41	6.92	121.03	5.57.E-03
C04C3.14	41.41	7.15	141.86	3.87.E-08
C04F12.5	76.41	1.68	3.21	1.77.E-10
C04G6.5	15.55	2.00	4.00	9.85.E-05
C05D9.9	16.69	1.01	2.01	4.91.E-03
C06E4.3	2.14	1.57	2.98	5.87.E-03
C07A9.9	65.06	1.96	3.90	3.80.E-12
C07E3.3	23.53	2.63	6.20	1.71.E-15
C07F11.2	2.48	1.53	2.89	1.32.E-03
C08B6.4	6.77	1.79	3.47	9.67.E-07
C08E8.10	25.81	2.71	6.55	4.22.E-11
C08E8.4	108.41	3.13	8.76	1.85.E-41
C08G5.7	8.51	1.13	2.19	4.45.E-03
C08G9.1	23.19	1.30	2.45	2.60.E-04
C08H9.15	34.42	1.04	2.06	6.16.E-04
C09H10.9	0.95	2.93	7.62	2.89.E-03
C10C5.2	13.16	1.85	3.61	1.24.E-07
C10C5.3	15.96	1.43	2.70	3.17.E-06
C10F3.7	10.69	1.85	3.59	5.50.E-04
C11E4.7	11.97	1.81	3.50	2.24.E-06
C13F10.1	4.72	1.49	2.80	5.33.E-03
C13G3.1	39.66	2.27	4.82	4.41.E-12
C14A11.2	2.04	2.71	6.55	9.92.E-04
C14A4.13	1.89	2.16	4.47	7.25.E-04
C15F1.5	5.54	1.12	2.18	2.54.E-03
C15H11.16	8.41	4.12	17.43	7.01.E-08
C16C10.13	1.63	2.43	5.38	9.82.E-03
C16C8.14	23.05	1.31	2.48	1.31.E-05
C16D9.4	11.75	1.23	2.34	2.16.E-04
C17C3.3	2.97	4.09	16.98	1.73.E-06
C17C3.5	3.92	3.45	10.94	3.60.E-03
C17H12.8	355.88	1.05	2.07	3.57.E-07
C18A11.1	34.26	1.58	2.98	1.36.E-07
C18E9.9	104.89	1.37	2.58	1.63.E-08
C18H7.1	5.63	2.43	5.40	2.09.E-07
C18H7.11	47.80	3.49	11.22	2.33.E-25
C23G10.1	2.10	2.37	5.17	6.54.E-04
C23H5.15	15.70	3.27	9.65	5.05.E-08
C25D7.5	12.82	1.96	3.88	3.14.E-08

C25E10.8	121.01	2.70	6.48	1.22.E-19
C25F9.11	129.59	3.73	13.26	8.00.E-37
C25F9.12	17.15	2.63	6.19	2.06.E-08
C25F9.16	18.64	5.25	38.11	7.98.E-09
C25F9.2	0.21	3.95	15.46	6.34.E-03
C25F9.5	9.22	1.84	3.57	7.70.E-08
C25F9.6	4.55	2.26	4.80	7.80.E-04
C26D10.3	6.24	1.00	2.00	4.98.E-03
C26E1.2	13.13	2.48	5.57	2.51.E-12
C27A2.8	3.21	2.03	4.09	7.37.E-03
C27H5.2	16.78	1.78	3.43	3.22.E-10
C28G1.2	3.73	1.98	3.96	5.05.E-04
C29F9.2	13.66	1.03	2.04	4.77.E-03
C29G2.3	3.25	1.93	3.81	2.39.E-03
C29H12.6	6.54	1.13	2.20	3.57.E-03
C30H6.12	15.76	4.91	30.00	6.72.E-13
C32D5.6	8.70	1.02	2.03	2.02.E-03
C32F10.4	87.58	1.20	2.29	3.52.E-10
C33C12.4	9.63	3.98	15.74	2.09.E-08
C33D9.3	10.47	1.06	2.08	4.32.E-03
C33H5.13	75.95	1.74	3.35	1.34.E-11
C34B7.1	6.67	1.61	3.05	6.70.E-05
C34F11.8	23.56	1.30	2.46	1.52.E-04
C34H4.2	138.45	2.18	4.52	6.22.E-34
C35A5.11	22.60	1.65	3.14	6.59.E-05
C36C9.5	0.81	2.55	5.86	8.24.E-03
C36E8.4	11.55	1.53	2.89	4.03.E-06
C37A5.3	17.39	2.74	6.69	2.17.E-06
C37C3.10	10.17	3.34	10.16	1.12.E-07
C37C3.12	7.78	1.39	2.62	4.35.E-03
C38C3.4	9.03	1.21	2.32	4.76.E-04
C38H2.3	16.40	1.35	2.54	1.66.E-04
C39B10.7	2.99	2.36	5.12	2.38.E-03
C39D10.11	15.82	1.73	3.32	3.24.E-08
C39F7.5	7.55	1.14	2.21	8.65.E-05
C41G7.8	17.18	1.57	2.96	1.47.E-04
C42D4.1	1475.69	2.25	4.77	3.82.E-24
C42D4.19	7.66	2.89	7.40	7.38.E-05
C42D4.3	56.96	1.48	2.79	5.53.E-03
C42D8.1	21.75	1.33	2.51	1.63.E-04
C44H9.4	2.54	1.30	2.46	1.94.E-03
C45B11.2	7.02	2.23	4.70	3.15.E-05
C45B2.1	60.22	1.11	2.17	6.67.E-05
C45B2.2	122.39	1.63	3.10	1.51.E-04
C45E5.4	19.38	2.06	4.18	9.36.E-06
C45G7.13	4.50	1.21	2.31	4.11.E-03
C46A5.4	3.26	2.42	5.37	1.36.E-08
C46F2.1	7.69	1.14	2.21	4.98.E-03
C46G7.5	12.65	1.22	2.33	5.87.E-03
C46H11.10	2.71	1.50	2.83	8.96.E-03
C47E12.12	2.73	2.16	4.47	5.94.E-03

C48B6.9	31.13	1.63	3.10	1.73.E-08
C49A9.3	4.77	1.07	2.10	5.05.E-03
C49A9.6	15.94	1.35	2.55	1.65.E-04
C49A9.9	24.76	1.35	2.55	1.22.E-06
C49C3.11	7.04	5.16	35.74	5.72.E-09
C49C8.1	1.12	2.55	5.85	1.74.E-03
C49C8.2	6.52	1.46	2.75	5.73.E-03
C49F8.1	2.75	1.53	2.88	6.96.E-03
C49G7.12	28.28	3.21	9.25	1.13.E-17
C50A2.3	14.74	1.75	3.37	2.88.E-06
C50B8.4	8.32	2.53	5.77	8.45.E-06
C50E3.6	7.15	2.03	4.07	1.70.E-05
C50F7.5	1374.43	3.81	14.06	2.13.E-25
C53B4.3	31.75	2.18	4.54	3.83.E-18
C53B7.3	138.64	1.64	3.12	4.18.E-15
C54C6.7	26.19	2.44	5.43	6.06.E-08
C54C8.12	124.25	4.07	16.79	4.10.E-15
C54F6.17	8.27	5.30	39.47	3.23.E-04
C54F6.18	4.20	3.10	8.57	3.91.E-03
C54G6.2	1.26	2.38	5.21	1.47.E-03
C55A1.6	19.01	4.16	17.88	3.24.E-09
C55C3.3	3.83	1.30	2.46	7.67.E-04
C56G7.3	16.13	1.20	2.30	7.35.E-03
cah-1	8.72	1.76	3.38	4.02.E-04
catp-3	62.39	2.43	5.39	3.78.E-27
cav-2	9.70	1.95	3.86	1.25.E-05
ccep-290	1.27	1.35	2.54	2.64.E-03
ccg-1	301.96	1.17	2.25	1.34.E-08
cdd-1	33.40	3.46	10.98	5.01.E-23
cdr-4	188.32	3.06	8.36	7.97.E-56
ceh-62	7.04	2.01	4.02	3.31.E-06
ceh-79	5.91	1.15	2.22	1.48.E-03
cex-1	41.35	1.88	3.69	2.52.E-08
cex-2	17.11	1.15	2.22	2.52.E-03
cfap-36	5.17	2.05	4.13	9.56.E-07
che-3	0.71	1.33	2.52	1.35.E-03
chil-18	5.01	4.53	23.14	4.10.E-07
cima-1	7.03	1.09	2.13	9.22.E-04
ckr-2	3.05	1.43	2.70	3.97.E-03
clc-1	178.90	1.32	2.50	4.15.E-12
cld-9	25.55	1.61	3.06	8.09.E-09
clec-11	1.27	3.84	14.35	8.42.E-04
clec-121	1.01	6.40	84.16	9.29.E-03
clec-125	4.27	4.52	22.91	2.48.E-12
clec-142	5.64	4.14	17.59	2.30.E-10
clec-143	1.75	2.50	5.66	4.22.E-04
clec-161	0.36	3.92	15.18	5.92.E-03
clec-169	1.52	2.02	4.04	1.81.E-03
clec-190	122.82	2.59	6.03	2.02.E-12
clec-221	14.40	3.17	9.02	1.30.E-08
clec-233	7.11	3.33	10.06	5.80.E-08

clec-47	28.32	1.43	2.70	2.53.E-05
clec-5	65.31	2.39	5.26	9.27.E-24
clec-52	17.26	1.32	2.50	1.38.E-03
clec-6	7.33	4.52	23.01	2.75.E-13
clec-60	21.00	5.12	34.79	3.32.E-27
clec-70	1.14	3.37	10.30	4.47.E-04
clik-2	44.18	1.06	2.08	1.09.E-06
clik-3	70.85	1.34	2.52	5.25.E-08
clp-4	38.34	1.06	2.09	1.07.E-07
clp-9	2.26	1.56	2.95	2.47.E-03
clx-1	1.96	3.80	13.90	1.74.E-06
cnc-11	6.47	2.19	4.56	6.55.E-03
col-106	1679.26	1.25	2.38	1.40.E-10
col-108	2.91	7.23	150.07	3.05.E-03
col-113	15.28	1.62	3.08	1.17.E-03
col-121	17.01	1.11	2.16	5.06.E-04
col-124	715.49	1.33	2.52	3.66.E-05
col-129	59.75	2.79	6.92	2.87.E-06
col-132	1.59	3.21	9.23	9.44.E-04
col-139	217.12	4.16	17.84	4.61.E-29
col-140	2313.68	1.63	3.10	2.77.E-12
col-141	15.04	3.36	10.26	3.42.E-07
col-142	120.81	2.53	5.76	2.53.E-13
col-165	105.11	1.84	3.58	8.60.E-17
col-178	1154.77	1.41	2.65	9.86.E-12
col-181	2075.50	1.31	2.48	4.31.E-11
col-183	4.61	4.40	21.10	4.61.E-07
col-184	1336.00	1.22	2.33	1.07.E-10
col-19	419.53	1.38	2.60	5.56.E-09
col-45	2.25	3.89	14.82	2.18.E-05
col-50	9.12	9.40	674.24	6.97.E-05
col-62	3.85	2.79	6.89	2.56.E-05
col-74	79.64	1.79	3.46	7.70.E-11
col-81	28.68	1.97	3.91	1.52.E-04
col-84	7.76	5.71	52.45	5.94.E-11
col-85	3.14	4.76	27.13	9.53.E-06
col-95	1585.03	1.20	2.30	6.13.E-09
col-96	124.50	4.52	22.87	2.33.E-33
comt-3	29.93	1.31	2.48	9.14.E-06
cpg-7	177.56	2.17	4.50	2.05.E-17
cpn-3	615.17	1.03	2.04	4.01.E-08
cpn-4	139.64	1.31	2.47	3.51.E-10
cpna-1	27.58	1.07	2.10	7.10.E-09
cpr-1	223.90	2.01	4.01	8.64.E-14
cpr-3	398.03	3.54	11.62	1.89.E-69
cpr-4	2055.24	4.15	17.73	1.38.E-80
ctl-1	86.28	1.10	2.15	1.85.E-08
cul-6	5.74	1.21	2.31	5.46.E-04
cut-3	74.77	2.15	4.44	2.88.E-16
cut-5	4.09	1.93	3.81	1.13.E-04
cutl-13	2.83	1.39	2.62	6.11.E-03

cutl-15	7.08	1.20	2.29	1.69.E-03
cutl-23	7.30	1.58	3.00	3.57.E-06
cyp-13A1	1.90	1.76	3.39	5.59.E-03
cyp-13A6	5.52	3.60	12.10	2.21.E-11
cyp-13A7	2.24	2.08	4.23	1.07.E-03
cyp-13A8	6.68	4.56	23.60	7.73.E-13
cyp-13B1	2.98	1.77	3.41	1.00.E-03
cyp-14A4	3.37	3.91	14.99	3.88.E-08
cyp-25A3	10.86	1.45	2.74	1.10.E-05
cyp-33C7	14.51	1.85	3.60	3.49.E-08
cyp-33C8	65.38	2.70	6.50	1.81.E-23
cyp-33D1	1.67	2.36	5.13	3.13.E-04
cyp-34A10	0.83	2.70	6.52	5.71.E-03
cyp-34A5	2.28	1.57	2.97	3.69.E-03
cyp-34A8	20.36	1.09	2.13	2.10.E-05
cyp-34A9	14.23	1.35	2.55	5.58.E-05
D1044.1	17.82	2.46	5.50	1.28.E-12
D1054.5	19.29	1.57	2.96	3.11.E-06
D1086.1	72.50	1.38	2.60	3.00.E-08
D1086.11	109.23	1.47	2.76	7.71.E-13
D1086.2	14.81	1.68	3.21	8.10.E-05
D1086.3	196.58	1.85	3.62	6.31.E-19
D1086.6	76.00	1.12	2.17	1.34.E-07
D1086.9	0.57	4.25	19.04	3.00.E-03
D2005.6	4.67	1.19	2.28	2.56.E-03
D2045.7	7.60	1.02	2.02	4.70.E-03
D2096.5	7.49	1.80	3.48	1.63.E-03
del-3	2.37	2.62	6.15	8.31.E-05
dgat-2	79.53	2.10	4.28	1.25.E-23
dhc-3	0.42	1.54	2.90	2.66.E-03
dhcr-7	5.24	1.51	2.85	1.15.E-04
dhs-14	86.87	2.21	4.62	1.80.E-22
dhs-26	4.22	4.03	16.39	2.74.E-07
dlhd-1	3.39	1.64	3.11	4.38.E-03
dmd-9	29.61	1.41	2.66	4.94.E-07
dod-20	2.33	3.44	10.85	7.17.E-06
dod-3	134.47	2.34	5.06	8.52.E-12
dos-3	2.69	4.86	29.03	1.10.E-08
doxa-1	18.73	2.43	5.37	6.89.E-08
dpy-1	6.99	1.10	2.14	1.96.E-03
dpy-14	259.65	1.06	2.08	2.04.E-05
dpy-17	310.44	1.17	2.25	3.38.E-07
duox-2	0.30	4.40	21.15	1.21.E-03
dyf-2	2.87	1.61	3.05	5.97.E-05
E02A10.3	3.94	1.27	2.42	3.74.E-03
E03H4.8	13.47	1.44	2.72	7.51.E-06
E04F6.9	612.99	2.42	5.35	4.53.E-28
eak-4	1.46	3.31	9.93	3.86.E-03
egl-1	7.13	1.42	2.67	2.12.E-03
elt-4	2.90	2.87	7.33	3.35.E-03
ensh-1	4.04	1.25	2.37	2.85.E-03

enu-3.6	15.58	1.12	2.17	5.86.E-04
eol-1	15.01	2.85	7.20	6.42.E-09
F01D4.8	1.35	2.59	6.01	1.69.E-03
F02C12.1	17.56	1.34	2.53	7.60.E-09
F07A11.1	1.13	1.79	3.45	5.72.E-03
F07C3.9	11.36	2.51	5.69	7.48.E-05
F07E5.9	0.99	3.27	9.67	1.29.E-03
F08A8.8	3.60	2.06	4.16	4.67.E-04
F08B12.4	1232.94	1.78	3.43	1.14.E-15
F08F1.3	10.40	1.01	2.02	9.18.E-03
F08F8.6	1.95	1.88	3.68	6.71.E-03
F08G2.8	15.59	3.93	15.23	1.09.E-14
F09C12.2	1.79	3.19	9.11	1.37.E-04
F09C6.3	3.83	2.11	4.32	3.30.E-04
F09E5.12	2.36	1.75	3.37	4.38.E-03
F09E5.16	12.96	1.55	2.92	1.75.E-05
F09F9.4	3.05	1.10	2.15	7.53.E-03
F10A3.4	7.14	2.06	4.18	4.11.E-07
F10D2.10	11.16	1.71	3.28	5.23.E-06
F10E9.12	42.16	1.22	2.33	8.78.E-05
F11C7.6	9.71	6.40	84.70	1.10.E-06
F11D11.3	3.03	4.51	22.82	1.85.E-05
F11E6.3	406.94	1.39	2.61	9.29.E-11
F11E6.6	6.05	2.37	5.18	1.40.E-06
F12E12.11	9.40	1.66	3.15	1.41.E-04
F13B12.3	1.80	1.50	2.84	2.86.E-03
F13D11.4	21.12	1.01	2.01	1.41.E-03
F13E9.14	11.53	5.60	48.56	1.14.E-09
F13H10.5	5.74	1.92	3.79	7.98.E-06
F13H6.3	25.27	1.29	2.45	4.10.E-09
F13H8.1	8.60	2.09	4.25	1.28.E-05
F14B8.4	18.04	1.16	2.24	7.30.E-03
F14D7.10	6.27	1.83	3.56	3.03.E-03
F14D7.5	20.58	8.47	354.42	3.60.E-04
F14F9.3	1.07	3.75	13.41	6.85.E-05
F14H3.12	24.70	1.29	2.45	7.66.E-05
F15B9.6	25.24	4.42	21.43	1.57.E-33
F15E6.6	5.16	1.08	2.11	1.96.E-04
F15G9.1	125.84	1.79	3.45	1.72.E-17
F15G9.5	6.75	1.14	2.21	8.33.E-03
F16B3.3	7.25	1.25	2.39	3.14.E-03
F16C3.1	3.63	1.22	2.33	8.73.E-03
F16H6.10	55.74	2.82	7.06	1.39.E-22
F16H6.9	0.52	4.06	16.66	3.07.E-03
F17C11.22	1.70	2.36	5.13	4.27.E-03
F18G5.6	63.69	1.39	2.62	1.05.E-06
F19B10.14	8.70	1.76	3.39	7.30.E-03
F19B10.4	3.42	2.57	5.94	1.44.E-04
F19B2.5	47.32	1.09	2.12	7.78.E-06
F19B2.6	6.08	2.10	4.27	3.21.E-08
F19C7.2	27.79	3.38	10.43	1.13.E-30

F19F10.3	23.97	1.65	3.14	1.51.E-05
F20A1.10	221.77	1.67	3.19	1.38.E-16
F20B10.3	10.96	1.50	2.83	2.35.E-03
F20D6.5	20.35	3.41	10.62	6.07.E-15
F20G2.5	8.11	1.34	2.54	1.07.E-05
F20H11.4	2.96	2.02	4.05	4.00.E-04
F21C10.10	329.74	2.10	4.28	7.66.E-21
F21C10.3	5.29	1.52	2.87	1.39.E-03
F21D5.4	13.32	1.12	2.17	2.95.E-03
F21G4.1	11.84	1.52	2.87	2.03.E-05
F21G4.5	4.45	1.60	3.03	8.70.E-04
F21H7.2	1.96	1.36	2.57	8.97.E-03
F22H10.10	14.55	7.08	135.27	3.39.E-03
F22H10.2	300.32	4.10	17.20	1.42.E-47
F22H10.3	1301.79	2.36	5.12	1.35.E-35
F23C8.7	1.89	1.76	3.40	3.29.E-03
F23C8.8	3.99	2.27	4.84	7.93.E-05
F23F12.12	50.13	1.54	2.92	4.49.E-11
F25B3.5	6.43	1.74	3.34	1.06.E-04
F25E5.3	1.66	1.86	3.62	6.54.E-03
F25E5.4	2.97	1.21	2.32	7.78.E-03
F25H5.7	0.85	2.68	6.43	3.17.E-03
F26D11.12	12.19	1.49	2.80	1.06.E-03
F26D11.20	11.45	2.07	4.21	7.88.E-04
F28H7.2	2.17	2.66	6.32	8.12.E-04
F29A7.4	3.61	1.73	3.31	6.54.E-03
F31D4.5	12.03	1.96	3.90	1.01.E-07
F32A5.4	76.71	1.17	2.25	5.11.E-08
F32A5.8	37.21	1.88	3.69	9.45.E-11
F32A5.9	29.40	1.69	3.24	2.32.E-06
F32E10.9	18.65	1.53	2.89	6.51.E-06
F32H5.3	11.45	1.38	2.61	1.33.E-03
F33H12.7	580.89	5.35	40.90	3.78.E-120
F35B12.3	31.88	1.10	2.15	2.72.E-04
F35C11.4	9.84	1.37	2.58	1.56.E-04
F35C5.1	3.22	1.62	3.08	9.12.E-03
F35E12.2	7.09	3.50	11.29	3.60.E-13
F35E12.6	85.95	1.33	2.52	2.04.E-09
F35E2.5	3.03	2.89	7.41	8.53.E-08
F35E8.10	7.18	5.63	49.62	2.77.E-07
F35E8.13	3.77	3.24	9.42	1.28.E-05
F35F10.13	10.75	1.02	2.03	6.66.E-03
F35F10.5	8.90	1.05	2.07	7.15.E-03
F36F2.2	69.20	1.81	3.50	2.02.E-11
F36G3.1	25.81	1.27	2.41	2.84.E-08
F36G9.7	4.70	3.21	9.27	2.99.E-05
F36H9.4	28.22	1.22	2.32	1.50.E-03
F37C4.5	775.51	1.78	3.43	6.84.E-19
F37D6.3	3.54	2.07	4.20	7.04.E-03
F39G3.4	4.44	5.70	52.16	3.65.E-05
F40D4.13	12.94	2.71	6.56	4.16.E-04

F40E3.5	7.69	3.94	15.37	1.51.E-09
F40F8.5	139.23	1.04	2.06	1.24.E-06
F40F9.10	4.07	1.57	2.98	8.36.E-04
F40H7.12	28.08	4.07	16.80	4.27.E-15
F41B4.3	27.99	1.05	2.07	1.68.E-03
F41C3.8	9.87	1.58	2.99	1.18.E-04
F41G3.21	10.02	1.82	3.53	2.00.E-03
F42A10.7	19.55	2.04	4.12	1.59.E-04
F42C5.4	4.02	1.84	3.57	3.62.E-05
F43C11.7	18.96	1.33	2.51	3.13.E-05
F43C11.8	1.21	3.02	8.12	2.89.E-03
F43G6.8	16.63	1.00	2.00	2.06.E-03
F44F4.3	3.95	2.17	4.50	6.21.E-04
F45E1.4	55.00	2.39	5.23	1.89.E-20
F45E12.6	3.32	3.44	10.82	1.51.E-03
F45H10.5	2.35	2.40	5.29	5.07.E-03
F46A9.1	3.86	6.12	69.61	7.75.E-06
F46H5.7	104.83	1.21	2.32	7.29.E-10
F47B8.2	43.86	1.49	2.80	1.15.E-06
F47B8.4	11.45	2.09	4.27	5.34.E-07
F47G3.4	52.28	1.68	3.20	1.32.E-06
F47G9.4	4.18	1.23	2.34	1.10.E-03
F48A9.2	4.57	1.46	2.75	4.39.E-04
F48G7.4	7.81	1.02	2.02	5.83.E-03
F49B2.6	1.80	1.35	2.54	6.13.E-03
F49C12.7	21.11	1.07	2.10	4.04.E-05
F49H6.3	8.00	2.60	6.06	7.33.E-05
F49H6.5	3.17	2.56	5.88	1.12.E-04
F52E4.5	51.56	1.70	3.24	9.37.E-07
F53A2.9	9.78	1.49	2.81	9.27.E-05
F53A9.3	2.85	2.52	5.72	5.41.E-03
F53A9.6	177.10	2.07	4.19	3.41.E-08
F53A9.7	117.03	2.34	5.05	1.14.E-08
F53B2.8	172.72	1.26	2.39	2.42.E-05
F53C3.3	3.41	1.48	2.80	3.87.E-03
F53E10.5	0.67	3.87	14.65	5.03.E-03
F53F8.4	48.68	1.44	2.70	4.48.E-07
F54C8.6	6.83	1.28	2.42	2.20.E-03
F54F7.3	77.09	1.44	2.71	1.25.E-07
F54F7.9	12.83	2.50	5.67	4.27.E-07
F54H5.2	2.17	2.85	7.23	4.51.E-05
F55B11.4	189.80	1.59	3.01	3.25.E-17
F55D12.1	6.10	1.42	2.68	7.15.E-03
F55F3.2	18.77	1.50	2.83	4.42.E-07
F55G7.1	0.88	3.33	10.05	2.05.E-03
F56A4.12	3.26	2.20	4.59	2.91.E-05
F56C11.6	11.63	1.06	2.08	2.63.E-03
F56C3.9	18.13	2.51	5.69	7.07.E-10
F56D2.5	6.49	1.56	2.95	8.55.E-06
F56D5.2	1.61	4.89	29.58	3.31.E-04
F56D5.6	33.53	1.03	2.05	1.00.E-05

F56H6.2	2.04	2.59	6.00	1.16.E-03
F56H9.2	1450.72	1.44	2.71	1.61.E-12
F57A8.7	0.72	4.06	16.69	3.37.E-03
F57B9.3	18.59	5.04	32.83	4.74.E-23
F57B9.8	1.04	1.98	3.94	8.47.E-03
F57F5.1	906.02	1.24	2.36	4.21.E-10
F57G4.11	1.66	3.30	9.82	3.82.E-04
F57G8.7	34.21	6.47	88.66	8.59.E-12
F57H12.6	56.53	1.63	3.09	3.71.E-04
F58B4.3	20.68	1.19	2.28	2.11.E-03
F58F9.4	7.30	1.88	3.69	6.66.E-05
F58G1.7	4.86	1.09	2.12	3.84.E-03
F58H7.5	1.94	1.88	3.69	9.22.E-03
F59A6.10	3.70	2.82	7.09	1.66.E-03
F59A7.2	24.93	2.04	4.13	9.69.E-06
F59B1.10	7.55	3.01	8.03	2.31.E-10
F59B1.8	31.32	1.77	3.41	1.06.E-11
F59C6.16	40.25	1.07	2.10	9.70.E-03
famh-161	1.94	1.26	2.40	5.56.E-03
far-3	159.19	2.79	6.94	1.60.E-21
far-7	41.94	2.09	4.27	2.91.E-08
fat-7	10.89	1.21	2.31	5.30.E-04
fbxa-107	39.01	1.05	2.08	2.59.E-06
fbxa-108	28.60	1.49	2.82	2.43.E-08
fbxa-114	32.24	1.12	2.18	7.39.E-06
fbxa-115	9.32	1.40	2.64	2.54.E-04
fbxa-118	1.07	2.34	5.05	9.66.E-03
fbxa-135	3.88	3.01	8.04	1.76.E-08
fbxa-138	3.11	4.53	23.17	3.12.E-05
fbxa-144	1.07	3.27	9.67	3.24.E-03
fbxa-150	11.74	1.68	3.21	1.26.E-06
fbxa-151	3.41	2.12	4.35	1.01.E-03
fbxa-156	42.18	2.23	4.71	7.92.E-15
fbxa-157	15.02	3.49	11.20	1.05.E-12
fbxa-158	8.81	4.07	16.75	5.39.E-13
fbxa-163	70.32	5.98	62.94	1.47.E-58
fbxa-164	5.62	4.63	24.83	6.80.E-10
fbxa-165	2.24	7.32	159.38	2.54.E-03
fbxa-182	12.01	1.70	3.24	1.23.E-08
fbxa-188	3.20	2.72	6.60	3.59.E-05
fbxa-26	4.55	1.80	3.49	7.64.E-04
fbxa-3	3.34	1.52	2.87	1.47.E-03
fbxa-30	7.44	3.47	11.08	2.36.E-10
fbxa-35	1.57	3.25	9.50	5.79.E-04
fbxa-36	1.26	2.93	7.62	2.87.E-03
fbxa-37	7.70	1.45	2.74	5.36.E-04
fbxa-4	4.53	1.61	3.05	4.91.E-04
fbxa-42	1.95	1.72	3.30	6.59.E-03
fbxa-53	1.94	1.78	3.43	9.77.E-03
fbxa-55	5.30	1.85	3.60	3.89.E-05
fbxa-63	5.55	1.57	2.96	1.65.E-03

fbxa-66	3.42	2.14	4.40	4.20.E-04
fbxa-69	2.43	1.56	2.96	3.73.E-03
fbxa-77	5.42	1.15	2.22	8.58.E-03
fbxa-79	12.62	2.02	4.05	2.93.E-07
fbxa-80	5.78	1.82	3.53	5.89.E-05
fbxa-88	4.14	1.95	3.87	1.59.E-04
fbxa-9	1.58	3.35	10.18	6.88.E-04
feh-1	10.07	1.12	2.17	4.92.E-04
fip-6	21.06	1.40	2.65	2.25.E-03
fipr-2	56.07	1.02	2.03	2.59.E-03
fipr-22	96.40	3.90	14.94	2.62.E-24
fipr-23	6.57	2.09	4.26	5.36.E-04
fipr-24	125.95	6.06	66.86	3.78.E-29
fipr-26	16.63	2.73	6.63	1.26.E-04
fkh-8	4.11	1.41	2.66	7.30.E-03
flp-10	29.93	1.06	2.09	8.43.E-04
fmo-1	17.01	1.17	2.25	2.87.E-05
folt-3	1.92	3.44	10.87	1.16.E-05
fpn-1.1	30.58	1.57	2.98	2.00.E-09
frpr-7	2.91	1.36	2.57	2.52.E-03
ftn-1	27.74	2.94	7.69	5.61.E-10
ftr-1	33.24	1.37	2.59	2.70.E-07
fut-2	6.06	2.44	5.44	5.78.E-06
gasr-8	1.44	2.20	4.59	4.43.E-03
gba-2	1.83	2.54	5.80	9.75.E-04
gcy-19	4.29	2.97	7.85	1.40.E-11
gcy-23	1.53	1.73	3.31	4.30.E-04
gcy-31	0.86	1.92	3.80	5.72.E-03
gcy-34	1.15	3.99	15.93	8.39.E-05
gem-4	35.99	1.43	2.69	9.19.E-10
glb-29	2.35	1.47	2.77	4.46.E-03
gln-3	217.25	1.12	2.17	6.06.E-09
glna-2	13.60	1.35	2.56	2.95.E-06
glr-2	3.41	2.44	5.41	1.87.E-05
glt-5	6.25	1.58	3.00	5.65.E-04
gly-2	7.84	1.21	2.32	3.71.E-04
gnrr-2	7.02	2.71	6.54	7.82.E-09
gpa-1	2.99	1.93	3.81	8.16.E-04
gpa-12	9.07	1.47	2.77	8.03.E-05
gpa-17	28.02	1.78	3.42	3.29.E-08
gpdh-1	9.22	1.42	2.68	4.68.E-05
gpx-6	3.19	1.60	3.02	6.05.E-03
grd-4	10.58	2.18	4.54	9.12.E-07
grl-17	1.62	6.74	107.23	5.22.E-03
grl-21	33.34	1.85	3.60	9.81.E-05
gst-15	9.03	1.83	3.54	3.39.E-05
gst-20	59.70	1.24	2.35	1.19.E-06
gst-24	319.08	4.91	30.15	1.13.E-93
gst-29	8.66	2.36	5.14	9.04.E-07
gst-3	3.99	2.10	4.29	8.05.E-04
gst-31	2.87	2.92	7.55	1.15.E-03

gst-33	4.76	3.64	12.51	2.00.E-06
gst-5	91.76	1.40	2.64	8.79.E-10
gstk-2	9.44	1.42	2.68	8.57.E-04
gsto-2	1.23	3.39	10.46	3.69.E-03
gtl-2	1.65	1.15	2.21	8.81.E-03
H01G02.3	4.61	1.01	2.01	7.05.E-03
H02F09.3	16.10	4.14	17.63	2.62.E-17
H05C05.4	8.67	1.38	2.61	4.14.E-03
H06I04.6	51.16	1.61	3.05	2.48.E-12
H10D18.5	7.02	1.23	2.35	9.14.E-04
H14E04.3	8.53	1.16	2.24	4.86.E-03
H39E23.3	1.66	2.87	7.32	1.49.E-05
hen-1	21.54	2.89	7.42	2.48.E-10
hil-1	29.79	2.07	4.21	4.16.E-09
hil-7	101.34	1.50	2.83	1.14.E-08
his-10	22.89	3.77	13.60	4.72.E-11
his-11	59.00	2.50	5.65	2.03.E-10
his-12	17.91	2.67	6.38	6.92.E-08
his-14	21.80	3.69	12.91	6.91.E-10
his-25	51.40	3.37	10.30	1.57.E-22
his-26	22.24	4.16	17.92	3.24.E-11
his-32	15.41	1.24	2.36	2.34.E-03
his-40	10.93	1.37	2.59	9.82.E-03
his-43	17.72	2.28	4.85	8.20.E-07
his-45	15.14	1.31	2.48	2.48.E-03
his-47	14.35	2.41	5.32	1.20.E-06
his-48	47.79	2.17	4.51	3.79.E-09
his-5	9.28	2.02	4.07	8.83.E-04
his-58	46.12	2.42	5.35	7.04.E-09
his-59	44.69	2.11	4.32	2.72.E-13
his-60	92.88	2.14	4.40	6.05.E-13
his-61	59.74	1.59	3.00	3.11.E-08
his-62	59.99	3.01	8.08	3.75.E-19
his-63	14.94	1.15	2.22	5.49.E-03
his-64	13.30	1.75	3.36	1.18.E-04
his-65	51.17	1.92	3.79	3.84.E-09
his-66	39.66	1.38	2.60	3.01.E-04
his-67	15.55	1.88	3.69	3.72.E-05
hot-5	5.62	2.22	4.65	1.65.E-04
hot-6	7.09	1.14	2.20	8.44.E-03
hpo-39	2.30	2.25	4.76	5.24.E-03
hrg-1	44.76	1.40	2.64	4.38.E-09
hsp-12.1	18.48	1.17	2.25	1.40.E-03
hsp-12.3	10.36	1.78	3.43	1.73.E-03
hsp-17	81.96	1.47	2.77	4.09.E-09
hsp-43	266.76	1.82	3.54	7.61.E-21
hsp-70	6.47	2.29	4.90	1.02.E-07
icmt-1	23.72	4.91	30.08	6.80.E-13
ifa-3	7.59	1.14	2.20	1.36.E-03
iglr-3	9.11	1.35	2.55	4.26.E-05
ilkp-1	6.82	1.26	2.39	2.10.E-03

ilys-2	32.02	3.83	14.24	3.52.E-15
ilys-3	9.13	3.81	14.01	1.95.E-07
ins-10	3.23	3.03	8.16	1.96.E-03
ins-17	39.01	1.25	2.38	6.60.E-05
ins-37	18.97	3.80	13.92	2.71.E-13
insc-1	7.52	1.22	2.33	4.15.E-04
inx-19	2.18	1.43	2.70	4.65.E-03
ipla-7	92.78	2.04	4.13	2.28.E-31
irg-1	19.11	2.67	6.35	4.28.E-08
irg-3	30.02	1.10	2.15	7.13.E-05
K01A2.10	15.91	1.30	2.47	4.69.E-04
K01A6.8	11.03	3.27	9.63	6.99.E-06
K01C8.1	86.69	1.03	2.04	2.80.E-08
K02A11.4	20.36	1.18	2.27	1.84.E-03
K02D10.2	2.45	3.09	8.53	6.63.E-03
K02D3.1	11.84	2.32	4.99	1.55.E-07
K03A11.5	10.51	1.36	2.56	5.79.E-04
K03D3.2	156.62	7.12	139.27	1.37.E-36
K05F1.10	57.89	1.95	3.86	3.19.E-10
K06A4.2	1.92	1.73	3.31	7.67.E-03
K06H7.2	5.24	1.07	2.11	5.22.E-03
K07C5.13	3.12	2.54	5.80	1.78.E-03
K08C7.1	10.62	1.25	2.38	2.14.E-04
K08C7.7	4.81	1.59	3.00	5.97.E-04
K08D10.14	6.55	1.74	3.35	6.97.E-05
K08D12.4	23.49	1.96	3.90	3.63.E-04
K08D8.11	7.07	1.75	3.36	9.02.E-03
K08D8.7	2.03	1.52	2.87	9.75.E-03
K09C8.7	6.60	1.60	3.03	2.94.E-03
K09F6.10	3.05	1.43	2.70	8.38.E-03
K09H9.5	9.05	1.39	2.63	2.10.E-03
K10B4.3	13.51	1.07	2.10	4.37.E-05
K10D11.2	8.70	2.48	5.57	1.47.E-08
K10G9.2	8.50	1.26	2.39	1.01.E-03
K11D12.9	11.51	2.71	6.53	7.43.E-10
K11H12.11	6.35	1.51	2.85	2.11.E-04
K11H3.2	5.21	2.90	7.47	9.35.E-06
kin-16	5.30	1.07	2.10	7.47.E-03
klp-4	17.92	1.11	2.16	5.29.E-09
lact-1	11.16	1.92	3.79	9.90.E-08
lec-11	39.80	1.19	2.28	9.27.E-07
lec-7	8.44	1.80	3.48	3.15.E-04
let-522	46.80	1.15	2.21	2.71.E-06
lgc-40	3.99	1.13	2.18	6.45.E-03
lgc-42	1.64	2.63	6.17	6.10.E-04
lgc-53	1.51	1.36	2.57	8.14.E-03
lim-8	11.07	1.07	2.11	4.83.E-06
linc-22	10.90	1.35	2.55	2.01.E-07
linc-39	4.03	3.22	9.33	2.66.E-03
linc-61	2.08	2.45	5.46	9.10.E-03
linc-91	0.35	2.21	4.62	8.49.E-03

lipl-4	3.29	2.65	6.26	4.82.E-05
lips-11	6.30	4.45	21.88	3.88.E-08
lips-15	82.54	1.03	2.04	1.10.E-06
lmd-4	0.70	1.85	3.60	8.36.E-04
lron-12	5.38	1.11	2.16	6.03.E-03
lys-3	107.77	3.68	12.82	2.37.E-29
lys-4	232.08	3.35	10.18	1.35.E-61
M01A8.1	8.14	1.58	3.00	2.50.E-04
M01G12.7	0.96	2.19	4.58	8.54.E-03
M01G12.9	30.66	2.36	5.12	4.85.E-20
M02E1.4	3.50	3.22	9.30	1.35.E-03
M02H5.8	309.78	1.28	2.42	4.86.E-12
M03A1.8	4.53	1.26	2.39	4.07.E-03
M04C3.2	3.56	1.38	2.60	1.13.E-03
M04D5.3	28.66	1.24	2.36	7.02.E-05
M04F3.3	20.78	1.17	2.26	3.32.E-05
M153.3	1.93	2.20	4.60	2.87.E-03
M199.9	54.65	3.59	12.07	1.22.E-11
madf-1	11.34	1.00	2.01	5.55.E-03
magu-2	18.29	1.20	2.30	1.51.E-06
magu-4	11.53	1.01	2.02	8.22.E-05
mai-1	32.40	1.35	2.55	1.96.E-06
mak-2	58.64	1.21	2.32	3.66.E-12
maph-9	2.06	1.83	3.55	1.28.E-03
mapk-15	4.32	1.40	2.65	8.81.E-04
math-10	1.38	2.27	4.83	6.93.E-03
math-15	2.61	1.72	3.29	3.92.E-03
math-21	1.90	2.76	6.76	5.70.E-05
math-28	2.59	2.61	6.09	4.66.E-05
math-41	13.08	1.74	3.34	2.03.E-06
max-1	10.18	1.37	2.58	1.65.E-06
mfsd-6	8.67	1.06	2.09	5.27.E-03
mlt-4	4.73	1.37	2.58	9.74.E-04
mrp-2	10.13	1.69	3.22	2.72.E-08
mrrf-1	33.80	1.02	2.03	1.52.E-04
msa-1	203.77	1.33	2.51	5.20.E-12
myo-5	13.77	1.33	2.51	9.37.E-08
nas-13	2.01	2.14	4.40	2.16.E-03
nas-38	5.33	1.60	3.03	4.72.E-03
nas-5	7.11	1.97	3.93	3.44.E-05
nceh-1	15.19	2.13	4.39	1.18.E-10
ncs-3	8.29	1.00	2.00	9.95.E-03
ncs-5	1.99	1.77	3.40	5.88.E-03
neto-1	2.16	1.63	3.10	6.99.E-03
nex-2	75.70	1.05	2.07	1.04.E-08
nex-3	104.12	1.54	2.92	8.91.E-16
nhr-11	5.15	1.90	3.74	4.45.E-06
nhr-112	9.30	1.22	2.33	6.85.E-04
nhr-116	3.30	1.49	2.81	9.36.E-04
nhr-123	2.03	2.37	5.15	3.52.E-04
nhr-132	8.13	1.25	2.38	1.15.E-03

nhr-142	2.61	2.12	4.35	1.14.E-04
nhr-150	1.07	2.20	4.61	4.56.E-03
nhr-154	10.45	1.16	2.24	5.62.E-04
nhr-170	4.01	1.25	2.38	3.49.E-03
nhr-178	2.15	2.03	4.09	2.23.E-03
nhr-206	1.72	1.62	3.07	5.49.E-03
nhr-213	3.58	1.86	3.64	1.47.E-04
nhr-247	3.15	3.38	10.40	1.59.E-04
nhr-44	5.45	1.18	2.26	7.74.E-03
nhr-54	2.16	1.94	3.84	2.98.E-03
nhr-56	5.56	1.03	2.05	4.27.E-03
nhx-6	14.43	5.40	42.32	1.29.E-29
nlp-60	2.32	2.77	6.81	5.82.E-03
nlp-61	34.39	2.29	4.88	1.34.E-08
nmad-1	76.10	1.14	2.21	4.69.E-09
nnt-1	10.95	1.72	3.31	2.84.E-10
nog-1	349.21	1.60	3.04	2.61.E-19
npax-2	2.74	2.01	4.02	6.17.E-03
nphp-1	2.55	1.78	3.43	1.86.E-04
nstp-6	11.19	1.47	2.76	1.79.E-05
numr-1	220.79	6.33	80.53	6.14.E-109
numr-2	227.90	6.53	92.37	4.36.E-110
oac-14	59.13	1.93	3.81	1.27.E-11
oac-24	0.69	2.58	5.97	2.44.E-03
oac-5	0.81	1.64	3.11	7.81.E-03
oac-55	0.98	1.45	2.74	7.16.E-03
oac-57	2.30	1.36	2.56	5.81.E-03
oac-7	1.88	1.45	2.73	1.85.E-03
oat-1	17.25	1.36	2.58	2.19.E-06
otpl-3	8.40	1.51	2.85	5.44.E-06
otpl-4	0.89	6.38	83.42	8.73.E-03
otpl-7	0.87	3.40	10.55	1.23.E-03
pals-14	22.66	4.15	17.79	6.56.E-21
pals-17	14.46	1.82	3.52	1.28.E-06
pals-18	6.78	1.44	2.72	2.20.E-03
pals-21	2.61	4.53	23.04	1.51.E-03
pals-23	17.36	1.24	2.36	3.95.E-04
pals-24	32.64	1.81	3.51	1.14.E-09
pals-26	9.25	2.29	4.89	6.18.E-06
pals-27	3.87	2.57	5.93	8.23.E-06
pals-32	8.21	4.61	24.49	2.51.E-09
pals-33	5.78	3.60	12.13	4.61.E-08
pals-37	1.90	1.86	3.64	1.06.E-03
pals-39	3.47	2.18	4.54	3.49.E-04
pals-40	2.86	3.57	11.92	2.10.E-04
pals-6	48.30	4.18	18.07	8.25.E-31
pde-5	1.08	2.05	4.14	1.19.E-03
pes-8	6.43	1.03	2.05	8.48.E-03
pgp-10	5.46	1.09	2.13	5.29.E-04
pgp-12	2.54	1.98	3.96	3.25.E-06
pgp-14	8.20	1.94	3.84	6.60.E-07

pgp-5	5.20	1.63	3.09	4.36.E-05
pgp-6	4.81	1.37	2.59	4.22.E-05
pgp-7	0.72	2.48	5.60	7.28.E-04
pgp-8	18.92	5.51	45.61	1.85.E-45
pgp-9	25.59	1.54	2.92	5.20.E-12
pgph-3	38.81	1.37	2.59	2.22.E-09
phat-3	10.74	1.46	2.75	6.49.E-05
phat-5	20.59	1.68	3.21	2.62.E-05
phg-1	20.73	1.58	2.98	3.46.E-04
phlp-2	0.86	1.51	2.86	4.73.E-03
piit-1	16.89	1.86	3.63	8.58.E-06
plep-1	2.70	5.84	57.29	1.55.E-05
pll-1	4.65	1.26	2.40	1.92.E-04
pmp-1	33.24	1.21	2.31	6.96.E-07
pmp-4	37.13	2.39	5.24	1.20.E-21
pqn-11	1.06	2.70	6.51	3.23.E-03
pqn-29	2.54	1.25	2.38	8.73.E-03
pqn-31	38.60	2.16	4.45	2.82.E-12
pqn-42	5.36	1.28	2.43	4.71.E-04
pqn-71	10.28	1.75	3.37	1.71.E-04
pqn-73	7.79	1.11	2.16	8.26.E-03
pqn-94	37.57	1.40	2.65	4.36.E-05
R01B10.3	39.24	1.10	2.15	2.58.E-04
R02D5.3	30.25	1.93	3.81	2.02.E-08
R02F2.1	660.61	1.64	3.11	1.09.E-18
R03H10.1	16.98	1.38	2.60	3.74.E-05
R03H10.6	26.84	5.14	35.22	3.19.E-33
R03H10.7	5.93	1.98	3.94	1.26.E-04
R04B5.6	1.64	2.64	6.21	1.12.E-03
R05D8.9	3.83	5.66	50.72	1.97.E-05
R06C1.6	13.05	1.32	2.49	2.18.E-04
R07A4.2	7.65	1.65	3.13	4.58.E-04
R07B1.9	3.48	1.47	2.76	7.32.E-04
R07B7.6	10.85	1.10	2.14	1.04.E-03
R07C12.1	6.85	2.21	4.62	2.67.E-06
R08E5.4	9.37	7.72	210.28	1.23.E-03
R09A1.2	5.30	1.42	2.68	7.90.E-04
R09A1.3	29.99	3.65	12.60	1.01.E-25
R09B5.11	23.83	1.82	3.52	7.79.E-09
R09H3.3	1.26	2.23	4.68	7.80.E-03
R10E8.8	22.05	2.51	5.69	1.20.E-10
R11D1.3	13.24	1.32	2.50	7.32.E-04
R11G1.2	2.39	2.17	4.50	7.52.E-03
R11H6.4	16.56	1.17	2.24	1.17.E-03
R13H4.8	4.58	1.65	3.13	7.26.E-03
R160.3	6.48	1.16	2.24	9.24.E-03
rbf-1	4.12	1.11	2.15	3.04.E-04
rhgf-2	12.49	1.00	2.01	8.66.E-06
ric-3	12.37	1.10	2.15	2.16.E-03
rig-5	2.97	1.31	2.48	4.92.E-03
rimb-1	7.96	1.03	2.05	3.10.E-04

rnt-1	1.78	1.59	3.02	6.32.E-03
rocf-1	2.18	1.34	2.53	9.15.E-04
rom-1	27.56	1.63	3.09	6.01.E-10
rom-2	1.59	2.10	4.28	6.88.E-03
rom-3	0.56	2.56	5.89	6.28.E-03
rpi-1	1.21	2.31	4.97	2.37.E-03
rpr-1	580.00	3.87	14.64	5.71.E-62
rrf-2	6.30	1.41	2.66	2.04.E-05
scl-12	13.98	8.94	492.54	1.50.E-04
scl-2	217.43	1.11	2.16	1.25.E-03
scl-23	2.59	3.48	11.15	8.93.E-05
scl-24	53.77	2.82	7.05	9.17.E-18
scl-25	12.89	4.38	20.81	1.19.E-13
scl-6	5.53	1.55	2.93	7.35.E-03
sdpn-1	25.18	1.26	2.40	2.18.E-07
sdz-35	3.64	3.97	15.62	2.13.E-06
sdz-6	55.37	3.01	8.05	1.86.E-17
seb-2	4.12	1.10	2.14	6.87.E-03
sel-7	4.55	1.40	2.65	1.82.E-03
sfxn-1.4	5.68	1.35	2.55	3.32.E-03
skr-3	148.17	1.19	2.28	1.40.E-09
skr-4	169.03	2.26	4.79	4.14.E-15
skr-5	21.59	2.50	5.66	3.10.E-05
slc-17.4	20.98	1.28	2.43	2.34.E-07
slc-17.9	4.21	1.68	3.20	2.25.E-04
slc-36.5	6.23	1.79	3.46	8.21.E-06
sma-3	13.69	1.05	2.08	3.25.E-03
snf-11	10.60	1.01	2.01	6.88.E-03
sodh-1	245.67	1.94	3.83	1.41.E-09
sol-2	5.67	1.16	2.23	2.39.E-03
spe-6	11.45	1.30	2.46	1.26.E-04
spg-20	26.25	1.17	2.25	1.40.E-07
spp-23	112.87	1.50	2.84	5.31.E-05
sptf-2	11.03	3.11	8.66	2.51.E-08
sqst-1	157.10	1.42	2.68	2.08.E-14
sqst-2	16.31	1.73	3.32	2.57.E-07
srab-4	1.96	1.74	3.34	8.63.E-03
srd-53	1.57	2.45	5.45	2.45.E-03
sri-16	3.14	3.34	10.14	4.72.E-05
sri-36	3.24	3.93	15.28	2.36.E-06
sri-38	2.94	3.85	14.39	2.62.E-05
sri-39	4.60	3.68	12.83	2.27.E-07
sri-74	3.20	5.18	36.33	5.90.E-07
srj-29	0.95	4.10	17.12	2.92.E-03
srp-2	39.41	1.31	2.48	3.77.E-07
srsx-25	3.06	3.15	8.91	5.06.E-06
srw-86	19.05	4.24	18.89	5.56.E-11
srx-13	3.55	7.60	194.08	1.47.E-03
stn-2	6.92	1.32	2.50	2.23.E-03
sto-3	3.02	3.96	15.51	1.13.E-04
str-144	1.31	3.31	9.94	1.62.E-03

svop-1	5.74	1.29	2.45	7.16.E-04
swm-1	52.30	1.02	2.03	8.85.E-04
syx-2	25.97	1.17	2.25	3.19.E-06
T01B10.5	5.53	1.76	3.38	1.08.E-03
T01C1.4	3.85	1.93	3.80	8.16.E-03
T01D3.6	144.76	2.25	4.76	8.93.E-31
T01E8.8	19.44	1.62	3.08	7.17.E-06
T03F6.4	4.96	1.31	2.48	7.92.E-03
T03G11.9	1.10	3.66	12.67	9.35.E-03
T04C12.1	10.79	2.01	4.03	1.80.E-04
T04D3.8	27.49	1.27	2.42	2.03.E-04
T05A7.11	2.76	2.75	6.74	6.39.E-06
T05A8.2	1.90	3.08	8.43	4.82.E-03
T05B11.1	18.71	1.19	2.28	1.58.E-04
T05B11.7	2.57	1.94	3.85	3.94.E-03
T05C12.11	1.62	2.08	4.23	7.56.E-03
T05H10.3	78.45	2.35	5.09	1.60.E-15
T06F4.1	7.17	1.33	2.51	1.65.E-03
T07A5.1	3.56	2.23	4.71	1.45.E-04
T07A5.7	3.45	2.92	7.54	2.40.E-06
T07D10.1	1.86	2.20	4.59	5.07.E-03
T07D10.3	4.56	1.30	2.47	1.55.E-03
T07D3.4	16.11	1.75	3.37	5.38.E-07
T07H6.1	2.74	1.53	2.90	4.08.E-04
T07H8.11	8.00	2.31	4.95	3.11.E-04
T08B1.4	13.19	1.14	2.20	1.15.E-04
T08D10.3	4.16	1.99	3.96	1.54.E-03
T08E11.1	2.50	2.91	7.49	7.48.E-06
T09F5.12	21.13	1.08	2.11	1.23.E-03
T10B10.8	12.80	2.07	4.21	2.31.E-10
T10B11.5	6.27	2.07	4.19	1.44.E-05
T10C6.15	2.36	1.63	3.10	9.97.E-03
T11B7.2	30.60	2.01	4.02	4.47.E-06
T11F9.12	29.41	1.22	2.32	4.28.E-09
T11G6.3	22.62	1.68	3.20	5.03.E-08
T12B3.3	146.46	1.16	2.23	1.36.E-10
T15B7.15	1.60	2.33	5.03	7.16.E-04
T16A9.3	1.92	1.93	3.80	1.88.E-03
T16G1.4	6.46	2.31	4.97	1.90.E-04
T16G1.5	3.80	2.14	4.41	8.12.E-06
T16G1.7	13.48	2.83	7.13	3.43.E-11
T17H7.1	21.12	2.36	5.12	1.10.E-09
T18D3.6	8.49	1.22	2.33	6.61.E-04
T19E7.6	22.31	1.06	2.08	3.22.E-03
T20D4.3	1.15	2.84	7.17	9.11.E-04
T20D4.5	2.67	2.95	7.73	3.53.E-07
T20D4.7	64.60	3.18	9.09	6.17.E-18
T20F5.4	15.66	1.60	3.02	5.54.E-07
T21C12.8	5.47	1.60	3.03	1.87.E-04
T22A3.12	4.16	5.17	36.08	1.95.E-04
T22B11.4	19.12	1.10	2.15	3.20.E-07

T22B7.3	47.52	1.17	2.25	8.17.E-05
T22B7.7	108.70	2.70	6.51	6.28.E-26
T22F3.11	3.50	2.29	4.89	2.29.E-04
T23B3.2	121.12	1.44	2.71	5.64.E-10
T23E1.3	3.15	3.14	8.84	1.20.E-04
T23F11.6	77.46	2.31	4.95	4.96.E-11
T23F2.2	4.63	1.08	2.11	1.26.E-03
T23F2.3	282.31	1.91	3.77	5.97.E-13
T24A6.7	4.75	4.25	19.01	1.21.E-05
T24C4.4	117.64	2.54	5.83	3.90.E-18
T24C4.8	12.99	3.16	8.96	1.95.E-09
T24E12.5	21.65	4.34	20.22	3.87.E-34
T24F1.4	5.07	2.65	6.28	3.87.E-04
T25C8.1	2.30	2.40	5.26	1.65.E-04
T26H5.10	9.92	1.61	3.06	5.58.E-03
T26H5.14	8.50	1.54	2.90	4.62.E-03
T26H5.9	246.86	1.83	3.56	3.85.E-25
T27C5.8	45.88	6.63	99.32	6.22.E-16
T27E4.1	2.86	1.44	2.72	7.31.E-03
T28A11.17	0.48	2.88	7.39	7.71.E-03
T28A11.19	14.37	1.81	3.50	6.45.E-06
T28A11.2	196.45	1.47	2.76	3.00.E-12
T28A11.25	13.74	1.52	2.87	9.02.E-05
T28A11.3	4.04	2.67	6.37	8.51.E-05
T28F3.5	3.12	2.26	4.78	1.96.E-08
tag-241	8.36	1.01	2.02	4.18.E-04
tag-243	12.55	1.00	2.00	9.28.E-03
tag-244	28.80	2.74	6.70	1.75.E-19
tat-2	5.88	1.13	2.19	6.98.E-04
tba-7	38.79	2.03	4.09	7.90.E-18
tbc-19	3.48	1.23	2.34	3.45.E-04
thn-1	38.38	5.78	54.83	1.98.E-21
thn-2	367.95	2.70	6.49	1.14.E-29
thn-5	10.85	2.44	5.41	5.73.E-07
tni-3	60.25	1.24	2.36	1.13.E-05
trpa-1	4.57	1.25	2.37	4.86.E-04
trpl-5	0.92	2.66	6.31	2.56.E-03
try-5	4.41	3.01	8.05	1.84.E-08
tsp-1	31.01	1.47	2.77	1.35.E-06
tsp-15	15.38	1.35	2.55	9.86.E-05
tsp-17	2.86	1.64	3.12	2.22.E-03
tsp-20	5.62	1.02	2.03	6.77.E-03
tsp-6	4.48	1.36	2.56	6.32.E-03
tth-1	286.79	1.19	2.28	4.29.E-08
ttr-14	22.72	1.67	3.19	2.45.E-06
ttr-15	1679.95	1.04	2.05	3.02.E-07
ttr-18	226.40	1.08	2.11	9.05.E-06
ttr-21	14.59	1.37	2.59	2.16.E-04
ttr-26	154.52	1.77	3.41	1.69.E-12
ttr-27	25.56	2.01	4.04	3.95.E-06
ttr-29	31.34	2.34	5.06	2.81.E-08

ttr-34	82.70	1.45	2.74	1.52.E-08
ttr-41	324.71	1.12	2.17	1.34.E-09
ttr-44	186.22	1.75	3.35	1.18.E-19
ttr-45	1275.67	2.73	6.65	1.01.E-50
ttr-48	181.36	1.11	2.16	3.37.E-09
ttr-51	2254.96	2.09	4.25	5.13.E-27
tts-2	123.97	1.34	2.53	6.38.E-04
twk-42	10.45	1.39	2.62	4.88.E-05
tyr-5	4.30	1.57	2.97	2.04.E-04
ubc-23	7.07	1.22	2.33	1.97.E-03
ugt-1	7.82	2.01	4.03	9.88.E-08
ugt-14	4.34	2.91	7.50	3.37.E-10
ugt-16	11.56	1.67	3.18	1.34.E-08
ugt-18	5.60	3.37	10.32	4.73.E-08
ugt-19	17.84	1.26	2.39	8.22.E-05
ugt-2	12.50	1.51	2.84	8.64.E-06
ugt-21	6.85	1.55	2.93	3.64.E-06
ugt-24	7.05	2.77	6.84	1.44.E-11
ugt-27	1.21	3.64	12.47	2.53.E-04
ugt-39	11.11	1.40	2.64	6.76.E-06
ugt-4	14.95	1.51	2.84	5.66.E-07
ugt-48	22.25	1.42	2.68	2.98.E-07
ugt-62	159.63	1.46	2.75	5.99.E-09
ugt-66	5.11	1.95	3.86	2.43.E-05
upb-1	228.94	1.27	2.41	7.10.E-11
valv-1	112.96	1.63	3.10	2.15.E-08
vamp-8	14.04	1.41	2.65	8.19.E-04
vap-2	1.73	1.96	3.90	1.50.E-03
ver-1	1.35	2.30	4.92	3.49.E-05
W01C9.1	4.05	3.44	10.86	2.30.E-05
W01C9.2	7.98	2.78	6.89	4.91.E-05
W01F3.2	190.99	1.13	2.19	1.21.E-08
W02A2.8	1.65	2.16	4.47	9.01.E-03
W02C12.2	10.37	1.46	2.75	9.97.E-04
W02D9.10	33.57	1.28	2.44	5.18.E-05
W02D9.6	266.42	2.11	4.33	6.57.E-23
W03D8.2	5.40	2.45	5.47	2.21.E-07
W03D8.7	2.03	2.00	4.01	5.17.E-03
W03D8.8	14.75	3.49	11.27	3.96.E-16
W03G1.5	3.30	2.36	5.12	1.48.E-06
W03G11.2	2.49	1.74	3.35	1.25.E-03
W04A4.2	10.05	1.18	2.26	1.81.E-03
W04C9.6	1.96	1.91	3.77	2.27.E-03
W04G5.8	2.51	3.99	15.92	1.10.E-06
W05H9.1	238.65	1.17	2.25	2.61.E-05
W05H9.3	384.12	2.12	4.35	4.00.E-32
W06D4.3	2.30	2.22	4.66	1.67.E-03
W06F12.3	1.29	2.70	6.50	2.47.E-03
W06G6.17	4.20	2.12	4.35	4.20.E-03
W06G6.20	9.76	1.87	3.67	2.26.E-04
W07B8.4	4.00	4.49	22.50	2.00.E-07

W07E6.3	3.37	2.33	5.01	4.16.E-05
W07E6.5	9.87	1.22	2.34	1.23.E-03
W07G1.25	53.30	4.71	26.10	1.61.E-15
W08E12.8	5.85	2.58	5.99	3.45.E-06
W09C3.3	1.54	2.36	5.13	4.98.E-03
W10C8.6	12.72	1.34	2.53	9.09.E-03
W10G11.2	18.68	1.69	3.22	1.24.E-04
W10G11.3	18.39	2.08	4.22	2.73.E-08
Y102A11A.2	9.15	1.88	3.69	2.29.E-07
Y102A11A.3	20.54	1.40	2.63	5.73.E-10
Y105C5A.12	102.64	2.81	7.00	9.73.E-07
Y105C5A.13	13.60	2.39	5.24	1.65.E-03
Y106G6D.6	19.30	1.01	2.01	4.04.E-03
Y110A2AL.4	36.07	1.62	3.07	9.68.E-07
Y119C1B.6	10.74	1.94	3.84	3.84.E-03
Y17D7B.3	1.13	3.03	8.14	9.33.E-03
Y17G7B.8	34.31	1.34	2.53	3.89.E-09
Y19D10A.11	3.35	1.55	2.93	1.16.E-03
Y22D7AL.15	164.57	1.86	3.63	3.94.E-20
Y23H5A.8	1.76	2.56	5.88	6.63.E-03
Y23H5B.8	6.80	1.42	2.68	5.80.E-03
Y24D9B.1	2.97	1.05	2.07	9.26.E-03
Y25C1A.14	24.02	1.39	2.62	8.13.E-05
Y26D4A.21	4.40	2.46	5.51	1.58.E-03
Y2H9A.4	3.50	1.53	2.89	1.60.E-03
Y32F6B.1	18.77	1.05	2.07	5.39.E-05
Y34F4.1	14.08	1.15	2.23	1.16.E-03
Y34F4.4	16.52	2.30	4.93	9.48.E-08
Y37D8A.16	86.66	1.53	2.89	6.00.E-08
Y37E11B.7	20.22	2.22	4.67	1.14.E-07
Y37H2A.10	2.40	4.05	16.61	3.89.E-03
Y37H2A.14	317.63	4.31	19.85	3.99.E-59
Y38C1AA.6	5.02	1.38	2.61	3.75.E-03
Y38E10A.14	35.84	1.58	3.00	1.90.E-08
Y38F1A.1	7.67	2.34	5.07	3.66.E-07
Y38H6C.15	3.60	3.04	8.20	2.52.E-04
Y38H6C.9	2.11	2.27	4.82	1.45.E-03
Y39A3A.3	1.20	2.76	6.77	6.45.E-03
Y39B6A.9	2.79	1.89	3.71	3.37.E-03
Y39H10A.1	38.43	5.81	56.03	6.10.E-16
Y40C7B.4	7.43	2.33	5.04	3.13.E-04
Y41D4A.1	1.06	3.74	13.38	7.06.E-03
Y41D4B.15	2.91	2.71	6.56	1.27.E-05
Y42A5A.3	17.00	1.90	3.74	1.80.E-05
Y43F8A.5	4.30	1.74	3.34	5.07.E-04
Y43F8B.12	7.16	5.03	32.71	2.55.E-06
Y43F8B.14	2.83	1.81	3.51	1.31.E-04
Y43F8B.15	4.56	4.43	21.59	1.62.E-06
Y43F8B.23	2.43	2.80	6.99	1.57.E-03
Y43F8B.25	1.22	3.85	14.45	8.46.E-03
Y44A6D.3	20.16	1.08	2.11	9.54.E-04

Y44E3A.1	8.69	1.15	2.21	1.81.E-03
Y45F10C.1	0.93	1.79	3.46	6.29.E-03
Y45F10D.6	19.65	3.05	8.27	7.50.E-11
Y45G5AM.3	14.14	1.65	3.15	2.85.E-07
Y46C8AM.1	7.31	3.50	11.32	1.00.E-07
Y47D3B.3	2.21	2.71	6.54	1.13.E-03
Y47H10A.3	10.40	3.91	15.07	3.47.E-12
Y47H10A.5	604.27	3.44	10.87	1.08.E-57
Y47H9B.2	2.55	1.83	3.57	2.07.E-03
Y48C3A.5	8.60	1.56	2.96	5.05.E-07
Y48G1BR.1	19.46	1.10	2.15	3.77.E-05
Y48G9A.7	22.23	2.22	4.65	3.31.E-05
Y49A3A.4	2.77	2.30	4.94	1.40.E-03
Y51A2D.21	16.60	1.00	2.01	8.55.E-03
Y51B9A.8	3.37	3.45	10.96	4.62.E-04
Y53G8AL.1	6.59	3.05	8.28	1.50.E-14
Y53G8AL.4	10.98	1.74	3.34	7.60.E-04
Y54E10A.17	80.26	1.32	2.50	7.82.E-10
Y54E5A.5	253.58	1.06	2.09	3.26.E-09
Y54G2A.28	2.61	2.61	6.09	3.43.E-03
Y54G2A.52	25.64	1.08	2.12	1.94.E-03
Y54G2A.57	6.71	4.26	19.22	1.91.E-09
Y55B1BR.6	7.50	1.23	2.35	9.79.E-03
Y55F3BR.11	20.42	1.95	3.86	1.83.E-06
Y57E12AR.1	16.48	1.25	2.37	2.05.E-03
Y57E12B.11	6.01	6.34	80.78	8.46.E-03
Y57G11B.5	334.86	1.42	2.68	9.00.E-08
Y57G11C.40	12.58	1.80	3.48	5.02.E-05
Y57G11C.5	2.13	1.75	3.36	7.56.E-04
Y57G11C.8	21.33	1.26	2.39	2.41.E-04
Y58A7A.3	74.34	1.89	3.71	1.34.E-13
Y60C6A.3	15.16	6.09	68.26	5.91.E-06
Y65B4BR.1	6.86	2.68	6.40	1.87.E-08
Y66A7A.4	1.05	1.82	3.52	6.25.E-03
Y66C5A.1	8.35	1.58	2.99	4.13.E-03
Y66D12A.24	23.30	1.02	2.03	8.90.E-04
Y69A2AR.12	3.54	2.31	4.96	3.62.E-05
Y69A2AR.25	6.05	1.77	3.40	3.92.E-04
Y69H2.10	1.23	1.96	3.90	5.44.E-04
Y6E2A.4	2.28	2.45	5.45	2.63.E-04
Y71G12B.2	3.49	1.99	3.96	6.23.E-04
Y71G12B.30	3.38	2.12	4.36	5.87.E-04
Y71G12B.5	0.45	1.99	3.98	6.71.E-03
Y71H2AM.12	9.06	1.26	2.39	4.00.E-04
Y71H2B.1	11.89	1.11	2.16	7.01.E-03
Y73B6BL.12	10.67	1.60	3.02	3.79.E-04
Y75B7B.1	1.15	2.30	4.94	3.59.E-03
Y75B8A.23	20.96	4.26	19.22	6.81.E-11
Y75B8A.32	2.83	1.88	3.68	1.05.E-03
Y75B8A.33	1.27	3.07	8.43	1.74.E-03
Y76B12C.4	12.99	2.55	5.85	1.06.E-08

Y77E11A.12	4.82	1.42	2.68	2.85.E-04
Y77E11A.2	76.09	1.23	2.34	3.64.E-11
Y82E9BL.18	8.44	2.97	7.81	8.13.E-10
Y82E9BR.5	1.88	1.71	3.28	5.50.E-03
Y87G2A.16	1.58	1.96	3.88	3.00.E-03
Y94H6A.2	3.70	2.83	7.09	1.80.E-05
Y97E10AR.1	14.55	1.87	3.66	6.83.E-09
Y9C9A.16	1.79	2.74	6.66	4.01.E-04
ZC15.3	1.55	3.15	8.89	2.26.E-03
ZC239.14	18.40	3.60	12.11	4.08.E-15
ZC239.15	2.81	2.29	4.88	6.38.E-04
ZC239.22	7.06	1.60	3.03	2.04.E-03
zig-13	1.06	3.34	10.10	4.55.E-03
zig-3	5.31	1.83	3.55	1.44.E-03
zig-4	15.10	1.95	3.86	5.69.E-06
zig-5	7.70	1.09	2.13	6.47.E-03
zip-6	1.99	2.45	5.45	6.60.E-03
zipt-2.3	5.75	1.12	2.17	7.66.E-03
ZK1010.4	5.82	1.33	2.52	9.67.E-03
ZK1037.6	19.78	2.02	4.05	4.44.E-11
ZK1055.6	38.78	2.41	5.33	1.85.E-19
ZK1055.7	213.97	3.62	12.29	2.32.E-61
ZK1290.10	16.87	2.85	7.19	2.00.E-14
ZK1290.14	23.81	1.07	2.11	1.77.E-03
ZK1290.5	31.85	2.03	4.08	4.16.E-12
ZK1320.11	34.52	1.39	2.62	6.17.E-07
ZK177.9	11.86	4.08	16.87	1.88.E-09
ZK180.7	1.96	2.40	5.26	6.95.E-03
ZK262.2	2.79	1.72	3.29	1.48.E-03
ZK353.4	1.98	2.42	5.34	1.20.E-03
ZK355.8	10.83	3.92	15.15	1.09.E-05
ZK384.7	38.30	1.58	3.00	1.69.E-05
ZK418.13	4.69	2.48	5.57	4.87.E-06
ZK546.4	6.22	2.51	5.70	3.71.E-04
ZK593.2	10.12	1.74	3.34	1.75.E-04
ZK596.1	84.97	2.92	7.58	4.92.E-20
ZK6.8	4.00	1.06	2.09	8.05.E-03
ZK675.4	9.23	2.19	4.57	5.99.E-06
ZK792.4	3.04	1.53	2.90	2.66.E-03
ZK822.1	2.78	1.26	2.39	1.34.E-03
ZK822.2	109.18	1.01	2.01	7.74.E-07
ZK863.8	25.33	1.60	3.02	1.28.E-05
ZK896.4	17.37	2.46	5.52	5.27.E-11
ZK896.5	28.56	2.39	5.23	6.28.E-17
ZK899.1	17.34	1.86	3.63	4.44.E-06
ZK930.2	39.67	1.33	2.51	1.36.E-05
zmp-1	6.44	2.70	6.51	3.95.E-08
zmp-3	12.19	1.08	2.12	4.95.E-04

Supplementary Table4

RNA sequencing analysis of animals exposed to LMB at 2°C for 3 hr.

(logFC<-1 p<0.01)

Name	Max group mean	Log_2 fold change	Fold change	P-value
abu-1	3.00	-2.26	-4.79	4.84.E-03
abu-15	2.80	-2.64	-6.25	1.75.E-03
abu-7	1.02	-6.51	-91.31	7.39.E-03
acd-1	2.05	-2.08	-4.23	1.12.E-04
acdh-1	263.41	-3.23	-9.38	9.54.E-10
acdh-2	3.46	-1.59	-3.01	5.29.E-03
acl-7	10.70	-1.35	-2.55	2.11.E-06
acox-1.1	20.00	-1.01	-2.02	7.49.E-05
acox-1.2	10.58	-2.62	-6.15	7.11.E-12
alh-13	22.40	-1.59	-3.02	4.14.E-08
aman-1	10.95	-1.05	-2.08	1.12.E-05
amt-4	17.30	-1.68	-3.20	1.54.E-09
anp-1	9.30	-1.03	-2.04	2.05.E-04
apm-3	21.74	-1.13	-2.18	3.15.E-06
aptf-4	9.02	-1.34	-2.54	1.02.E-04
aqp-8	23.30	-2.02	-4.06	1.01.E-11
argk-1	5.80	-3.24	-9.43	9.37.E-11
arrd-1	26.73	-1.28	-2.43	1.94.E-03
asm-3	2.76	-2.49	-5.61	2.21.E-06
asp-13	53.12	-2.82	-7.04	1.47.E-46
atic-1	15.10	-1.35	-2.56	1.86.E-08
B0025.5	2.24	-4.04	-16.48	5.56.E-03
B0035.13	51.43	-2.38	-5.21	3.23.E-25
B0244.17	1.26	-1.92	-3.79	9.83.E-03
B0294.1	6.48	-2.92	-7.57	5.16.E-05
B0416.4	20.07	-1.03	-2.04	2.63.E-03
bath-34	2.84	-1.37	-2.59	7.42.E-03
best-7	4.70	-2.29	-4.89	6.99.E-08
bli-1	3.14	-2.69	-6.47	4.07.E-05
bli-2	4.80	-3.66	-12.62	2.19.E-05
bli-6	10.73	-4.32	-19.97	2.21.E-08
btb-7	4.76	-1.20	-2.29	4.93.E-03
C01G10.6	3.92	-2.94	-7.65	2.23.E-03
C02F5.14	8.51	-1.79	-3.45	4.99.E-04
C04E12.5	0.64	-2.55	-5.85	5.28.E-03
C05D12.3	14.26	-1.07	-2.10	1.16.E-07

C05D12.4	5.13	-1.86	-3.63	2.84.E-04
C05D12.5	6.29	-2.00	-4.00	3.09.E-04
C05G5.7	4.77	-4.92	-30.35	2.84.E-07
C06A5.12	10.12	-1.43	-2.69	1.14.E-03
C07D8.6	266.77	-1.08	-2.12	3.86.E-07
C08B6.11	2.22	-1.15	-2.22	9.17.E-03
C08F11.1	3.54	-2.28	-4.84	4.10.E-04
C11H1.5	0.90	-1.93	-3.81	8.21.E-03
C11H1.9	9.72	-1.02	-2.03	2.44.E-03
C14C6.2	27.13	-3.68	-12.77	6.27.E-23
C14C6.5	98.04	-3.60	-12.17	2.94.E-37
C17D12.7	25.45	-1.02	-2.03	1.96.E-05
C17E7.12	64.80	-1.02	-2.03	3.39.E-04
C17H12.6	5.38	-3.74	-13.37	4.98.E-13
C18A11.3	8.16	-1.81	-3.51	1.79.E-05
C23H4.3	3.38	-1.22	-2.33	1.12.E-03
C23H5.8	85.11	-3.49	-11.22	9.82.E-70
C27A2.12	1.79	-1.36	-2.57	8.88.E-03
C27C12.3	13.49	-1.52	-2.88	8.62.E-05
C29F3.7	23.49	-1.58	-2.98	6.40.E-12
C30G12.10	10.73	-1.44	-2.71	3.75.E-03
C30G12.2	4.37	-2.71	-6.54	8.31.E-06
C31G12.1	7.34	-1.37	-2.59	1.28.E-03
C32H11.11	3.48	-7.15	-142.03	3.89.E-03
C32H11.5	3.19	-1.90	-3.73	4.02.E-03
C32H11.9	18.29	-6.24	-75.39	1.58.E-19
C34F6.1	2.18	-1.97	-3.92	2.38.E-04
C35A11.4	8.01	-1.55	-2.92	1.34.E-05
C35A5.3	13.32	-2.04	-4.12	2.15.E-06
C35D6.4	2.05	-2.18	-4.53	3.61.E-03
C40H1.7	10.67	-3.16	-8.91	1.34.E-14
C44C10.3	0.87	-4.77	-27.36	5.71.E-04
C45E5.1	8.80	-2.03	-4.07	4.88.E-08
C45H4.14	16.06	-1.24	-2.37	1.44.E-03
C46E10.8	1.74	-1.31	-2.48	9.76.E-03
C49F5.3	27.51	-1.47	-2.77	5.44.E-07
C53A5.16	14.37	-1.03	-2.04	1.51.E-03
C53D5.5	12.52	-1.01	-2.02	1.18.E-05
C55B6.1	12.30	-1.34	-2.53	1.81.E-06
C55B7.3	3.36	-1.70	-3.24	3.27.E-04

car-1	820.91	-1.29	-2.44	8.14.E-06
cbl-1	33.15	-2.18	-4.52	8.73.E-17
ccch-2	3.61	-1.56	-2.95	3.17.E-03
cct-8	156.13	-1.10	-2.15	8.14.E-09
CD4.10	12.29	-1.10	-2.14	8.01.E-03
cdc-25.3	40.54	-1.15	-2.22	1.04.E-04
cdd-2	55.64	-1.31	-2.48	8.35.E-07
cdh-10	2.83	-2.05	-4.13	1.59.E-04
cdh-12	4.50	-1.91	-3.77	1.56.E-06
cdh-7	1.62	-1.90	-3.72	5.92.E-04
cdk-4	12.26	-1.26	-2.39	4.43.E-04
cdo-1	36.52	-1.22	-2.33	5.93.E-06
ced-8	5.27	-1.23	-2.34	3.71.E-03
ceh-21	11.04	-1.00	-2.00	2.74.E-04
cey-2	1523.12	-1.18	-2.26	4.03.E-07
cgh-1	778.53	-1.21	-2.31	2.36.E-06
chil-22	2.65	-3.30	-9.87	2.72.E-08
chtl-1	28.97	-1.59	-3.02	1.45.E-08
ckb-4	10.35	-1.01	-2.02	2.00.E-03
clec-12	3.33	-1.55	-2.93	6.29.E-04
clec-166	6.25	-2.25	-4.77	2.50.E-07
clec-186	64.19	-1.78	-3.43	6.60.E-19
clec-187	12.20	-1.03	-2.05	4.68.E-04
clec-205	2.40	-2.38	-5.22	1.07.E-06
clec-209	190.28	-2.75	-6.73	1.74.E-45
clec-218	5.59	-2.93	-7.64	8.37.E-07
clec-26	1.08	-2.68	-6.41	8.94.E-04
clec-4	11.42	-2.24	-4.73	1.71.E-09
clec-48	14.62	-1.85	-3.60	1.72.E-09
clec-53	2.14	-1.85	-3.60	1.87.E-03
clec-54	4.01	-2.23	-4.70	8.11.E-07
clec-65	138.57	-1.22	-2.33	7.09.E-11
clec-7	3.67	-3.75	-13.47	9.74.E-10
clec-85	167.99	-1.71	-3.27	1.56.E-18
cnc-2	48.11	-1.39	-2.61	3.93.E-04
coh-4	12.80	-1.10	-2.14	1.76.E-04
col-10	341.82	-1.45	-2.74	9.99.E-03
col-110	1.35	-2.52	-5.75	2.76.E-03
col-117	318.90	-1.89	-3.70	9.27.E-05
col-120	3.44	-3.01	-8.08	8.88.E-05

col-125	207.89	-2.18	-4.54	3.96.E-04
col-13	24.78	-2.37	-5.16	2.81.E-03
col-133	10.40	-4.87	-29.21	1.70.E-08
col-138	17.75	-3.82	-14.10	8.05.E-09
col-144	273.31	-1.54	-2.91	4.83.E-03
col-147	61.78	-2.05	-4.13	2.71.E-04
col-149	37.46	-4.41	-21.22	1.27.E-12
col-154	28.11	-2.69	-6.43	2.06.E-05
col-155	30.11	-2.00	-4.01	8.51.E-09
col-156	11.04	-4.66	-25.35	3.56.E-09
col-157	12.67	-2.82	-7.08	1.41.E-05
col-158	11.56	-2.05	-4.15	9.38.E-07
col-161	28.66	-5.98	-63.30	1.25.E-12
col-162	19.05	-4.71	-26.23	2.12.E-13
col-166	276.28	-1.65	-3.13	8.06.E-04
col-168	72.79	-2.04	-4.13	8.88.E-04
col-169	53.96	-3.53	-11.53	1.00.E-09
col-17	242.02	-7.24	-151.02	3.35.E-21
col-170	19.69	-3.48	-11.14	6.89.E-09
col-173	6.71	-3.40	-10.59	7.39.E-07
col-175	9.23	-6.56	-94.59	7.24.E-08
col-180	24.25	-3.62	-12.27	3.61.E-09
col-38	19.56	-2.26	-4.79	2.29.E-07
col-39	53.28	-1.57	-2.96	4.92.E-03
col-41	102.30	-6.82	-112.94	1.45.E-21
col-43	6.85	-2.00	-3.99	9.29.E-06
col-46	3.46	-3.02	-8.10	6.14.E-06
col-49	7.81	-5.80	-55.65	6.29.E-09
col-54	13.03	-2.28	-4.85	3.31.E-04
col-63	7.40	-3.46	-10.97	2.44.E-07
col-71	20.55	-2.78	-6.89	8.25.E-07
col-73	176.62	-6.99	-127.53	3.09.E-21
col-77	43.08	-1.95	-3.87	4.54.E-05
col-90	85.43	-7.61	-195.87	3.00.E-23
col-91	20.37	-2.95	-7.74	2.82.E-05
col-92	61.24	-3.15	-8.85	9.50.E-10
col-93	286.74	-1.65	-3.13	1.01.E-05
comt-4	4.02	-2.88	-7.37	5.26.E-05
cpr-6	525.38	-1.32	-2.49	1.57.E-06
cth-1	21.75	-1.13	-2.18	4.97.E-05

ctsa-1	9.29	-3.39	-10.49	9.28.E-17
ctsa-2	26.64	-2.01	-4.02	9.02.E-14
cyp-25A1	10.31	-3.12	-8.70	1.77.E-18
cyp-35C1	6.66	-1.22	-2.32	1.76.E-04
cyp-36A1	5.74	-1.02	-2.03	1.87.E-03
cyp-37B1	7.47	-1.01	-2.02	2.90.E-03
D1014.2	2.66	-1.82	-3.52	5.51.E-04
D1086.7	18.45	-1.12	-2.18	2.28.E-04
dao-4	1.88	-4.81	-28.07	1.60.E-03
dct-11	30.53	-1.11	-2.16	6.17.E-07
dct-16	741.30	-1.27	-2.41	1.82.E-12
dct-5	1.42	-2.37	-5.19	6.62.E-03
dct-7	9.62	-2.57	-5.92	2.55.E-03
ddo-1	4.52	-1.31	-2.48	1.85.E-03
dhs-25	41.78	-1.33	-2.51	2.18.E-07
dod-21	23.89	-6.82	-113.00	7.04.E-19
dod-22	18.47	-2.15	-4.45	2.06.E-13
dod-24	55.84	-4.47	-22.20	4.82.E-67
dpy-13	234.67	-6.87	-117.04	6.45.E-19
dpy-20	1.85	-5.93	-60.89	2.99.E-05
dpy-4	192.88	-6.35	-81.55	1.57.E-19
dpy-5	213.41	-7.29	-156.85	5.97.E-22
dpy-6	2.85	-1.93	-3.81	4.55.E-05
dpy-8	24.14	-5.24	-37.81	2.30.E-19
dpy-9	16.40	-1.67	-3.19	3.02.E-04
drd-1	7.89	-2.79	-6.92	3.38.E-11
drd-10	36.74	-2.81	-7.01	1.83.E-05
drd-5	8.04	-1.44	-2.72	1.56.E-04
dsl-1	6.97	-1.37	-2.59	5.15.E-03
dsl-2	27.38	-1.16	-2.24	3.40.E-04
E01G4.6	6.49	-4.08	-16.91	1.76.E-10
E01G6.3	1.86	-2.34	-5.07	1.54.E-05
E02H4.6	33.44	-1.21	-2.31	1.81.E-05
E02H4.7	5.63	-1.66	-3.16	1.03.E-03
E04F6.15	4.11	-3.76	-13.57	5.97.E-10
ech-6	259.50	-1.01	-2.01	5.22.E-08
EEED8.12	12.70	-1.08	-2.11	6.64.E-03
EEED8.4	15.62	-1.11	-2.15	2.23.E-03
egas-3	0.80	-3.27	-9.62	2.81.E-05
ent-4	6.11	-1.34	-2.54	6.16.E-05

ent-5	21.19	-1.42	-2.68	6.03.E-06
F01D5.1	38.06	-2.32	-4.99	1.92.E-15
F01D5.2	5.88	-3.89	-14.78	9.06.E-08
F01D5.3	12.70	-3.29	-9.75	2.20.E-13
F01D5.5	40.01	-2.77	-6.81	5.90.E-17
F02E8.4	24.20	-1.10	-2.15	2.56.E-04
F08D12.2	5.61	-2.50	-5.65	1.02.E-06
F09B12.3	45.19	-1.11	-2.16	3.40.E-05
F10C1.9	6.67	-2.26	-4.78	3.18.E-07
F11C7.2	9.10	-1.48	-2.79	1.38.E-03
F11C7.7	2.23	-3.02	-8.13	5.88.E-03
F12E12.1	50.95	-1.01	-2.02	1.63.E-04
F14D2.14	3.43	-1.52	-2.86	6.26.E-03
F14H3.3	16.96	-1.61	-3.06	1.56.E-05
F15A4.10	27.60	-1.12	-2.17	1.63.E-03
F15E6.3	78.00	-1.63	-3.09	2.16.E-04
F15E6.4	74.92	-1.23	-2.35	4.67.E-03
F19B10.2	31.54	-1.06	-2.08	5.52.E-05
F19H6.4	55.37	-1.27	-2.42	2.67.E-06
F21C10.9	3.07	-1.78	-3.44	2.11.E-04
F22F4.4	27.48	-1.02	-2.03	4.19.E-05
F22F7.8	14.53	-1.26	-2.39	6.45.E-04
F22H10.6	11.42	-1.10	-2.15	2.13.E-03
F23D12.2	3.98	-1.30	-2.46	1.70.E-04
F25B4.8	8.91	-1.77	-3.40	8.68.E-06
F28H7.3	58.81	-1.29	-2.45	1.78.E-09
F31B9.3	17.64	-1.01	-2.01	2.28.E-03
F31F6.1	10.22	-1.19	-2.28	7.69.E-03
F32A5.3	19.20	-1.20	-2.30	3.20.E-05
F36H5.13	3.07	-1.18	-2.26	8.46.E-03
F37A4.5	1.49	-2.07	-4.21	5.10.E-03
F39F10.4	7.17	-1.34	-2.54	2.80.E-03
F42A10.9	11.23	-1.21	-2.31	1.51.E-03
F42A9.6	58.64	-1.08	-2.12	4.29.E-04
F45D11.14	86.25	-2.15	-4.42	1.02.E-08
F47G3.3	9.77	-1.16	-2.23	1.29.E-04
F49C12.14	7.30	-1.88	-3.67	4.44.E-05
F49F1.5	7.98	-1.97	-3.92	7.23.E-05
F49F1.7	18.39	-2.75	-6.71	1.70.E-20
F52F10.2	1.22	-2.42	-5.37	1.92.E-03

F55G11.4	79.27	-2.13	-4.38	3.09.E-23
F56A4.2	189.92	-2.72	-6.58	5.39.E-44
F56C9.7	123.70	-1.09	-2.13	2.53.E-09
F57B1.9	4.47	-1.25	-2.37	3.81.E-03
F57F4.4	82.73	-1.22	-2.34	1.43.E-10
F58A6.9	2.89	-3.23	-9.41	3.35.E-03
F58G6.3	31.73	-3.23	-9.38	1.82.E-13
F58G6.7	19.03	-2.46	-5.50	1.01.E-10
fbxa-120	4.26	-1.20	-2.29	4.62.E-03
fbxa-192	19.41	-1.07	-2.10	9.94.E-04
fbxa-83	8.69	-1.24	-2.36	1.02.E-03
fbxb-16	1.50	-3.04	-8.25	1.96.E-03
fbxb-17	4.99	-1.35	-2.55	2.13.E-03
fbxb-26	4.00	-1.44	-2.71	2.89.E-03
fbxb-44	4.81	-1.67	-3.19	4.13.E-04
fbxb-52	1.70	-2.90	-7.44	6.42.E-04
fbxb-54	4.02	-1.40	-2.64	8.72.E-03
fbxb-59	1.44	-2.08	-4.23	6.62.E-03
fbxb-67	4.96	-1.56	-2.94	1.42.E-03
fbxc-32	12.65	-1.11	-2.16	1.35.E-03
fbxc-42	5.19	-1.71	-3.27	5.14.E-05
fil-2	4.39	-1.32	-2.49	7.11.E-03
fipr-13	10.22	-2.03	-4.07	1.16.E-04
fkh-4	7.97	-1.22	-2.34	1.95.E-04
flh-3	39.45	-1.03	-2.04	8.52.E-05
flr-1	2.30	-1.13	-2.19	5.94.E-03
folt-2	74.04	-3.88	-14.72	1.23.E-51
fpn-1.2	4.13	-1.90	-3.72	7.78.E-06
gale-1	86.37	-1.39	-2.62	1.58.E-13
gcsh-1	24.36	-1.19	-2.28	1.11.E-03
gei-18	1.05	-2.26	-4.78	7.33.E-03
gfi-1	59.05	-1.29	-2.45	3.94.E-11
gipc-1	2.72	-1.72	-3.28	9.47.E-03
glp-4	78.32	-1.05	-2.06	5.30.E-09
got-2.1	60.40	-1.56	-2.96	1.88.E-12
gpd-4	215.72	-1.17	-2.25	3.69.E-07
gpx-7	6.47	-1.47	-2.76	6.60.E-04
grd-1	1.78	-1.44	-2.71	2.46.E-03
grd-6	5.68	-3.08	-8.46	3.46.E-07
gst-13	53.67	-1.13	-2.20	2.13.E-05

gst-27	46.52	-1.46	-2.75	1.89.E-08
gst-41	2.62	-3.60	-12.14	1.04.E-05
gsto-1	5.41	-1.72	-3.30	8.12.E-04
H20E11.3	4.71	-3.33	-10.04	8.40.E-10
H23N18.5	12.17	-2.37	-5.18	1.75.E-04
H25K10.1	1.39	-4.35	-20.36	2.51.E-05
H37A05.2	1.78	-3.02	-8.10	2.40.E-05
hil-5	250.68	-1.25	-2.39	2.20.E-06
hphd-1	88.08	-2.57	-5.95	1.38.E-33
hrpk-1	153.18	-1.11	-2.15	2.39.E-07
hsp-1	1476.11	-1.16	-2.24	4.15.E-07
hsp-60	307.07	-1.47	-2.77	1.10.E-07
ifc-1	14.33	-1.15	-2.21	8.37.E-03
ifet-1	261.83	-1.33	-2.52	4.24.E-09
ilys-5	173.34	-1.56	-2.94	1.06.E-05
inf-1	355.67	-1.00	-2.01	2.27.E-07
ins-19	6.10	-7.21	-148.25	2.57.E-03
irg-4	10.63	-3.08	-8.43	1.30.E-13
K02E11.10	11.78	-6.09	-67.99	2.48.E-13
K02E11.6	2.60	-1.73	-3.33	3.62.E-03
K02E11.7	7.30	-1.37	-2.59	1.91.E-03
K03H6.2	12.41	-1.40	-2.64	6.09.E-05
K04C1.5	51.30	-1.15	-2.22	1.01.E-04
K08D8.5	40.54	-3.27	-9.64	3.39.E-41
K08E7.5	3.33	-1.40	-2.64	5.36.E-03
K08H2.3	15.97	-1.29	-2.44	6.19.E-04
K09C4.5	5.82	-1.66	-3.15	1.82.E-06
K09E3.7	5.81	-1.60	-3.03	1.86.E-05
K10C2.1	14.06	-1.14	-2.21	2.71.E-06
K10D11.5	12.00	-1.12	-2.17	2.41.E-05
K10D11.6	3.92	-1.57	-2.97	2.40.E-05
K11D12.13	74.94	-2.24	-4.72	1.51.E-27
lbp-8	2.52	-1.97	-3.91	2.81.E-03
lido-2	4.36	-1.23	-2.34	4.21.E-03
lido-5	4.18	-1.16	-2.23	5.07.E-03
lin-42	15.70	-1.84	-3.59	1.76.E-05
lipl-2	10.19	-2.02	-4.06	2.13.E-09
LLC1.2	126.26	-1.53	-2.90	1.30.E-18
lon-1	48.62	-1.28	-2.43	9.51.E-10
lon-3	19.02	-4.43	-21.49	1.09.E-11

lys-6	1.61	-3.15	-8.90	9.30.E-04
M151.2	1.60	-1.81	-3.51	7.74.E-03
M151.4	1.68	-2.33	-5.04	2.41.E-05
M151.7	28.03	-1.09	-2.13	6.13.E-04
marg-1	4.87	-1.52	-2.88	1.17.E-04
math-45	1.63	-2.66	-6.32	1.03.E-05
meg-1	100.84	-1.14	-2.21	3.15.E-06
meg-3	35.88	-1.28	-2.43	6.67.E-08
meg-4	22.77	-1.31	-2.48	4.70.E-08
mes-1	9.39	-1.13	-2.18	9.25.E-05
mlt-10	20.99	-1.23	-2.35	2.18.E-04
mrps-21	43.81	-1.08	-2.12	3.43.E-04
msp-152	4.18	-2.28	-4.87	7.50.E-03
msp-33	4.18	-2.59	-6.03	2.37.E-03
msp-49	3.06	-2.83	-7.13	9.22.E-03
msp-56	4.93	-2.12	-4.36	7.73.E-03
mtl-2	89.35	-3.71	-13.07	4.38.E-41
mtp-18	11.97	-1.07	-2.09	3.36.E-03
nas-15	3.08	-1.74	-3.35	3.83.E-03
nas-3	2.11	-3.07	-8.42	2.68.E-05
nas-37	8.71	-2.12	-4.36	7.64.E-08
nas-4	1.65	-1.79	-3.45	1.87.E-03
ncx-6	2.13	-1.29	-2.45	6.28.E-03
ncx-7	5.76	-1.09	-2.13	9.83.E-04
ncx-8	1.21	-1.93	-3.80	1.34.E-03
ncx-9	1.47	-3.26	-9.56	4.34.E-06
nep-18	1.12	-2.35	-5.11	8.36.E-05
nhr-114	12.41	-1.53	-2.89	2.62.E-06
nhr-68	21.85	-1.50	-2.82	2.25.E-08
nlp-28	64.04	-1.08	-2.11	1.98.E-03
nspc-16	30.24	-2.00	-4.01	9.89.E-09
nspc-17	21.84	-3.32	-10.00	2.23.E-11
nspc-18	18.86	-2.61	-6.09	1.14.E-08
nspc-19	14.97	-3.18	-9.06	1.62.E-07
nspc-20	124.11	-1.05	-2.07	9.21.E-05
oac-20	9.88	-5.39	-42.06	2.59.E-21
oac-29	0.58	-3.30	-9.82	2.16.E-03
oac-30	0.79	-2.50	-5.67	2.69.E-03
oac-31	4.50	-1.15	-2.22	3.08.E-03
oac-32	2.08	-1.55	-2.92	1.21.E-03

oac-58	0.91	-5.35	-40.76	4.24.E-05
orc-3	34.70	-1.07	-2.10	1.27.E-06
oxa-1	31.67	-1.07	-2.10	4.21.E-05
papl-1	19.08	-1.76	-3.39	1.70.E-14
pcp-1	15.11	-1.06	-2.09	3.78.E-04
pcp-2	13.69	-2.93	-7.60	9.05.E-24
pcp-4	7.76	-1.29	-2.45	1.02.E-03
pept-1	17.33	-1.13	-2.19	9.53.E-05
pes-1	5.22	-2.15	-4.44	4.57.E-06
pes-10	7.63	-1.77	-3.40	1.61.E-05
pgp-13	0.46	-2.97	-7.82	1.24.E-04
pho-11	108.58	-2.48	-5.59	2.24.E-11
pho-13	2.60	-1.33	-2.51	7.65.E-03
pif-1	25.79	-1.19	-2.29	3.64.E-06
pmp-5	11.79	-1.90	-3.74	4.14.E-10
pqn-54	2.92	-1.58	-3.00	1.02.E-03
pud-1.2	722.98	-1.61	-3.05	1.14.E-08
pud-2.1	383.80	-1.94	-3.83	1.22.E-09
pud-2.2	385.41	-1.97	-3.93	2.77.E-10
pud-3	176.43	-2.42	-5.37	2.46.E-09
pud-4	126.17	-2.02	-4.06	8.08.E-06
qua-1	15.35	-1.73	-3.32	2.07.E-04
R03G8.6	6.80	-1.16	-2.23	1.20.E-04
R04D3.2	55.87	-1.07	-2.10	2.65.E-05
R04D3.3	97.17	-1.03	-2.04	9.75.E-05
R05G6.9	0.97	-6.42	-85.83	7.98.E-03
R06C1.4	180.22	-1.06	-2.08	2.55.E-03
R07E5.11	44.19	-1.02	-2.03	1.59.E-04
R08E3.1	23.23	-1.14	-2.21	2.09.E-09
R08E5.3	23.86	-2.07	-4.20	4.04.E-09
R09A8.1	3.62	-1.47	-2.78	3.17.E-04
R09A8.2	9.88	-1.67	-3.18	1.26.E-08
R09F10.8	73.68	-1.02	-2.03	2.24.E-06
R09H10.3	44.57	-1.61	-3.05	2.66.E-12
R13D7.2	5.44	-1.44	-2.72	1.60.E-03
rbm-3.1	532.59	-1.03	-2.05	9.63.E-05
rgs-9	36.40	-1.70	-3.25	4.04.E-09
rhr-1	74.21	-1.54	-2.91	1.35.E-15
rol-1	8.47	-7.87	-234.28	1.89.E-08
rol-6	46.57	-6.25	-76.23	1.11.E-15

rol-8	78.72	-7.18	-145.17	9.88.E-25
sams-1	90.42	-1.41	-2.67	3.64.E-06
sams-3	64.85	-1.59	-3.01	2.73.E-12
scav-4	5.48	-1.39	-2.62	1.35.E-04
scav-5	6.39	-1.42	-2.68	1.02.E-05
sdz-18	3.14	-1.79	-3.46	3.32.E-03
sdz-24	18.39	-2.29	-4.90	1.74.E-06
sdz-28	16.75	-1.24	-2.36	1.06.E-03
sdz-30	13.32	-1.39	-2.62	1.75.E-03
sdz-33	5.02	-1.45	-2.73	1.05.E-03
sdz-4	7.98	-1.42	-2.67	1.45.E-03
sdz-9	5.32	-1.47	-2.77	7.02.E-04
sfxn-1.5	28.96	-1.00	-2.00	1.30.E-05
skr-10	44.37	-1.20	-2.29	6.24.E-05
skr-12	31.56	-1.34	-2.53	4.18.E-04
skr-14	53.01	-1.06	-2.08	8.71.E-04
skr-8	58.31	-1.05	-2.08	1.37.E-04
slc-17.3	2.03	-1.68	-3.20	2.57.E-04
smf-3	2.04	-1.73	-3.31	5.75.E-04
spch-1	4.51	-2.23	-4.71	1.64.E-03
spd-3	6.64	-1.73	-3.32	4.14.E-06
spds-1	10.15	-1.25	-2.38	1.17.E-04
spp-17	814.38	-1.17	-2.25	1.20.E-04
spp-4	25.13	-1.70	-3.24	2.17.E-07
spr-3	10.64	-1.15	-2.21	7.75.E-05
sqrd-1	12.47	-1.65	-3.14	2.59.E-08
sqt-1	55.96	-7.95	-247.79	6.23.E-19
sqt-2	78.65	-6.16	-71.29	7.35.E-21
srh-237	2.61	-2.28	-4.85	3.03.E-04
srv-7	1.46	-2.26	-4.80	2.94.E-03
ssq-1	4.58	-1.93	-3.82	1.96.E-03
ssq-2	2.61	-1.94	-3.83	8.13.E-03
sur-5	48.18	-1.21	-2.31	1.15.E-10
suro-1	11.33	-1.47	-2.77	9.80.E-04
T01G5.7	12.11	-1.07	-2.10	7.32.E-04
T01H8.2	7.86	-1.28	-2.43	9.59.E-04
T02G6.5	11.45	-1.15	-2.22	7.80.E-04
T03G11.6	31.90	-1.12	-2.18	1.19.E-06
T04D3.1	17.27	-1.03	-2.05	1.75.E-03
T05C3.6	7.02	-1.93	-3.80	3.91.E-08

T05E12.6	21.77	-3.50	-11.34	7.40.E-35
T05G5.4	2.49	-2.25	-4.76	5.87.E-03
T05H10.4	65.07	-1.08	-2.12	1.69.E-06
T06E4.12	4.29	-3.78	-13.76	1.42.E-05
T06G6.6	5.91	-2.86	-7.24	6.75.E-04
T09B9.1	24.71	-1.30	-2.47	4.04.E-06
T11F8.1	19.37	-1.38	-2.61	7.67.E-05
T12A2.3	9.37	-1.10	-2.14	7.36.E-03
T15B7.1	38.95	-3.51	-11.43	2.90.E-39
T16G12.1	5.98	-1.52	-2.86	5.96.E-08
T19H12.3	13.14	-1.22	-2.32	9.45.E-03
T19H5.7	5.01	-1.91	-3.75	8.20.E-03
T22B2.1	3.75	-1.67	-3.19	2.56.E-06
T24B8.5	223.07	-3.92	-15.15	8.11.E-45
T24D5.6	4.42	-1.38	-2.59	2.78.E-03
T24E12.1	15.57	-1.31	-2.49	3.33.E-04
T24E12.11	7.62	-1.02	-2.03	7.20.E-03
T25C12.3	46.39	-1.48	-2.78	4.67.E-16
T26E3.8	4.62	-1.64	-3.12	1.08.E-03
T27A8.2	10.97	-2.37	-5.17	1.32.E-06
T28D6.3	22.71	-2.69	-6.47	3.04.E-10
T28F3.8	15.27	-1.56	-2.95	1.19.E-09
tba-8	3.55	-3.32	-9.98	3.34.E-06
tbx-32	0.48	-3.61	-12.21	9.52.E-03
tig-3	1.03	-3.10	-8.57	1.10.E-03
tmem-135	12.04	-1.02	-2.03	2.88.E-04
tsp-10	10.70	-2.61	-6.09	7.03.E-11
ugt-17	8.27	-1.63	-3.10	9.01.E-06
ugt-22	23.31	-1.53	-2.88	1.10.E-10
ugt-30	1.29	-2.27	-4.83	7.45.E-05
ugt-43	4.37	-1.04	-2.06	2.29.E-03
ugt-44	49.33	-1.11	-2.15	3.59.E-09
ugt-47	14.35	-1.97	-3.92	7.56.E-12
ugt-63	6.25	-1.53	-2.89	1.02.E-04
vap-1	6.02	-2.42	-5.34	1.32.E-07
vet-1	24.24	-1.11	-2.15	3.79.E-05
vet-2	19.32	-1.01	-2.01	8.27.E-05
vet-6	15.18	-1.43	-2.70	1.78.E-05
vit-1	302.72	-1.65	-3.14	1.14.E-06
vit-2	4145.29	-1.46	-2.75	2.26.E-08

vit-3	1102.80	-1.67	-3.17	4.22.E-06
vit-4	1706.20	-2.64	-6.22	1.39.E-13
vit-5	3328.53	-1.84	-3.59	3.69.E-09
vit-6	4057.78	-1.41	-2.67	3.85.E-08
vrp-1	74.97	-1.38	-2.59	5.61.E-14
W02D7.4	8.91	-3.95	-15.40	3.73.E-14
W04A8.5	7.65	-1.01	-2.02	8.16.E-03
wago-11	0.84	-1.51	-2.85	6.95.E-03
Y105C5B.15	64.70	-1.64	-3.13	7.06.E-21
Y105C5B.5	108.24	-1.11	-2.15	1.15.E-05
Y106G6D.1	9.98	-1.17	-2.25	2.34.E-03
Y111B2A.21	2.14	-1.77	-3.40	7.49.E-03
Y113G7C.1	0.31	-2.38	-5.19	7.57.E-03
Y116A8C.19	5.33	-1.58	-3.00	4.71.E-03
Y116A8C.33	0.74	-1.82	-3.54	8.40.E-03
Y16B4A.2	11.14	-2.11	-4.31	9.89.E-25
Y17D7C.6	2.88	-2.58	-5.98	3.73.E-03
Y17G9A.4	8.05	-1.09	-2.12	6.91.E-03
Y18D10A.11	169.98	-1.06	-2.09	1.63.E-06
Y22D7AL.10	343.49	-1.23	-2.34	3.16.E-08
Y34B4A.9	121.31	-1.23	-2.35	3.69.E-11
Y37F4.8	2.05	-2.63	-6.18	2.82.E-03
Y39A1A.9	5.33	-1.75	-3.36	4.07.E-04
Y39B6A.27	2.11	-2.15	-4.44	7.38.E-04
Y39G8B.9	21.72	-2.08	-4.23	3.39.E-04
Y40H7A.10	45.66	-1.55	-2.92	1.43.E-16
Y43F11A.4	4.33	-1.46	-2.75	7.15.E-03
Y43F8B.24	6.04	-1.56	-2.95	3.05.E-03
Y44A6C.2	4.59	-1.27	-2.41	2.74.E-03
Y46G5A.28	4.44	-1.28	-2.44	3.46.E-03
Y46G5A.34	6.85	-1.91	-3.75	3.47.E-04
Y46H3C.5	81.79	-1.30	-2.47	2.96.E-05
Y46H3C.7	32.70	-1.32	-2.49	7.89.E-06
Y47D7A.15	6.36	-5.68	-51.19	1.76.E-09
Y47G6A.15	9.52	-2.70	-6.50	5.30.E-05
Y47H10A.2	2.35	-1.49	-2.82	3.08.E-03
Y48E1B.8	7.99	-6.73	-106.29	1.10.E-09
Y48G8AL.12	61.39	-4.64	-24.97	7.31.E-10
Y48G8AL.15	12.09	-1.17	-2.26	6.54.E-03
Y51F10.7	179.41	-2.73	-6.64	7.34.E-51

Y51H4A.5	2.72	-2.39	-5.23	2.86.E-05
Y51H7C.13	5.38	-1.49	-2.82	2.86.E-03
Y51H7C.15	7.78	-1.26	-2.40	6.89.E-03
Y52B11B.1	4.13	-6.39	-84.08	8.69.E-03
Y54F10AM.8	41.62	-1.42	-2.67	4.64.E-12
Y54G2A.45	26.39	-2.31	-4.97	1.92.E-20
Y54G2A.49	10.79	-1.56	-2.95	6.62.E-05
Y57A10A.3	8.76	-1.26	-2.39	1.35.E-03
Y57A10A.7	3.97	-1.52	-2.87	3.50.E-05
Y64H9A.2	5.89	-2.11	-4.31	2.05.E-03
Y71A12B.11	25.73	-1.42	-2.68	1.24.E-03
Y73C8C.3	1.70	-1.28	-2.43	5.38.E-03
Y73C8C.8	3.55	-1.25	-2.38	2.10.E-03
Y75D11A.3	17.24	-1.31	-2.48	2.74.E-04
Y7A5A.1	30.74	-1.50	-2.84	4.72.E-15
Y82E9BR.17	19.92	-1.02	-2.02	8.77.E-03
Y82E9BR.19	20.08	-1.15	-2.22	2.88.E-07
Y8A9A.2	1.57	-1.73	-3.31	1.41.E-05
Y9C9A.13	1.02	-1.79	-3.45	3.48.E-03
ZC116.3	1.22	-1.31	-2.47	4.65.E-05
ZC266.1	4.02	-1.75	-3.36	1.36.E-04
ZC376.3	4.35	-1.73	-3.31	7.47.E-07
ZC443.1	3.77	-1.51	-2.86	8.76.E-04
ZC53.1	9.49	-1.02	-2.03	1.23.E-03
zip-8	33.51	-1.03	-2.04	1.27.E-03
ZK1025.2	8.67	-1.62	-3.08	7.71.E-03
ZK1025.3	25.11	-1.73	-3.32	7.40.E-03
ZK1025.8	8.01	-1.77	-3.41	9.04.E-03
ZK1307.1	84.91	-2.13	-4.39	1.34.E-21
ZK180.6	18.82	-3.74	-13.40	3.90.E-10
ZK185.3	7.25	-1.12	-2.18	4.05.E-03
ZK550.2	14.52	-1.29	-2.44	1.62.E-06
ZK6.6	2.58	-1.77	-3.42	2.15.E-04
ZK666.1	2.08	-4.02	-16.21	4.67.E-03
ZK822.4	31.18	-1.48	-2.80	2.34.E-07
ZK899.5	4.09	-1.40	-2.64	4.12.E-03
zmp-2	3.85	-1.49	-2.80	1.88.E-03

Supplementary Table5

RNA sequencing analysis of animals cultivated at 25°C for 20 hours under CPT exposure

(logFC>1 p<0.01)

Name	Max group means	Fold change	Log fold change	P-value
F55G11.2	76.95	87.03	6.44	6.46E-03
clec-174	67.76	26.58	4.73	4.64E-04
gst-5	172.90	21.75	4.44	4.38E-06
cld-9	38.68	18.53	4.21	8.71E-04
nspc-1	159.59	17.65	4.14	1.80E-03
F49F1.7	194.73	14.69	3.88	7.50E-08
dod-21	200.67	14.14	3.82	1.05E-08
Y34F4.5	62.45	13.97	3.80	2.95E-03
F33H12.7	126.89	12.51	3.64	2.47E-04
K08D8.4	22.04	12.45	3.64	5.27E-03
irg-5	92.92	12.20	3.61	2.09E-05
K11H12.4	31.07	11.71	3.55	8.42E-03
C33G8.2	28.61	11.70	3.55	8.16E-03
C17H12.6	80.94	10.34	3.37	1.11E-04
ges-1	30.61	8.97	3.17	1.80E-03
Y47H10A.5	131.80	8.75	3.13	4.94E-07
Y22D7AL.15	93.88	8.53	3.09	2.24E-03
C49G7.10	53.50	8.53	3.09	2.14E-03
skr-3	101.66	8.49	3.09	2.32E-04
ZK899.2	48.85	8.18	3.03	3.28E-04
thn-2	180.26	7.74	2.95	7.01E-07
dod-22	120.87	7.56	2.92	4.15E-06
K08D8.5	332.64	6.63	2.73	1.22E-14
hpo-6	113.48	6.40	2.68	5.75E-08
Y37H2A.14	52.64	6.33	2.66	9.51E-03
F35E12.6	331.91	6.18	2.63	4.30E-14
cebp-1	68.40	6.11	2.61	1.35E-04
F08G2.5	64.54	6.01	2.59	2.88E-03
F53B2.8	153.92	5.92	2.57	5.46E-06
spp-4	104.80	5.70	2.51	3.69E-03
Y46D2A.2	33.76	5.39	2.43	4.91E-03
dod-17	92.66	5.30	2.41	1.44E-05
T01D3.6	89.86	5.11	2.35	2.78E-10
oac-6	54.37	5.08	2.35	4.99E-05

cdr-4	49.09	5.07	2.34	7.73E-03
fbxa-60	91.93	5.05	2.34	2.73E-05
F55B11.4	145.98	5.00	2.32	1.21E-03
swt-6	157.03	4.98	2.32	4.05E-06
ctl-3	33.63	4.93	2.30	9.81E-03
pacs-1	36.05	4.84	2.28	6.94E-03
F49C12.7	49.77	4.82	2.27	3.75E-04
F01D5.1	67.81	4.77	2.25	9.84E-03
C34H4.2	105.24	4.73	2.24	5.44E-07
clec-67	94.74	4.69	2.23	4.18E-08
C29F7.2	38.73	4.64	2.21	4.49E-03
alh-11	22.95	4.63	2.21	3.89E-03
F35E12.10	69.26	4.59	2.20	2.35E-04
clec-186	252.95	4.50	2.17	4.47E-10
cpr-3	61.22	4.45	2.15	6.07E-04
F57B9.1	54.35	4.36	2.12	2.42E-03
K08D8.6	108.35	4.35	2.12	3.00E-06
B0024.4	207.72	4.32	2.11	1.45E-08
clec-62	80.47	4.26	2.09	5.25E-04
cpr-4	702.00	4.20	2.07	0
B0495.5	24.59	4.16	2.06	3.43E-03
spp-18	86.75	4.16	2.06	7.95E-03
irg-4	100.36	4.12	2.04	4.20E-04
drd-50	196.69	4.07	2.02	2.16E-07
clc-1	290.11	4.05	2.02	7.72E-09
F54E2.1	376.65	3.98	1.99	1.19E-12
mtl-2	153.82	3.97	1.99	4.65E-03
C18H9.6	106.11	3.92	1.97	1.44E-03
ZK1055.7	28.74	3.77	1.92	6.99E-03
F54C9.3	358.70	3.71	1.89	2.81E-05
spp-8	110.09	3.68	1.88	2.61E-05
C32H11.4	184.09	3.65	1.87	7.85E-06
W05H9.1	421.79	3.59	1.84	4.73E-14
clec-83	166.91	3.50	1.81	5.20E-06
nlp-36	203.28	3.40	1.76	5.82E-03
T27F6.7	56.86	3.35	1.74	8.09E-03
F52B11.2	45.71	3.35	1.74	8.51E-03
tag-120	29.84	3.32	1.73	7.79E-03

nspc-15	193.09	3.28	1.71	4.42E-03
F28B4.3	34.77	3.26	1.70	1.69E-06
dod-24	375.20	3.22	1.69	1.75E-10
C49C3.6	71.69	3.18	1.67	8.43E-03
clec-209	1301.14	3.15	1.66	0
ugt-44	124.48	3.14	1.65	1.05E-05
C17H12.8	769.79	3.13	1.65	8.12E-14
lys-8	1026.03	3.06	1.61	1.11E-16
MTCE.7	342.25	2.99	1.58	2.79E-06
T10E9.2	36.67	2.98	1.58	2.35E-03
dhs-3	158.12	2.98	1.57	7.92E-05
ZK6.11	884.25	2.95	1.56	1.76E-13
ttr-46	472.45	2.93	1.55	8.85E-07
F56C9.7	385.23	2.91	1.54	4.91E-08
T21F4.1	83.01	2.91	1.54	1.62E-03
dod-23	547.88	2.91	1.54	8.29E-08
ZK1320.3	585.50	2.88	1.53	6.82E-10
faah-2	54.03	2.87	1.52	9.91E-04
F56A4.2	1186.65	2.87	1.52	2.39E-14
npa-1	122.12	2.85	1.51	1.11E-08
F46F11.8	87.77	2.84	1.50	4.52E-04
C12D12.1	58.08	2.83	1.50	1.42E-04
clec-85	345.33	2.81	1.49	3.52E-08
T02C5.1	186.06	2.80	1.49	3.72E-06
C26B9.5	79.58	2.77	1.47	1.07E-03
AC3.5	20.27	2.76	1.47	8.31E-03
F19C7.1	287.54	2.75	1.46	4.59E-07
ugt-62	78.99	2.75	1.46	1.56E-03
C05D2.8	109.04	2.71	1.44	1.56E-04
Y11D7A.7	133.33	2.71	1.44	8.61E-04
tdo-2	46.69	2.70	1.43	6.06E-03
ttr-48	275.09	2.70	1.43	4.72E-05
F54D5.4	1020.47	2.66	1.41	5.43E-11
C24B9.3	51.52	2.65	1.41	4.82E-03
C18B2.4	97.84	2.65	1.40	1.31E-03
K12H4.7	501.79	2.64	1.40	1.07E-12
F56F10.1	63.93	2.63	1.39	5.82E-03
F20D1.3	135.42	2.62	1.39	1.19E-04

vem-1	91.56	2.61	1.38	7.28E-03
M28.10	175.57	2.57	1.36	1.17E-04
pfn-2	180.41	2.57	1.36	2.42E-04
alh-12	46.44	2.57	1.36	7.84E-03
mes-3	55.38	2.56	1.35	1.01E-04
ggtb-1	37.41	2.53	1.34	4.60E-03
smut-1	66.77	2.53	1.34	2.09E-03
nlt-1	203.72	2.52	1.33	5.01E-03
sdz-27	144.21	2.52	1.33	2.24E-03
C16B8.3	159.71	2.48	1.31	3.64E-03
Y37E11AM.2	72.03	2.46	1.30	7.64E-03
Y34B4A.9	312.17	2.46	1.30	9.12E-06
M02H5.8	232.80	2.45	1.29	8.76E-03
F19F10.11	25.61	2.41	1.27	4.99E-03
F09E5.7	65.70	2.39	1.26	4.69E-03
ZK1320.2	269.09	2.38	1.25	1.84E-04
Y119D3B.21	998.61	2.38	1.25	2.72E-05
col-80	429.32	2.38	1.25	4.54E-06
rog-1	44.59	2.38	1.25	4.05E-03
F20D1.1	142.34	2.37	1.25	3.73E-03
F11G11.5	153.07	2.37	1.24	6.36E-04
ugt-22	55.99	2.35	1.23	4.77E-03
F58D5.5	36.05	2.31	1.21	9.21E-03
T12B3.3	134.44	2.31	1.21	5.03E-04
R07E3.1	88.10	2.31	1.21	6.42E-03
lec-8	351.52	2.30	1.20	5.51E-07
W05H9.3	282.59	2.29	1.19	3.98E-06
K06G5.1	478.76	2.27	1.19	1.22E-08
Y34B4A.6	538.07	2.27	1.18	3.78E-07
csn-5	77.67	2.26	1.18	7.80E-03
F59B1.2	327.50	2.26	1.17	3.68E-04
tth-1	226.95	2.22	1.15	9.92E-04
lron-7	126.24	2.22	1.15	1.33E-03
T08H10.1	111.51	2.21	1.15	4.74E-03
T20D3.2	576.51	2.20	1.14	7.79E-07
col-8	310.04	2.18	1.13	1.80E-04
F46G10.1	148.41	2.18	1.13	6.16E-03
F55B11.3	116.31	2.17	1.12	4.34E-03

cpr-6	1448.35	2.17	1.12	1.21E-11
LLC1.2	289.08	2.17	1.12	2.14E-05
asp-2	453.66	2.14	1.10	5.38E-07
dvc-1	136.91	2.14	1.10	5.05E-04
F28H7.3	181.13	2.14	1.10	6.32E-04
pfk-1.1	52.53	2.14	1.10	5.07E-04
T23G11.7	59.27	2.12	1.08	3.90E-03
H34I24.2	106.41	2.11	1.08	1.47E-03
F13D12.6	484.04	2.11	1.08	2.42E-08
rict-1	37.87	2.11	1.08	5.64E-04
lec-6	1106.12	2.11	1.08	5.50E-07
sac-1	130.03	2.10	1.07	7.89E-04
col-160	254.65	2.06	1.04	3.88E-04
F55G11.4	313.24	2.06	1.04	7.72E-05
Y32F6A.5	226.44	2.05	1.03	7.05E-06
C49C3.9	87.11	2.04	1.03	4.84E-03
ttr-41	274.45	2.04	1.03	4.53E-03
sdhd-1	225.12	2.04	1.03	7.05E-03

Supplementary Table6

RNA sequencing analysis of animals cultivated at 25°C for 20 hours under CPT exposure (logFC<-1 p<0.01)

Name	Max group means	Fold change	Log fold change	P-value
pod-2	84.69	-2.16	-1.11	9.30E-08
nol-56	201.16	-2.18	-1.13	9.83E-06
unc-54	178.69	-2.23	-1.16	4.10E-06
his-24	322.09	-2.54	-1.34	1.01E-05
cav-1	406.27	-2.55	-1.35	4.19E-07
vab-10	9.46	-2.84	-1.51	8.10E-06
mig-6	34.26	-2.93	-1.55	1.00E-05
icl-1	202.29	-2.98	-1.57	4.83E-10
T24B8.3	247.41	-3.05	-1.61	2.36E-06
clu-1	55.37	-3.14	-1.65	3.74E-07
hil-2	191.50	-3.29	-1.72	5.46E-05
fasn-1	30.60	-3.31	-1.73	1.52E-07
anc-1	10.03	-3.34	-1.74	6.95E-08
unc-52	10.53	-3.35	-1.74	2.85E-05
unc-22	5.26	-3.66	-1.87	7.25E-05
noah-1	33.93	-3.81	-1.93	3.94E-05
col-3	800.44	-4.10	-2.04	3.99E-09
col-144	511.77	-4.93	-2.30	6.08E-09
sqt-3	280.52	-5.19	-2.38	5.47E-10
epg-2	76.89	-5.20	-2.38	2.11E-07
sodh-1	132.11	-5.53	-2.47	9.80E-07
col-73	263.80	-6.21	-2.63	7.31E-07
acdh-1	121.67	-6.23	-2.64	4.59E-06
dpy-13	258.93	-6.66	-2.74	4.23E-06
F41F3.3	512.22	-7.04	-2.82	2.69E-10
ZK180.5	104.70	-8.85	-3.15	5.55E-08
epi-1	14.01	-9.05	-3.18	1.83E-07
dpy-14	110.23	-9.69	-3.28	8.28E-06
col-107	138.25	-10.96	-3.45	2.18E-05
ram-2	150.52	-11.16	-3.48	8.52E-07
col-125	285.77	-12.07	-3.59	1.02E-07
cut-2	270.41	-13.92	-3.80	2.75E-10
F53F1.4	286.29	-16.63	-4.06	3.05E-06
Y47D7A.13	134.66	-5.60	-2.49	1.13E-04
col-167	304.57	-3.72	-1.90	1.49E-04

ssl-1	22.34	-2.58	-1.37	1.72E-04
col-39	78.28	-12.64	-3.66	2.19E-04
sma-1	13.88	-2.32	-1.21	2.51E-04
ztf-6	40.47	-5.75	-2.52	2.85E-04
dpy-4	283.60	-4.59	-2.20	2.92E-04
cht-1	85.02	-2.50	-1.32	3.11E-04
col-166	290.44	-2.75	-1.46	3.27E-04
clec-266	113.23	-3.17	-1.66	3.99E-04
C35E7.5	28.46	-3.15	-1.66	5.27E-04
pyr-1	19.55	-2.58	-1.37	5.57E-04
col-10	341.04	-3.04	-1.60	6.93E-04
pat-12	15.10	-2.94	-1.56	7.42E-04
col-94	105.84	-6.24	-2.64	8.32E-04
unc-44	5.89	-2.58	-1.37	9.24E-04
T05E11.9	119.74	-2.38	-1.25	1.05E-03
R166.6	46.13	-22.78	-4.51	1.09E-03
chd-7	15.54	-2.34	-1.23	1.13E-03
col-13	71.83	-10.70	-3.42	1.16E-03
mca-1	36.27	-2.13	-1.09	1.20E-03
algn-6	42.78	-5.54	-2.47	1.24E-03
ppw-2	21.03	-4.69	-2.23	1.24E-03
wars-1	77.48	-2.46	-1.30	1.48E-03
C17E4.6	88.19	-2.29	-1.19	1.65E-03
meg-4	24.15	-4.51	-2.17	1.70E-03
col-41	81.89	-4.51	-2.17	1.90E-03
pud-1.1	290.11	-7.45	-2.90	2.00E-03
rol-8	67.59	-6.56	-2.71	2.01E-03
mlt-10	23.65	-14.89	-3.90	2.21E-03
slc-25A46	41.86	-3.82	-1.93	2.31E-03
B0019.2	29.79	-4.10	-2.04	2.34E-03
gfi-1	66.38	-2.38	-1.25	2.39E-03
col-92	69.58	-10.57	-3.40	2.48E-03
egl-30	63.78	-2.05	-1.04	2.53E-03
let-805	5.55	-3.46	-1.79	2.80E-03
oxa-1	44.99	-3.55	-1.83	2.82E-03
Y4C6B.1	69.32	-2.41	-1.27	2.88E-03
gcy-28	18.77	-2.34	-1.22	2.88E-03
C09F9.2	10.09	-8.20	-3.04	2.91E-03

dpy-17	61.18	-3.93	-1.97	3.05E-03
F13C5.2	32.02	-5.77	-2.53	3.09E-03
ifa-4	26.93	-4.67	-2.22	3.61E-03
Y48G8AL.5	35.82	-2.89	-1.53	3.63E-03
col-14	46.83	-13.49	-3.75	3.85E-03
hpo-29	36.80	-2.29	-1.19	4.02E-03
aqp-2	108.28	-2.10	-1.07	4.20E-03
lgc-22	33.97	-5.49	-2.46	4.26E-03
mics-1	91.98	-2.22	-1.15	4.28E-03
lin-9	36.54	-2.72	-1.44	4.44E-03
vet-2	23.26	-3.98	-1.99	4.46E-03
mrps-12	82.74	-12.10	-3.60	5.00E-03
lrp-1	4.77	-3.12	-1.64	5.09E-03
sams-4	65.85	-2.14	-1.10	5.33E-03
cbs-1	20.75	-7.42	-2.89	5.58E-03
mrpl-38	54.93	-2.89	-1.53	5.59E-03
tni-1	53.37	-3.80	-1.93	5.89E-03
rbg-2	17.83	-2.89	-1.53	5.95E-03
C17H11.6	13.89	-7.00	-2.81	6.03E-03
grl-16	56.70	-93.57	-6.55	6.03E-03
R06C1.4	170.38	-2.63	-1.40	6.06E-03
T13H5.8	61.58	-2.13	-1.09	6.17E-03
dpy-21	16.49	-2.35	-1.23	6.86E-03
col-81	48.12	-14.21	-3.83	6.87E-03
C02E7.6	128.28	-11.40	-3.51	6.90E-03
T04F3.1	3.93	-6.59	-2.72	6.91E-03
hsf-1	45.41	-2.22	-1.15	6.92E-03
col-17	220.96	-3.92	-1.97	7.02E-03
tep-1	16.11	-2.63	-1.40	7.04E-03
C06A5.6	27.10	-2.50	-1.32	7.31E-03
lin-26	57.29	-2.24	-1.16	7.43E-03
fzr-1	20.03	-3.50	-1.81	7.45E-03
cogc-3	32.41	-2.35	-1.23	7.66E-03
C02F12.5	64.55	-4.92	-2.30	7.82E-03
T10F2.2	58.70	-2.89	-1.53	8.07E-03
col-129	43.01	-13.48	-3.75	8.17E-03
nol-10	43.05	-2.15	-1.10	8.45E-03
daf-4	16.40	-3.62	-1.86	8.61E-03

mlt-8	41.97	-4.92	-2.30	9.07E-03
cogc-4	38.72	-2.16	-1.11	9.15E-03
pop-1	47.69	-2.61	-1.39	9.34E-03
col-154	43.85	-7.68	-2.94	9.66E-03
fce-2	33.85	-10.71	-3.42	9.69E-03
sams-1	60.87	-2.23	-1.16	9.76E-03

Supplementary Table7

RNA sequencing analysis of animals exposed to CPT at 2°C for 3 hr.

(LogFC>1, p<0.01)

Name	Max group mean	Log_2 fold change	Fold change	P-value
acd-3	2.06	1.45	2.72	6.57.E-03
acox-1.3	6.43	1.12	2.17	2.31.E-03
acox-1.4	25.04	1.33	2.51	1.48.E-06
alh-5	11.88	1.19	2.28	1.57.E-04
amt-1	14.47	1.29	2.44	1.15.E-06
anmt-2	18.77	1.11	2.16	4.68.E-04
arrd-7	12.41	1.03	2.05	1.16.E-03
asah-1	62.37	1.30	2.47	2.17.E-08
asah-2	60.10	1.19	2.28	4.15.E-10
asm-2	14.47	2.24	4.72	1.04.E-11
asm-3	6.81	1.31	2.49	3.14.E-03
asp-12	16.11	1.63	3.09	6.51.E-08
asp-14	94.09	1.48	2.79	5.56.E-13
asp-2	392.37	1.08	2.12	1.52.E-08
asp-3	796.90	1.09	2.12	2.46.E-07
B0024.13	74.24	1.09	2.12	2.44.E-09
B0024.4	177.48	2.28	4.86	4.79.E-09
B0252.1	1.57	1.32	2.49	3.17.E-03
B0281.4	3.21	2.00	3.99	8.92.E-04
B0304.4	50.85	1.25	2.37	1.48.E-07
B0334.6	2.37	1.44	2.72	9.09.E-03
B0393.9	9.64	1.16	2.23	2.76.E-03
B0403.3	10.51	1.04	2.06	8.03.E-04
B0462.5	12.51	2.59	6.03	1.17.E-08
bath-24	10.16	1.07	2.10	2.41.E-03
bath-26	5.58	1.53	2.90	3.22.E-04
bath-36	5.85	1.23	2.35	1.62.E-03
bath-4	16.91	1.03	2.05	7.28.E-04
bath-47	5.95	1.24	2.36	1.45.E-04
bcmo-2	18.77	2.07	4.19	3.98.E-16
bgal-2	14.80	1.11	2.16	2.14.E-05
btb-9	3.54	1.34	2.52	7.34.E-03
C01B10.3	43.97	1.22	2.34	1.52.E-08
C03B1.14	14.62	1.15	2.22	4.52.E-04
C04F12.5	56.16	1.36	2.57	3.33.E-07
C04G6.5	16.82	2.24	4.71	1.38.E-05

C05C12.4	45.48	1.14	2.21	1.92.E-05
C05D2.8	86.01	1.33	2.51	1.75.E-11
C06B8.2	9.15	1.66	3.16	2.46.E-06
C06E4.8	5.19	1.63	3.09	1.35.E-03
C06G4.4	5.08	2.23	4.68	3.48.E-04
C07E3.3	7.50	1.12	2.18	7.78.E-04
C07F11.2	3.36	2.09	4.25	9.65.E-06
C08E8.10	10.12	1.46	2.75	6.59.E-04
C08E8.4	49.35	2.13	4.38	1.19.E-19
C10C5.2	17.18	2.38	5.19	1.02.E-11
C12D12.1	60.12	1.01	2.01	2.51.E-07
C14C6.2	55.00	1.03	2.04	6.77.E-04
C15B12.1	6.83	1.03	2.05	5.88.E-03
C15F1.5	5.37	1.19	2.28	1.46.E-03
C16C10.9	1.31	2.59	6.02	5.08.E-03
C16C8.13	7.02	1.31	2.49	3.41.E-03
C16C8.14	24.19	1.50	2.83	5.93.E-07
C16C8.21	1.89	2.77	6.83	5.45.E-03
C16C8.4	63.52	1.05	2.07	6.61.E-07
C16D9.4	11.18	1.28	2.43	1.20.E-04
C17B7.10	2.56	1.86	3.62	1.14.E-03
C17B7.12	7.43	4.72	26.37	1.70.E-07
C17B7.15	4.93	2.64	6.24	1.59.E-05
C17B7.3	2.89	1.91	3.75	2.13.E-03
C17B7.4	8.40	1.38	2.60	2.86.E-03
C17B7.5	0.79	1.86	3.64	1.89.E-03
C17C3.3	1.02	2.67	6.34	2.64.E-03
C17G1.2	79.32	1.01	2.02	1.51.E-07
C17H12.4	21.88	1.32	2.50	6.56.E-09
C17H12.6	51.47	3.26	9.56	2.37.E-28
C17H12.8	523.07	1.74	3.33	3.45.E-17
C18A11.1	32.59	1.63	3.10	5.27.E-08
C18H7.11	26.62	2.79	6.91	1.32.E-16
C18H9.6	66.53	1.82	3.53	2.88.E-14
C23G10.1	1.61	2.15	4.43	2.24.E-03
C24B9.3	95.91	1.01	2.02	1.71.E-08
C25D7.5	10.23	1.76	3.38	8.09.E-07
C25E10.8	38.30	1.17	2.26	1.09.E-04
C25F9.11	28.10	1.66	3.16	3.89.E-08
C25F9.12	6.71	1.42	2.67	3.46.E-03

C26B9.5	57.36	1.19	2.28	2.68.E-11
C26E1.2	4.92	1.20	2.29	1.06.E-03
C27H5.2	9.01	1.02	2.03	3.88.E-04
C27H5.4	17.56	1.70	3.26	5.39.E-09
C28C12.11	1.99	1.63	3.10	9.14.E-03
C28G1.5	22.99	1.21	2.31	1.94.E-05
C28G1.6	8.08	1.28	2.43	1.30.E-03
C29F3.7	58.95	1.32	2.50	1.42.E-09
C29F7.1	7.74	1.61	3.06	8.10.E-06
C29F7.2	54.62	1.65	3.14	6.14.E-10
C29F9.2	13.44	1.12	2.18	2.25.E-03
C29G2.6	36.14	1.25	2.38	4.19.E-06
C30G12.2	17.09	1.98	3.95	1.03.E-04
C31G12.4	1.13	2.20	4.61	1.33.E-04
C32H11.3	3.04	1.90	3.72	5.54.E-04
C32H11.4	79.88	2.35	5.08	3.75.E-17
C32H11.9	73.38	1.99	3.98	4.22.E-06
C33H5.13	54.78	1.41	2.65	6.53.E-08
C34B7.1	4.30	1.10	2.15	7.30.E-03
C34H4.1	24.08	1.15	2.23	1.89.E-04
C34H4.2	105.61	1.91	3.77	1.78.E-26
C36C5.5	33.16	1.40	2.64	7.20.E-05
C37C3.10	6.07	2.73	6.62	2.26.E-05
C39B5.14	1.38	2.46	5.49	2.47.E-03
C39F7.5	14.39	2.20	4.59	1.77.E-14
C40H1.8	9.05	2.92	7.56	8.15.E-11
C41G7.8	22.33	2.07	4.19	4.83.E-07
C42C1.3	1.22	3.14	8.79	4.84.E-03
C42D4.19	12.71	3.75	13.41	2.28.E-07
C42D4.2	5.86	1.32	2.49	1.52.E-03
C42D8.1	23.79	1.60	3.03	5.39.E-06
C44C10.11	4.04	1.53	2.88	3.21.E-04
C44E12.1	13.93	1.03	2.04	1.83.E-03
C45B11.2	7.06	2.37	5.18	1.00.E-05
C45B2.1	86.18	1.76	3.38	2.64.E-10
C46F2.1	11.62	1.88	3.67	3.22.E-06
C47D12.5	31.43	1.26	2.40	1.12.E-05
C49A9.3	4.74	1.19	2.28	1.90.E-03
C49C3.11	1.90	3.41	10.65	1.82.E-04
C49C3.9	75.27	1.46	2.76	5.80.E-11

C49F5.6	30.98	1.04	2.06	2.17.E-06
C49G7.12	10.14	1.87	3.65	1.17.E-06
C50A2.3	9.45	1.24	2.36	1.11.E-03
C50B6.7	24.76	1.94	3.85	2.06.E-08
C50B8.4	4.68	1.84	3.57	1.59.E-03
C50F4.9	8.82	1.26	2.39	4.19.E-03
C52A10.1	26.56	1.44	2.71	2.15.E-07
C53B4.3	14.53	1.18	2.27	4.00.E-06
C54C6.7	14.11	1.68	3.21	2.65.E-04
C54E4.5	35.79	1.16	2.23	1.18.E-05
C55C3.3	6.23	2.11	4.31	2.78.E-08
cdd-1	8.45	1.58	2.99	1.44.E-05
cdr-1	2.62	2.05	4.13	2.27.E-03
cdr-2	66.25	1.44	2.71	7.47.E-07
cdr-4	49.55	1.26	2.40	2.07.E-10
cdr-7	11.37	1.36	2.57	3.22.E-04
cest-1	3.29	1.24	2.35	3.05.E-03
cgr-1	25.54	1.01	2.02	7.09.E-05
cgt-1	10.53	1.52	2.87	1.21.E-05
chil-13	8.71	1.04	2.06	1.98.E-03
chil-22	6.12	1.19	2.28	3.32.E-03
clc-1	226.93	1.80	3.48	3.76.E-21
cld-9	41.40	2.45	5.46	1.35.E-18
clec-125	0.65	1.93	3.81	5.20.E-03
clec-143	1.34	2.21	4.64	2.18.E-03
clec-160	32.69	1.35	2.55	7.40.E-08
clec-166	15.21	1.26	2.39	1.51.E-03
clec-167	1.21	1.87	3.65	7.89.E-03
clec-169	2.06	2.59	6.00	5.45.E-05
clec-173	63.36	1.83	3.56	4.78.E-10
clec-174	38.02	4.65	25.11	1.49.E-25
clec-186	155.23	1.28	2.43	2.43.E-11
clec-187	33.64	1.46	2.75	1.36.E-07
clec-209	836.23	2.13	4.39	2.30.E-30
clec-210	5.21	2.44	5.41	4.88.E-05
clec-245	1.71	2.65	6.26	5.10.E-03
clec-3	3.15	2.95	7.72	8.45.E-07
clec-45	5.18	2.48	5.57	1.78.E-03
clec-47	32.53	1.77	3.41	1.97.E-07
clec-5	38.68	1.77	3.40	1.87.E-13

clec-51	29.51	1.29	2.44	1.05.E-05
clec-54	8.24	1.03	2.04	6.21.E-03
clec-55	1.35	2.12	4.34	7.00.E-03
clec-56	18.39	1.02	2.03	1.09.E-03
clec-6	5.11	4.12	17.45	3.80.E-11
clec-60	2.07	1.89	3.72	1.96.E-04
clec-62	101.14	1.55	2.92	1.73.E-17
clec-67	89.73	2.08	4.23	9.53.E-16
clec-71	2.80	1.70	3.25	2.46.E-03
clec-8	4.61	1.22	2.32	2.90.E-03
clec-80	7.13	1.74	3.35	1.02.E-05
clec-83	141.45	1.28	2.43	1.36.E-11
clec-84	34.32	1.00	2.01	1.55.E-04
clec-85	423.42	1.34	2.53	2.25.E-12
clec-86	33.52	1.95	3.86	1.90.E-06
clp-6	1.23	1.37	2.58	9.40.E-03
col-135	22.83	2.88	7.37	1.21.E-13
col-45	1.48	3.42	10.67	2.40.E-04
col-96	33.76	2.77	6.84	2.27.E-13
comt-2	6.10	1.83	3.55	9.32.E-04
cpr-3	78.08	1.32	2.49	8.75.E-11
cpr-4	492.78	2.21	4.64	4.00.E-24
crn-6	12.63	1.07	2.09	6.39.E-04
csb-1	18.11	1.11	2.16	4.78.E-06
cyp-14A5	17.17	1.14	2.21	1.69.E-05
cyp-29A3	8.24	2.24	4.72	2.39.E-09
cyp-33C4	5.32	1.07	2.11	5.14.E-03
cyp-33C7	8.25	1.16	2.24	6.33.E-04
cyp-33C9	14.50	1.37	2.58	9.29.E-06
cyp-35A1	0.59	3.18	9.04	9.23.E-03
cyp-35A5	10.02	3.87	14.63	5.91.E-14
cyp-35B1	2.55	1.51	2.86	1.72.E-03
cyp-35B2	1.59	2.75	6.72	3.11.E-03
cyp-35C1	26.97	2.01	4.02	1.78.E-11
D1044.1	10.72	1.86	3.63	1.24.E-07
D1086.6	65.96	1.04	2.05	1.07.E-06
dct-17	37.28	1.46	2.75	6.63.E-16
decr-1.2	0.89	2.70	6.50	8.08.E-03
dhc-3	0.72	2.46	5.52	9.90.E-07
dhs-14	46.65	1.44	2.72	2.80.E-10

dhs-2	17.87	1.82	3.53	9.74.E-09
dhs-20	10.98	1.44	2.71	2.58.E-05
dhs-23	1.46	2.18	4.54	6.60.E-03
dhs-26	1.64	2.82	7.08	4.52.E-04
dhs-3	121.97	1.29	2.45	2.23.E-10
dhs-7	5.37	1.04	2.05	5.09.E-03
dod-17	56.64	2.22	4.65	1.96.E-12
dod-19	458.65	1.08	2.11	5.73.E-09
dod-20	1.97	3.31	9.93	1.83.E-05
dod-21	77.19	1.69	3.22	8.34.E-04
dod-22	72.58	1.98	3.93	1.04.E-13
dod-23	358.54	1.49	2.81	3.50.E-14
dod-24	138.48	1.31	2.47	1.07.E-10
dod-3	63.08	1.38	2.61	6.17.E-05
drd-50	99.07	1.85	3.61	5.51.E-16
E02C12.6	2.26	2.72	6.57	2.20.E-04
E02H4.7	18.93	1.73	3.31	5.31.E-05
E03H4.8	18.90	2.08	4.21	9.89.E-11
E04F6.15	11.48	1.47	2.77	4.20.E-05
egl-1	15.39	2.63	6.21	4.61.E-09
elt-4	3.56	3.25	9.50	8.79.E-04
ent-7	10.36	1.21	2.32	1.53.E-04
ets-9	7.01	1.11	2.16	6.03.E-03
F01D5.2	21.51	1.85	3.60	1.97.E-06
F01D5.3	26.39	1.04	2.06	1.91.E-03
F08D12.2	13.72	1.27	2.41	1.70.E-03
F08D12.3	29.72	1.17	2.25	1.86.E-05
F08F1.3	10.71	1.18	2.27	2.43.E-03
F08G2.8	9.39	3.31	9.92	1.22.E-10
F09A5.2	3.14	1.53	2.90	6.22.E-05
F10A3.17	36.77	1.72	3.29	4.08.E-11
F10A3.4	3.40	1.13	2.18	7.06.E-03
F10D2.10	6.98	1.16	2.23	2.46.E-03
F11D5.7	1.98	1.74	3.33	1.14.E-03
F11E6.6	3.17	1.56	2.94	2.23.E-03
F13H6.3	32.25	1.76	3.39	7.84.E-16
F15B9.6	7.00	2.70	6.52	5.87.E-13
F15E6.6	4.92	1.13	2.18	1.03.E-04
F16H6.10	15.15	1.09	2.12	2.40.E-04
F19C6.8	4.68	2.67	6.38	1.56.E-03

F19C7.1	255.77	1.16	2.23	2.69.E-08
F19C7.2	7.33	1.59	3.01	1.74.E-07
F19C7.4	6.66	1.95	3.87	7.54.E-06
F20D6.5	7.44	2.10	4.28	2.32.E-06
F20G2.1	2.85	2.09	4.26	3.30.E-03
F20G2.5	44.06	3.92	15.13	4.45.E-40
F22E5.1	13.63	1.49	2.81	3.23.E-06
F22H10.2	47.27	1.57	2.98	7.40.E-08
F23C8.8	3.91	2.38	5.19	4.01.E-05
F25B4.8	19.97	1.16	2.24	8.37.E-04
F25D1.5	2.54	1.82	3.53	2.57.E-03
F26C11.1	3.64	1.90	3.73	2.27.E-04
F26D11.12	10.11	1.35	2.54	3.35.E-03
F26D11.20	12.22	2.26	4.78	2.64.E-04
F27E5.9	19.19	1.19	2.27	1.65.E-03
F28B4.3	45.09	1.49	2.82	1.39.E-11
F28H7.3	134.63	1.19	2.29	1.07.E-08
F31F6.2	11.73	1.07	2.10	8.41.E-03
F33E2.4	1.43	3.54	11.63	1.99.E-04
F33H12.7	61.84	2.25	4.77	8.43.E-22
F35C8.5	70.56	1.16	2.24	2.59.E-08
F35E12.10	94.21	1.72	3.29	5.41.E-14
F35E12.2	1.59	1.48	2.78	4.02.E-03
F35E12.6	266.60	3.11	8.61	9.79.E-45
F35E8.10	1.56	3.59	12.04	1.40.E-03
F35F10.1	13.69	1.17	2.25	5.82.E-04
F35F10.13	44.50	3.20	9.18	1.04.E-18
F35F10.5	38.61	3.30	9.83	2.20.E-18
F35F10.6	4.84	1.48	2.78	1.52.E-03
F35F10.7	1.27	3.28	9.69	5.11.E-03
F36F2.2	36.78	1.03	2.05	1.45.E-04
F38B2.6	4.97	1.91	3.77	1.60.E-03
F41G4.7	1.72	1.83	3.56	4.94.E-03
F41H10.1	5.84	1.81	3.51	4.82.E-04
F42A10.7	40.83	3.26	9.57	1.40.E-09
F42A6.1	0.98	2.78	6.86	6.32.E-03
F42G2.5	6.08	1.75	3.36	9.18.E-06
F43C11.7	17.16	1.31	2.47	4.66.E-05
F46A9.1	1.27	4.69	25.77	7.11.E-04
F47B8.4	9.36	1.93	3.81	4.54.E-06

F49C12.14	18.56	1.34	2.53	1.01.E-03
F49C12.7	38.27	2.06	4.17	1.73.E-15
F49F1.7	147.65	3.01	8.05	9.21.E-39
F52E1.14	547.76	1.19	2.28	7.61.E-12
F53C11.1	50.16	1.21	2.32	9.02.E-09
F53E10.5	0.55	3.75	13.49	6.96.E-03
F54B11.11	1.47	2.35	5.10	2.72.E-04
F54B8.4	18.19	2.04	4.12	2.31.E-05
F54D5.3	474.25	1.03	2.04	4.73.E-08
F54D5.4	762.45	1.56	2.95	2.56.E-17
F54D8.10	3.95	2.85	7.23	3.05.E-03
F54E2.1	214.60	1.47	2.78	1.82.E-12
F54H5.2	1.43	2.38	5.21	8.60.E-04
F55B11.4	133.91	1.22	2.33	1.26.E-10
F55B11.6	1.59	3.76	13.57	6.03.E-03
F55F3.2	20.50	1.78	3.43	2.29.E-09
F55G1.7	11.28	1.13	2.19	3.00.E-03
F55G11.2	60.50	4.04	16.51	3.37.E-47
F55G11.8	40.37	1.49	2.80	1.09.E-10
F55G7.1	0.71	3.14	8.79	4.14.E-03
F56A4.12	2.66	2.03	4.09	1.35.E-04
F56A4.2	840.32	2.14	4.42	3.04.E-30
F56C9.7	270.41	1.12	2.18	3.33.E-10
F56D2.5	7.54	1.90	3.74	5.59.E-08
F56F10.1	70.14	1.15	2.22	1.96.E-10
F57B9.3	3.68	2.84	7.18	6.82.E-08
F58B6.1	9.17	2.03	4.08	5.75.E-07
F59B1.10	2.98	1.80	3.48	2.38.E-04
F59B1.2	296.05	1.25	2.38	1.99.E-12
F59B2.12	0.44	6.28	77.87	9.71.E-03
far-3	84.76	2.04	4.11	4.32.E-12
fat-7	10.03	1.21	2.32	5.22.E-04
fbxa-105	6.06	1.08	2.12	5.46.E-03
fbxa-120	8.75	1.04	2.06	7.17.E-03
fbxa-138	1.17	3.28	9.69	3.37.E-03
fbxa-14	4.62	1.10	2.14	8.55.E-03
fbxa-150	8.10	1.27	2.42	3.08.E-04
fbxa-151	3.82	2.41	5.32	1.82.E-04
fbxa-157	3.06	1.35	2.54	8.34.E-03
fbxa-158	1.47	1.63	3.09	6.93.E-03

fbxa-163	4.98	2.26	4.78	7.46.E-09
fbxa-164	1.32	2.67	6.36	6.74.E-04
fbxa-18	2.36	2.32	4.98	2.59.E-03
fbxa-182	9.62	1.51	2.84	5.24.E-07
fbxa-2	2.50	1.32	2.50	3.75.E-03
fbxa-3	2.94	1.45	2.72	2.88.E-03
fbxa-30	2.76	2.19	4.55	1.23.E-04
fbxa-35	1.79	3.54	11.63	1.74.E-04
fbxa-36	1.35	3.14	8.79	1.44.E-03
fbxa-37	8.22	1.68	3.20	6.47.E-05
fbxa-4	4.80	1.82	3.52	8.34.E-05
fbxa-43	1.97	2.94	7.69	1.71.E-04
fbxa-54	8.54	1.13	2.18	2.84.E-03
fbxa-55	3.63	1.43	2.69	1.98.E-03
fbxa-59	22.90	1.39	2.63	3.99.E-05
fbxa-60	74.58	1.98	3.94	1.04.E-19
fbxa-63	4.78	1.48	2.79	3.18.E-03
fbxa-69	2.33	1.64	3.11	2.58.E-03
fbxa-74	7.46	1.47	2.78	9.35.E-05
fbxa-77	5.62	1.32	2.50	2.61.E-03
fbxa-78	9.18	1.44	2.71	3.47.E-04
fbxa-79	7.83	1.47	2.77	2.46.E-04
fbxa-88	3.40	1.80	3.47	6.11.E-04
fbxa-95	36.61	1.43	2.69	5.01.E-10
fbxa-98	6.88	1.26	2.39	1.34.E-03
fbxb-7	27.62	1.33	2.52	1.44.E-05
fbxc-1	8.73	1.39	2.63	2.45.E-04
fbxc-2	7.80	1.31	2.49	3.56.E-04
fbxc-23	3.95	1.74	3.33	1.14.E-03
fbxc-3	7.63	1.24	2.36	8.49.E-04
fbxc-5	7.56	1.07	2.10	5.61.E-03
fbxc-54	6.51	1.03	2.04	7.66.E-03
fipr-1	24.44	1.24	2.36	4.65.E-03
fipr-24	4.96	1.54	2.91	7.89.E-03
folt-3	0.72	2.17	4.50	8.42.E-03
fpn-1.1	20.87	1.16	2.23	1.23.E-05
frpr-5	2.66	1.34	2.53	4.91.E-03
fut-6	10.42	1.09	2.12	6.51.E-04
gba-2	5.58	4.31	19.83	1.21.E-08
gcy-19	1.27	1.34	2.54	3.26.E-03

ges-1	36.20	1.08	2.12	5.82.E-07
glb-14	2.43	1.55	2.93	9.17.E-03
gly-14	8.33	1.01	2.02	2.32.E-03
gnrr-2	2.39	1.28	2.43	9.39.E-03
gpa-12	6.72	1.16	2.24	2.09.E-03
gpa-17	15.02	1.01	2.01	1.93.E-03
gpa-4	3.42	1.47	2.77	5.43.E-04
gpdh-1	8.21	1.37	2.59	9.38.E-05
gst-24	58.34	2.60	6.06	1.19.E-26
gst-29	5.08	1.71	3.26	5.49.E-04
gst-5	185.43	2.54	5.83	4.20.E-29
gst-6	42.13	1.59	3.01	4.22.E-07
H14E04.3	9.08	1.37	2.58	9.32.E-04
H25K10.1	6.40	2.19	4.56	1.38.E-06
H27M09.5	1.29	2.94	7.67	1.67.E-03
H34I24.2	112.00	1.09	2.13	3.48.E-08
hen-1	13.62	2.37	5.18	2.89.E-07
hizr-1	3.71	1.21	2.31	4.84.E-03
hpo-6	113.83	1.41	2.65	5.65.E-12
hrg-3	6.63	1.54	2.90	8.71.E-03
icmt-1	6.23	3.15	8.87	5.17.E-06
ins-7	43.96	1.53	2.90	6.35.E-06
irg-1	11.85	2.13	4.37	1.54.E-05
irg-3	39.85	1.64	3.11	3.53.E-09
irg-4	146.24	3.81	14.02	2.03.E-28
irg-5	96.27	4.93	30.45	2.02.E-48
irg-6	19.35	5.45	43.59	1.76.E-25
K04A8.1	27.71	1.51	2.85	9.70.E-09
K05B2.4	4.80	1.83	3.56	6.41.E-05
K05C4.4	4.66	1.29	2.44	1.22.E-03
K06G5.1	370.75	1.28	2.42	6.52.E-12
K07C5.13	2.49	2.35	5.10	4.41.E-03
K08D10.14	4.79	1.41	2.66	1.61.E-03
K08D8.5	192.69	2.25	4.76	4.68.E-28
K08D8.6	88.67	1.61	3.05	8.61.E-12
K08E4.2	46.06	1.00	2.00	5.44.E-05
K09C4.5	13.54	1.20	2.30	1.33.E-04
K10C2.8	10.67	1.52	2.87	4.61.E-04
K10D11.2	8.55	2.59	6.02	3.60.E-09
K10D11.5	25.37	1.07	2.10	2.00.E-05

K11H12.11	8.00	1.96	3.89	1.32.E-06
K12C11.6	18.15	1.13	2.18	3.33.E-03
K12H4.7	335.01	1.07	2.11	4.94.E-09
kgb-2	4.04	1.41	2.66	3.90.E-03
lact-1	11.67	2.11	4.32	5.00.E-09
lact-6	1.65	3.97	15.69	5.52.E-05
lbp-5	73.77	1.02	2.03	2.55.E-04
lbp-8	11.11	2.13	4.37	2.05.E-05
lec-11	42.48	1.43	2.69	3.88.E-09
lec-6	1435.60	1.10	2.15	3.64.E-08
linc-40	2.93	1.99	3.97	7.26.E-04
lipl-2	25.31	1.31	2.48	1.60.E-05
lmp-2	8.40	1.29	2.45	6.31.E-04
lst-5	4.58	1.21	2.31	4.31.E-03
lys-1	879.92	1.10	2.15	1.47.E-09
lys-3	22.11	1.54	2.90	3.86.E-06
lys-8	651.04	1.60	3.03	5.64.E-15
M01G12.9	11.44	1.07	2.10	4.57.E-05
M02D8.2	4.45	2.15	4.43	2.59.E-03
M02H5.8	345.67	1.56	2.94	3.68.E-17
M04C3.2	2.81	1.17	2.26	6.06.E-03
M04D5.3	24.20	1.13	2.19	3.07.E-04
M28.10	206.54	1.37	2.59	7.28.E-13
math-20	8.52	1.49	2.81	3.00.E-05
math-22	9.43	1.07	2.10	4.00.E-03
math-24	7.94	1.24	2.36	1.49.E-03
math-27	6.33	1.11	2.16	5.11.E-03
mec-3	3.02	1.24	2.37	8.55.E-03
mif-1	120.80	1.48	2.80	5.96.E-12
mul-1	28.02	3.20	9.22	2.13.E-15
nas-5	4.90	1.58	2.99	1.10.E-03
nceh-1	9.75	1.62	3.07	1.47.E-06
nep-18	2.66	1.24	2.37	6.12.E-03
nhr-112	11.16	1.62	3.07	6.16.E-06
nhr-123	1.64	2.18	4.54	1.13.E-03
nhr-176	9.15	1.52	2.86	2.55.E-05
nhr-180	3.46	1.29	2.44	3.46.E-03
nhr-247	2.00	2.88	7.36	1.47.E-03
nit-1	35.73	1.64	3.12	1.81.E-10
nlp-73	25.73	1.24	2.36	4.22.E-04

nnt-1	9.43	1.65	3.13	2.05.E-09
npa-1	127.78	1.09	2.13	4.06.E-08
nspd-7	15.59	2.20	4.58	4.48.E-05
nspe-2	6.71	1.97	3.92	5.01.E-03
numr-1	16.95	2.77	6.80	1.00.E-20
numr-2	14.45	2.68	6.43	1.46.E-18
oac-3	1.55	2.22	4.67	6.19.E-04
oac-32	15.19	2.89	7.41	8.85.E-12
oac-5	1.68	2.78	6.86	3.27.E-06
oac-53	0.79	2.87	7.31	5.71.E-04
oac-55	2.39	2.84	7.17	4.71.E-08
oac-57	2.22	1.43	2.70	3.67.E-03
oac-6	36.66	1.86	3.63	1.81.E-11
oac-7	2.24	1.81	3.52	9.27.E-05
pals-14	4.21	1.86	3.64	5.57.E-05
pals-17	8.55	1.19	2.27	1.98.E-03
pals-23	17.94	1.42	2.68	4.73.E-05
pals-24	22.78	1.43	2.69	1.95.E-06
pals-37	1.70	1.83	3.56	1.41.E-03
pals-6	10.14	2.05	4.14	2.96.E-08
parg-2	3.44	1.87	3.66	8.03.E-05
pcp-1	42.48	1.51	2.84	2.14.E-07
PDB1.1	26.25	1.04	2.05	3.50.E-05
pgp-5	4.61	1.61	3.06	5.41.E-05
pgp-6	3.59	1.09	2.12	1.31.E-03
pgp-8	6.79	4.17	18.05	2.14.E-26
plpp-1.2	10.40	1.20	2.30	7.89.E-05
pmp-4	13.18	1.03	2.05	4.69.E-05
prmt-6	12.61	1.07	2.10	2.49.E-03
R02C2.7	4.92	2.24	4.72	3.26.E-04
R03G8.3	9.34	1.25	2.37	9.73.E-05
R03H10.1	15.02	1.32	2.51	8.18.E-05
R03H10.6	3.74	2.44	5.42	3.41.E-08
R04A9.9	6.09	1.38	2.61	7.73.E-03
R05D8.9	2.87	5.37	41.31	5.57.E-05
R06A10.1	2.73	1.71	3.27	8.60.E-03
R08F11.1	17.85	1.10	2.15	9.99.E-06
R09A1.3	6.68	1.61	3.06	9.73.E-06
R09E10.2	1.68	3.53	11.52	2.02.E-03
R09F10.1	36.62	1.24	2.36	8.14.E-09

R10F2.6	2.99	1.00	2.01	2.17.E-03
R11H6.4	13.82	1.03	2.04	4.67.E-03
rgs-10	9.25	1.16	2.23	8.75.E-03
rpr-1	259.33	2.85	7.21	7.48.E-34
scav-6	10.14	1.39	2.61	1.29.E-05
scl-5	24.39	1.06	2.08	6.91.E-04
sek-4	7.25	1.24	2.36	1.41.E-03
set-28	4.09	5.65	50.33	1.82.E-05
sid-2	28.63	1.24	2.36	5.47.E-06
skr-3	121.11	1.03	2.04	1.79.E-07
skr-4	88.79	1.46	2.75	4.45.E-07
snt-2	23.93	1.02	2.03	1.03.E-05
sox-4	13.04	1.08	2.11	1.65.E-03
spe-15	8.58	1.34	2.54	3.78.E-07
spp-16	39.31	1.31	2.48	3.25.E-05
spp-18	121.20	1.20	2.29	2.29.E-07
spp-2	100.34	1.22	2.33	2.15.E-06
spp-4	76.14	1.59	3.01	7.62.E-08
spp-5	2402.53	1.23	2.34	3.13.E-11
spp-8	79.67	1.02	2.03	6.26.E-08
sqst-2	9.29	1.05	2.07	2.06.E-03
sri-36	1.95	3.34	10.15	7.90.E-05
srj-29	0.74	3.86	14.48	5.50.E-03
srp-2	32.75	1.17	2.25	6.61.E-06
srw-86	4.85	2.42	5.37	2.87.E-04
str-144	0.88	2.89	7.41	6.78.E-03
str-44	0.67	3.59	12.01	9.01.E-03
swt-6	134.33	1.58	2.99	1.39.E-17
T01D3.6	114.27	2.04	4.11	1.72.E-25
T02B5.3	7.25	1.69	3.22	9.46.E-07
T02C5.1	169.98	1.02	2.03	1.43.E-07
T07A5.7	1.68	2.00	4.01	1.69.E-03
T09F3.4	15.31	1.03	2.04	1.38.E-03
T10B11.5	3.71	1.45	2.73	3.12.E-03
T15B7.15	1.44	2.27	4.83	1.11.E-03
T16G1.4	3.75	1.69	3.24	6.80.E-03
T16G1.5	5.74	2.87	7.31	1.55.E-09
T16G1.6	33.67	3.62	12.33	3.60.E-19
T16G1.7	10.72	2.64	6.25	7.51.E-10
T19D12.2	8.54	1.14	2.20	3.28.E-05

T20D3.2	456.02	1.15	2.22	3.96.E-11
T20D4.11	24.74	1.74	3.33	5.25.E-07
T20D4.5	1.07	1.75	3.36	3.70.E-03
T20D4.7	15.28	1.25	2.38	1.06.E-03
T21C12.8	7.30	2.14	4.41	4.63.E-07
T21D12.12	24.01	1.08	2.11	1.26.E-03
T22B7.7	34.28	1.18	2.27	5.03.E-06
T24A6.7	1.65	2.89	7.42	3.88.E-03
T24B8.5	499.59	1.17	2.24	5.18.E-06
T24C4.4	43.71	1.26	2.39	2.42.E-05
T24E12.5	3.49	1.84	3.59	7.06.E-07
T25D3.3	15.76	1.43	2.69	1.77.E-06
T25G12.13	1.58	3.87	14.57	8.65.E-04
T25G12.6	4.15	1.36	2.57	2.40.E-04
T26H5.9	150.36	1.24	2.35	5.72.E-12
T28A11.16	14.02	1.21	2.32	1.92.E-03
T28A11.17	1.64	4.75	26.96	5.86.E-06
T28A11.19	28.20	2.92	7.57	1.42.E-13
T28A11.2	171.20	1.40	2.64	2.87.E-11
T28A11.20	2.58	3.41	10.64	1.61.E-06
T28A11.25	14.06	1.68	3.20	1.70.E-05
T28A11.3	5.45	3.23	9.41	1.68.E-06
T28F3.8	31.13	1.02	2.03	2.37.E-05
tag-244	31.05	3.00	7.99	6.03.E-23
tba-7	17.93	1.04	2.06	1.54.E-05
thn-1	2.14	1.75	3.37	7.51.E-03
thn-2	180.99	1.80	3.48	5.73.E-14
tre-4	6.54	1.20	2.30	1.98.E-04
tsp-1	28.13	1.46	2.75	1.86.E-06
ttm-2	16.84	1.15	2.21	1.87.E-04
ttr-32	42.77	1.07	2.10	5.36.E-05
ttr-41	312.62	1.20	2.29	9.38.E-11
ttr-44	119.04	1.23	2.35	2.19.E-10
ttr-46	422.07	1.29	2.44	1.94.E-10
ttr-48	221.54	1.53	2.89	3.44.E-16
ttr-49	18.81	1.03	2.04	2.13.E-03
twk-28	4.52	1.22	2.33	2.29.E-03
ugt-1	5.00	1.50	2.82	9.52.E-05
ugt-13	6.91	1.97	3.91	8.50.E-07
ugt-14	4.39	3.04	8.25	5.41.E-11

ugt-16	23.62	2.83	7.13	1.69.E-22
ugt-18	1.66	1.78	3.43	5.24.E-03
ugt-21	7.47	1.79	3.46	9.45.E-08
ugt-24	3.06	1.70	3.25	6.12.E-05
ugt-25	19.11	1.58	2.98	1.08.E-08
ugt-26	14.38	1.40	2.64	9.26.E-06
ugt-30	2.99	1.20	2.30	4.58.E-03
ugt-31	6.96	2.19	4.57	1.42.E-06
ugt-32	2.14	1.49	2.80	6.46.E-03
ugt-33	1.54	2.69	6.45	3.11.E-04
ugt-39	9.91	1.36	2.57	1.32.E-05
ugt-41	21.06	2.41	5.32	1.02.E-13
ugt-43	12.95	1.56	2.94	9.09.E-07
ugt-47	31.03	1.10	2.14	3.87.E-05
ugt-5	11.21	1.55	2.92	3.76.E-06
ugt-6	8.32	1.55	2.93	4.28.E-06
ugt-66	6.72	2.48	5.60	6.68.E-08
ugt-8	3.56	2.08	4.22	2.04.E-05
ugt-9	5.93	1.68	3.20	3.60.E-05
valv-1	78.46	1.23	2.35	2.49.E-05
vem-1	99.08	1.27	2.40	8.30.E-13
VZK822L.2	12.62	1.59	3.02	4.27.E-04
W01A11.1	101.79	1.03	2.04	3.11.E-08
W01C9.2	6.76	2.66	6.34	1.05.E-04
W02B12.1	25.61	1.49	2.82	1.72.E-08
W02H3.1	6.49	1.27	2.41	2.80.E-03
W02H5.11	1.00	2.46	5.50	4.64.E-03
W04C9.5	2.76	1.81	3.50	2.42.E-03
W04C9.6	2.07	2.14	4.40	6.69.E-04
W04E12.7	12.30	1.24	2.37	1.09.E-03
W04G5.8	1.02	2.81	7.03	8.99.E-04
W05E10.1	4.05	1.15	2.22	7.55.E-03
W05H9.1	316.79	1.73	3.31	5.73.E-10
W05H9.3	169.87	1.07	2.10	3.05.E-09
W06G6.17	3.44	1.95	3.87	9.80.E-03
W07G1.25	7.42	2.01	4.03	1.16.E-03
W08E12.2	4.72	2.48	5.58	6.39.E-03
xtr-1	5.55	1.80	3.47	2.68.E-04
Y110A2AL.9	19.44	1.06	2.08	2.63.E-03
Y119D3B.13	47.80	1.10	2.14	1.15.E-05

Y119D3B.21	1584.10	1.60	3.03	1.68.E-16
Y14H12A.5	8.21	2.24	4.71	2.17.E-04
Y17D7B.2	5.91	2.32	5.00	3.50.E-04
Y17D7B.3	1.14	3.16	8.91	6.88.E-03
Y17G7B.23	0.97	3.76	13.57	6.47.E-03
Y17G7B.8	30.96	1.31	2.48	9.45.E-09
Y18H1A.11	5.48	1.36	2.56	2.27.E-03
Y19D10A.11	3.15	1.58	3.00	9.86.E-04
Y22D7AL.15	103.55	1.32	2.49	1.11.E-10
Y22D7AR.2	0.98	1.49	2.80	2.65.E-03
Y24D9B.1	4.21	1.67	3.18	3.03.E-05
Y34B4A.5	31.60	1.97	3.90	5.16.E-10
Y34B4A.6	448.68	1.42	2.67	4.82.E-15
Y34F4.1	20.63	1.83	3.57	1.88.E-07
Y34F4.4	7.71	1.33	2.52	2.51.E-03
Y34F4.5	41.42	1.08	2.12	1.91.E-05
Y34F4.6	6.92	2.54	5.83	8.85.E-06
Y37H2A.10	1.98	3.91	15.04	5.67.E-03
Y37H2A.14	71.14	2.28	4.85	2.24.E-17
Y37H2A.18	8.25	1.19	2.28	5.86.E-03
Y37H2A.7	6.51	1.40	2.63	8.48.E-04
Y38C1AA.6	4.80	1.46	2.75	2.27.E-03
Y38F1A.8	8.61	1.16	2.24	1.71.E-03
Y38H6C.9	1.57	1.99	3.97	6.09.E-03
Y39B6A.25	1.81	2.83	7.10	1.60.E-05
Y39B6A.9	2.87	2.02	4.06	1.84.E-03
Y39H10B.2	28.78	1.47	2.77	5.46.E-07
Y41D4B.15	3.50	3.11	8.64	5.24.E-07
Y41D4B.17	7.47	1.37	2.58	3.83.E-04
Y43C5A.2	129.62	1.02	2.03	1.59.E-08
Y43F8B.14	2.02	1.44	2.72	2.79.E-03
Y45G5AM.3	14.13	1.78	3.44	3.36.E-08
Y46C8AM.1	2.53	2.10	4.30	2.10.E-03
Y46D2A.2	29.14	1.19	2.29	3.06.E-06
Y46G5A.20	29.90	1.79	3.46	6.79.E-07
Y47D3B.12	4.30	1.88	3.69	1.29.E-03
Y47G7B.2	9.49	1.65	3.14	2.06.E-07
Y47H10A.3	2.41	1.94	3.83	9.95.E-04
Y47H10A.5	125.51	1.31	2.48	1.32.E-09
Y48G1C.5	0.80	1.81	3.51	1.24.E-03

Y48G8AR.2	7.09	1.50	2.83	6.25.E-04
Y49E10.18	45.23	1.30	2.46	9.83.E-10
Y50C1A.2	1.90	3.09	8.54	5.49.E-05
Y51A2D.8	0.74	3.15	8.85	6.11.E-03
Y51B9A.8	3.62	3.69	12.92	1.84.E-04
Y51B9A.9	5.53	2.20	4.60	6.26.E-06
Y51H4A.13	3.41	2.01	4.04	5.81.E-05
Y52E8A.3	4.40	1.64	3.11	8.67.E-03
Y53F4B.11	4.76	1.67	3.18	1.46.E-03
Y53G8AL.1	2.14	1.56	2.96	1.29.E-04
Y54G11A.4	6.01	1.49	2.80	2.79.E-04
Y54G2A.49	35.98	1.75	3.36	4.76.E-07
Y54G2A.7	10.21	1.33	2.52	1.27.E-03
Y55F3AM.21	2.42	1.48	2.79	7.46.E-03
Y55F3BR.11	13.36	1.46	2.76	4.41.E-04
Y57G11B.5	229.48	1.02	2.03	1.23.E-04
Y57G11C.40	10.33	1.65	3.14	2.32.E-04
Y57G11C.41	3.94	1.80	3.47	2.68.E-03
Y57G7A.1	20.13	1.12	2.17	2.60.E-03
Y58A7A.1	30.43	1.05	2.07	2.05.E-03
Y66A7A.4	1.92	2.80	6.96	1.53.E-05
Y66H1A.5	33.69	1.16	2.23	3.06.E-05
Y70G10A.2	0.18	3.79	13.84	8.67.E-03
Y71F9B.13	12.07	1.07	2.10	1.07.E-03
Y71G12A.4	3.13	1.54	2.90	7.58.E-04
Y71G12B.2	3.21	2.00	4.00	6.34.E-04
Y71G12B.35	5.54	1.68	3.21	1.48.E-04
Y71G12B.5	0.44	2.09	4.25	4.73.E-03
Y73B3B.1	1.10	2.46	5.50	3.85.E-03
Y75B7B.1	1.87	3.13	8.74	5.98.E-05
Y75B8A.23	7.41	2.89	7.44	1.66.E-05
Y75B8A.33	1.03	2.84	7.16	4.35.E-03
Y76B12C.4	9.90	2.30	4.94	2.93.E-07
Y77E11A.12	3.66	1.15	2.22	3.69.E-03
Y82E9BL.18	3.56	1.84	3.57	2.49.E-04
ZC239.13	2.90	2.16	4.47	8.14.E-03
ZC239.14	7.97	2.52	5.75	7.41.E-08
ZC239.15	2.24	2.10	4.27	2.06.E-03
ZC376.2	25.98	1.62	3.06	6.84.E-10
ZC412.10	8.75	1.26	2.40	5.51.E-03

zig-9	25.30	1.19	2.28	4.06.E-06
ZK1055.6	14.41	1.13	2.19	3.23.E-05
ZK1055.7	42.98	1.44	2.71	7.76.E-11
ZK1240.2	8.85	1.09	2.13	2.92.E-03
ZK1290.10	10.34	2.28	4.86	1.27.E-09
ZK1290.13	8.82	1.09	2.13	2.55.E-03
ZK1290.14	26.45	1.36	2.56	7.72.E-05
ZK1290.5	15.07	1.08	2.12	2.78.E-04
ZK1320.2	264.18	1.16	2.23	7.31.E-09
ZK1320.3	494.43	1.66	3.15	1.25.E-18
ZK228.3	21.51	1.08	2.11	3.08.E-04
ZK287.9	22.38	1.60	3.02	2.56.E-05
ZK418.7	10.80	1.11	2.15	6.17.E-04
ZK484.6	2.21	2.22	4.65	4.07.E-03
ZK6.11	574.58	1.53	2.88	1.02.E-16
ZK896.1	2.60	2.11	4.32	5.64.E-05
ZK896.4	10.96	1.95	3.86	2.77.E-07
ZK896.5	26.46	2.41	5.33	3.19.E-17
ZK899.2	57.77	1.30	2.47	9.88.E-12

Supplementary Table8

RNA sequencing analysis of animals exposed to CPT at 2°C for 3 hr.
(logFC<-1 p<0.01)

Name	Max group mean	Log_2 fold change	Fold change	P-value
aagr-4	15.06	-1.22	-2.34	1.58.E-05
abu-12	4.05	-1.65	-3.14	1.13.E-03
abu-13	11.60	-1.56	-2.95	1.93.E-03
abu-14	34.80	-1.47	-2.77	4.34.E-03
acdh-1	263.41	-1.76	-3.39	8.40.E-04
acdh-2	3.46	-2.53	-5.78	4.27.E-05
ace-3	2.20	-1.33	-2.52	2.31.E-03
acn-1	14.54	-1.64	-3.12	2.67.E-05
acs-2	19.32	-2.47	-5.56	5.05.E-09
acsd-1	6.42	-1.15	-2.22	2.03.E-03
agmo-1	6.35	-1.75	-3.37	2.95.E-03
agr-1	1.95	-1.28	-2.43	1.33.E-04
anc-1	11.21	-1.75	-3.37	2.78.E-15
anp-1	9.30	-2.42	-5.36	3.03.E-16
aptf-4	9.02	-1.68	-3.20	2.23.E-06
aqp-2	135.21	-1.33	-2.52	3.80.E-08
arrd-1	26.73	-2.09	-4.27	6.90.E-07
arrd-4	7.25	-1.38	-2.59	4.52.E-04
ast-1	3.69	-1.79	-3.45	9.27.E-05
atf-2	5.39	-1.77	-3.41	5.58.E-06
atf-8	3.93	-1.96	-3.90	7.07.E-04
atln-2	2.25	-1.25	-2.37	7.98.E-03
atz-1	50.94	-1.23	-2.34	2.41.E-05
B0228.6	10.29	-1.63	-3.10	2.63.E-05
B0281.5	28.05	-1.10	-2.15	8.43.E-04
B0310.2	6.55	-1.05	-2.06	2.43.E-03
B0393.5	2.61	-1.78	-3.44	6.69.E-06
bah-1	4.18	-2.83	-7.10	4.92.E-05
bath-10	5.85	-1.74	-3.33	3.80.E-04
bath-9	9.94	-1.24	-2.37	1.11.E-03
BE0003N10.3	2.41	-1.08	-2.11	9.20.E-03
best-7	4.70	-1.14	-2.21	4.39.E-03
bli-1	3.14	-2.42	-5.35	2.28.E-04
bli-2	4.80	-2.32	-5.00	3.48.E-03
bro-1	1.02	-2.59	-6.01	8.79.E-03

btb-11	8.56	-1.42	-2.67	6.85.E-04
btb-8	8.06	-1.74	-3.34	2.85.E-05
bus-12	2.85	-2.34	-5.05	4.09.E-05
bus-18	1.28	-2.62	-6.16	3.94.E-04
bus-19	4.89	-1.29	-2.45	1.26.E-03
bus-4	2.71	-1.54	-2.91	3.38.E-03
bus-8	11.29	-1.94	-3.84	5.34.E-04
C01A2.1	1.70	-1.98	-3.96	5.10.E-03
C01G10.6	3.92	-2.70	-6.48	5.10.E-03
C01G6.9	1.63	-1.79	-3.46	7.53.E-03
C02E7.6	285.48	-1.65	-3.14	4.80.E-03
C02F12.5	52.04	-1.40	-2.63	1.13.E-06
C03A3.1	2.67	-1.79	-3.46	1.85.E-04
C03C10.5	3.99	-3.93	-15.27	2.61.E-04
C03D6.9	20.47	-1.91	-3.76	2.13.E-04
C03H5.6	1.83	-2.03	-4.08	4.12.E-03
C05D10.4	7.47	-1.06	-2.09	8.29.E-04
C05D9.7	18.36	-1.15	-2.21	5.67.E-04
C05E7.1	5.41	-1.35	-2.55	9.10.E-03
C05G5.7	4.77	-1.85	-3.59	3.46.E-03
C06E7.4	12.72	-1.31	-2.48	2.18.E-05
C06E7.88	2.40	-3.54	-11.66	1.15.E-03
C06G1.1	6.61	-1.54	-2.91	2.80.E-03
C08F1.10	22.31	-1.75	-3.37	1.84.E-06
C08F1.11	3.85	-1.93	-3.80	1.48.E-04
C08F1.6	18.02	-1.46	-2.76	3.32.E-04
C08F11.12	228.10	-1.02	-2.02	1.04.E-08
C09F9.2	12.94	-2.74	-6.68	2.97.E-16
C10F3.7	2.68	-4.07	-16.85	1.55.E-04
C13C12.2	3.12	-1.57	-2.96	4.62.E-03
C14A4.9	8.35	-2.50	-5.65	1.32.E-05
C14B4.2	4.01	-1.00	-2.00	4.40.E-04
C14F11.6	25.30	-2.02	-4.06	2.85.E-06
C15C6.1	9.31	-1.24	-2.36	3.48.E-03
C17E4.20	33.50	-1.40	-2.64	6.46.E-08
C18D11.6	14.98	-2.02	-4.04	1.37.E-04
C18H7.1	0.95	-2.58	-5.98	8.80.E-04
C23H3.9	2.71	-1.67	-3.19	2.17.E-05

C25F6.7	5.35	-2.10	-4.28	3.00.E-06
C26B9.3	15.47	-1.59	-3.00	1.18.E-03
C26F1.1	15.44	-1.28	-2.43	4.43.E-03
C27A7.8	16.16	-1.75	-3.37	6.99.E-06
C28C12.4	12.95	-1.34	-2.53	8.01.E-05
C28G1.4	2.06	-1.02	-2.03	7.07.E-03
C29A12.6	0.52	-3.88	-14.70	2.59.E-04
C32H11.11	3.48	-7.03	-130.48	6.41.E-03
C32H11.5	3.19	-2.90	-7.47	1.85.E-04
C33D9.6	6.16	-1.05	-2.07	5.14.E-04
C33E10.1	3.14	-2.03	-4.08	2.63.E-03
C34E7.4	47.73	-1.15	-2.22	3.36.E-06
C34F6.1	2.18	-1.65	-3.13	2.08.E-03
C35A5.10	2.10	-2.07	-4.21	2.59.E-03
C35A5.11	6.52	-2.73	-6.65	1.02.E-06
C35E7.5	30.52	-2.21	-4.63	2.65.E-17
C39B5.2	9.89	-1.02	-2.03	7.07.E-03
C40A11.4	4.96	-1.55	-2.92	2.97.E-03
C40A11.6	3.82	-3.36	-10.28	2.14.E-06
C44H9.7	1.49	-2.65	-6.29	1.27.E-03
C50D2.1	3.44	-1.95	-3.87	4.12.E-05
C50F7.5	88.82	-1.99	-3.97	9.01.E-08
C54C8.12	6.68	-4.95	-31.01	2.01.E-04
C54G4.4	0.76	-2.45	-5.46	2.30.E-04
C55B7.3	3.36	-1.61	-3.06	7.61.E-04
cav-1	354.90	-1.72	-3.29	4.42.E-09
cbn-1	3.39	-2.06	-4.17	8.65.E-06
cdh-10	2.83	-1.87	-3.65	5.85.E-04
cdh-12	4.50	-2.04	-4.11	3.80.E-07
cdh-4	8.91	-1.07	-2.10	3.15.E-08
cdh-5	1.58	-1.14	-2.21	9.62.E-04
cec-8	18.43	-1.49	-2.81	8.13.E-07
ced-11	3.93	-1.25	-2.37	5.64.E-04
ced-8	5.27	-1.18	-2.27	5.63.E-03
ceh-10	1.62	-1.76	-3.40	6.48.E-03
ceh-13	15.15	-1.59	-3.01	8.10.E-07
ceh-22	1.66	-1.99	-3.97	9.42.E-04
ceh-27	7.98	-2.21	-4.64	9.95.E-07

ceh-30	1.90	-1.68	-3.21	4.86.E-03
ceh-32	11.83	-1.92	-3.79	2.45.E-09
ceh-34	5.60	-1.27	-2.41	2.43.E-03
ceh-36	4.81	-2.05	-4.14	2.36.E-03
ceh-43	14.14	-1.85	-3.60	1.98.E-07
ceh-48	2.84	-1.27	-2.42	3.88.E-03
ceh-86	5.36	-1.37	-2.58	1.96.E-04
ces-2	11.37	-2.53	-5.79	1.16.E-09
cfz-2	9.87	-2.13	-4.37	7.57.E-10
chd-7	20.71	-1.04	-2.06	9.26.E-08
chs-2	1.79	-1.12	-2.17	2.19.E-03
cht-1	76.71	-1.53	-2.89	3.37.E-08
cla-1	0.55	-1.54	-2.91	5.18.E-04
clec-12	3.33	-1.64	-3.13	4.66.E-04
clec-180	7.48	-1.68	-3.20	2.35.E-04
clec-196	36.33	-1.95	-3.85	7.21.E-05
clec-223	4.68	-1.87	-3.66	4.76.E-08
clec-266	114.60	-2.34	-5.06	4.51.E-16
clec-78	2.83	-2.61	-6.09	1.45.E-18
cls-3	11.12	-1.11	-2.16	8.39.E-07
cmk-1	5.53	-1.08	-2.11	3.65.E-03
cnc-2	48.11	-2.48	-5.60	2.03.E-09
cnc-4	50.54	-1.88	-3.69	1.08.E-07
cog-1	2.15	-1.55	-2.92	6.10.E-03
col-107	139.20	-1.56	-2.94	3.54.E-03
col-117	318.90	-1.45	-2.74	2.66.E-03
col-121	7.24	-3.00	-7.99	6.98.E-12
col-130	17.28	-1.57	-2.98	2.18.E-03
col-131	2.91	-2.32	-4.99	7.48.E-04
col-138	17.75	-1.69	-3.24	6.54.E-03
col-147	61.78	-1.60	-3.03	4.37.E-03
col-149	37.46	-1.99	-3.96	8.12.E-04
col-162	19.05	-1.43	-2.69	9.86.E-03
col-165	26.93	-7.16	-142.96	6.24.E-23
col-168	72.79	-1.70	-3.25	5.74.E-03
col-172	1.71	-1.78	-3.44	4.94.E-03
col-175	9.23	-2.21	-4.63	5.64.E-03
col-43	6.85	-1.39	-2.62	1.60.E-03

col-46	3.46	-2.00	-4.00	1.12.E-03
col-48	25.94	-1.19	-2.28	4.72.E-03
col-63	7.40	-1.96	-3.90	1.72.E-03
col-65	32.74	-1.91	-3.76	1.09.E-03
col-71	20.55	-1.57	-2.96	4.78.E-03
col-74	21.08	-5.90	-59.86	3.27.E-25
col-77	43.08	-1.71	-3.27	3.53.E-04
col-99	7.04	-2.03	-4.09	7.52.E-09
cpt-4	5.67	-1.13	-2.18	4.77.E-04
cut-2	140.92	-1.70	-3.25	5.71.E-03
cut-3	15.37	-7.50	-181.34	1.61.E-14
cut-6	2.02	-1.21	-2.31	8.60.E-03
cutl-16	4.17	-1.71	-3.27	4.02.E-05
cutl-18	1.37	-2.85	-7.20	3.58.E-06
cutl-2	5.10	-2.92	-7.57	1.19.E-07
cutl-20	4.32	-1.90	-3.72	2.96.E-05
cutl-28	2.50	-2.63	-6.17	8.90.E-08
cutl-29	1.95	-1.82	-3.53	2.30.E-03
cutl-9	2.10	-1.68	-3.20	1.03.E-03
cwn-2	7.62	-1.77	-3.41	5.37.E-07
cyd-1	15.12	-1.39	-2.62	2.92.E-05
cyn-17	3.80	-2.65	-6.26	4.89.E-08
cyn-6	71.19	-1.05	-2.07	5.58.E-05
cyp-42A1	5.87	-1.22	-2.32	7.08.E-04
D1014.5	3.92	-2.65	-6.26	2.85.E-07
D2005.6	1.86	-1.19	-2.28	9.89.E-03
D2096.6	8.70	-1.50	-2.83	2.41.E-03
daam-1	1.78	-1.60	-3.03	1.11.E-04
daao-1	5.03	-1.21	-2.32	6.44.E-03
dao-2	178.18	-1.36	-2.56	1.50.E-06
dct-5	1.42	-2.43	-5.38	8.73.E-03
dct-7	9.62	-4.16	-17.92	3.05.E-05
dct-8	15.09	-4.13	-17.52	1.62.E-07
ddo-2	6.04	-1.05	-2.07	6.45.E-03
ddr-2	3.96	-1.09	-2.12	2.83.E-03
deg-1	1.80	-1.26	-2.39	5.58.E-03
del-2	0.81	-2.23	-4.70	1.79.E-03
dex-1	6.12	-1.72	-3.30	6.02.E-09

dgk-1	3.11	-1.07	-2.10	1.72.E-03
dhs-25	41.78	-1.07	-2.10	2.98.E-05
dig-1	1.61	-1.91	-3.76	7.26.E-12
dma-1	2.51	-1.56	-2.96	8.17.E-04
dmd-5	2.28	-3.57	-11.90	1.11.E-04
dos-1	3.87	-1.39	-2.62	5.70.E-03
dpy-10	35.13	-2.45	-5.47	1.32.E-12
dpy-14	114.38	-3.94	-15.37	9.51.E-48
dpy-17	127.09	-3.41	-10.60	1.90.E-44
dpy-2	29.07	-2.56	-5.88	1.44.E-10
dpy-20	1.85	-2.53	-5.78	1.98.E-03
dpy-3	40.78	-2.65	-6.28	5.03.E-10
dpy-7	29.95	-2.67	-6.34	1.96.E-11
dpy-8	24.14	-1.93	-3.81	1.81.E-04
dpy-9	16.40	-1.31	-2.47	4.70.E-03
drd-10	36.74	-4.27	-19.33	1.50.E-09
dre-1	18.94	-1.21	-2.31	3.41.E-09
dsl-2	27.38	-1.52	-2.87	4.06.E-06
dsl-3	13.18	-1.22	-2.32	3.11.E-03
dsl-6	3.28	-3.29	-9.81	2.26.E-06
dyc-1	2.91	-1.32	-2.50	5.27.E-04
dyf-7	2.30	-1.21	-2.31	9.07.E-03
E01G4.6	6.49	-2.18	-4.54	2.59.E-04
EEED8.15	18.61	-1.27	-2.41	1.95.E-05
EEED8.2	12.55	-1.11	-2.16	2.09.E-03
efl-3	15.51	-1.44	-2.71	1.63.E-05
egas-3	0.80	-1.84	-3.58	4.85.E-03
egl-23	1.77	-1.28	-2.43	5.01.E-03
egl-38	2.20	-1.87	-3.64	4.37.E-03
egl-46	11.63	-2.74	-6.68	7.33.E-11
epg-2	66.94	-2.13	-4.36	2.97.E-20
epi-1	15.62	-1.66	-3.17	2.34.E-16
ets-7	3.54	-1.79	-3.45	1.02.E-03
eva-1	7.01	-1.22	-2.34	7.05.E-04
exc-9	19.76	-1.39	-2.62	7.18.E-05
eya-1	8.86	-1.60	-3.02	4.25.E-05
F01G10.10	3.58	-1.30	-2.47	3.76.E-03
F07H5.13	23.38	-1.32	-2.50	1.32.E-04

F09F7.6	42.02	-1.34	-2.52	7.75.E-05
F09F9.2	10.50	-1.76	-3.38	1.90.E-03
F10D7.3	8.64	-1.36	-2.57	8.67.E-04
F13B12.4	1.55	-1.98	-3.95	1.30.E-03
F13C5.2	47.74	-1.12	-2.18	1.79.E-07
F13E9.11	11.70	-2.14	-4.40	1.38.E-10
F14B4.1	4.70	-1.56	-2.95	8.05.E-05
F14B6.3	12.39	-1.06	-2.08	2.96.E-03
F15B9.8	24.61	-1.44	-2.70	1.02.E-03
F15E6.3	78.00	-2.74	-6.70	1.13.E-09
F15E6.4	74.92	-2.22	-4.67	7.33.E-07
F18C5.5	3.63	-2.52	-5.72	1.34.E-04
F20B10.3	3.49	-1.99	-3.97	8.88.E-04
F20C5.6	13.76	-1.02	-2.03	5.34.E-05
F23F12.8	0.60	-2.59	-6.02	3.87.E-04
F23H12.5	4.52	-1.94	-3.85	6.54.E-05
F26F12.4	3.51	-1.50	-2.83	3.09.E-03
F28C6.5	3.73	-1.96	-3.90	1.77.E-03
F30A10.2	3.88	-2.04	-4.11	8.02.E-04
F30H5.3	8.63	-2.54	-5.82	2.29.E-09
F32D8.12	19.21	-1.15	-2.22	1.26.E-05
F33D4.6	13.22	-1.85	-3.61	1.23.E-04
F33E2.5	22.52	-1.67	-3.19	2.17.E-06
F34D10.6	1.09	-1.31	-2.48	3.14.E-03
F34D6.1	3.92	-1.69	-3.22	4.19.E-04
F35D2.3	13.07	-1.71	-3.27	1.33.E-07
F36H1.11	3.06	-2.48	-5.60	3.96.E-03
F36H5.13	3.07	-2.58	-5.98	1.86.E-06
F36H5.4	3.87	-1.77	-3.40	5.63.E-04
F37A4.3	1.49	-1.64	-3.12	9.82.E-03
F40E10.5	5.62	-1.32	-2.49	3.37.E-03
F40G9.15	1.68	-3.06	-8.37	1.62.E-03
F41E7.1	5.91	-1.71	-3.27	5.99.E-07
F41F3.8	4.59	-1.65	-3.13	3.16.E-03
F43D9.1	4.88	-1.61	-3.05	1.78.E-08
F44E2.4	7.38	-1.09	-2.13	1.38.E-03
F44F1.6	5.06	-1.64	-3.11	2.88.E-04
F45C12.17	3.29	-1.46	-2.75	8.09.E-03

F45D11.5	5.19	-1.84	-3.58	3.69.E-03
F45D3.4	181.51	-1.34	-2.53	5.52.E-06
F46C8.8	8.50	-1.39	-2.62	5.81.E-03
F46E10.2	44.53	-1.58	-2.99	4.04.E-05
F46G11.6	2.11	-1.88	-3.69	9.91.E-03
F47B8.5	1.66	-3.50	-11.29	4.40.E-04
F47E1.4	2.45	-1.33	-2.51	1.08.E-03
F48E3.6	8.23	-1.93	-3.82	1.29.E-05
F49D11.6	0.91	-3.06	-8.34	8.03.E-03
F49E10.2	1.93	-1.82	-3.52	5.72.E-03
F49E12.12	1.45	-1.82	-3.53	9.30.E-03
F53A10.2	12.42	-1.05	-2.07	8.76.E-06
F53B1.4	21.34	-1.93	-3.81	6.08.E-06
F53B3.5	34.66	-1.65	-3.14	5.41.E-12
F53C3.11	1.28	-2.39	-5.24	5.92.E-04
F53G2.1	1.92	-2.47	-5.53	2.32.E-05
F56D2.3	5.55	-1.97	-3.90	6.80.E-05
F56D3.1	21.02	-2.18	-4.53	3.82.E-07
F56D6.8	15.10	-4.35	-20.37	4.09.E-08
F56D6.9	11.51	-3.21	-9.25	1.08.E-04
F58D2.3	1.48	-3.20	-9.19	5.32.E-03
F58E6.13	3.48	-1.49	-2.80	4.40.E-04
F58H1.2	8.85	-1.93	-3.81	1.16.E-04
F59D6.1	2.16	-2.04	-4.11	8.31.E-03
F59F5.7	1.37	-2.41	-5.30	1.20.E-05
fasn-1	35.68	-1.80	-3.47	1.40.E-22
fbn-1	7.32	-2.59	-6.03	2.15.E-14
fbxa-103	0.83	-2.86	-7.27	4.10.E-03
fbxa-24	2.76	-1.82	-3.53	2.32.E-03
fbxb-10	3.47	-1.46	-2.75	2.36.E-03
fbxb-102	1.87	-1.68	-3.21	8.83.E-03
fbxb-108	1.66	-2.70	-6.48	1.54.E-04
fbxb-17	4.99	-1.41	-2.65	1.80.E-03
fbxb-18	2.52	-2.02	-4.06	1.02.E-03
fbxb-19	1.82	-3.40	-10.57	4.07.E-04
fbxb-24	4.71	-1.53	-2.88	1.94.E-03
fbxb-30	1.87	-2.90	-7.48	2.55.E-04
fbxb-35	3.65	-1.73	-3.31	6.57.E-04

fbxb-37	3.85	-1.19	-2.28	8.75.E-03
fbxb-40	3.97	-1.61	-3.06	6.03.E-03
fbxb-41	7.01	-1.36	-2.56	1.42.E-03
fbxb-42	4.16	-1.24	-2.37	4.68.E-03
fbxb-43	3.60	-1.58	-2.99	1.39.E-03
fbxb-44	4.81	-1.24	-2.36	8.10.E-03
fbxb-45	4.28	-1.95	-3.87	2.25.E-04
fbxb-51	2.72	-2.11	-4.33	1.32.E-03
fbxb-57	2.23	-1.51	-2.84	8.83.E-03
fbxb-65	1.67	-3.58	-11.95	2.91.E-04
fbxb-66	6.93	-2.41	-5.33	3.86.E-07
fbxb-74	3.09	-1.84	-3.58	9.75.E-04
fbxb-75	5.02	-2.06	-4.18	1.34.E-04
fbxb-77	1.21	-2.16	-4.47	2.18.E-03
fbxb-78	1.71	-1.98	-3.94	9.81.E-03
fbxb-88	5.18	-1.90	-3.73	2.21.E-05
fbxb-90	2.74	-1.99	-3.98	1.02.E-03
fbxb-91	4.88	-1.68	-3.19	3.56.E-04
fbxc-14	2.52	-2.22	-4.67	3.14.E-04
fbxc-18	5.52	-1.90	-3.74	1.36.E-05
fbxc-19	1.58	-2.06	-4.17	5.18.E-04
fbxc-21	2.76	-1.46	-2.75	4.05.E-03
fbxc-24	3.57	-1.37	-2.59	3.24.E-03
fbxc-28	8.54	-1.11	-2.16	7.45.E-03
fbxc-34	1.99	-2.32	-5.00	1.55.E-03
fbxc-51	22.22	-1.99	-3.98	4.48.E-10
fip-5	23.06	-1.35	-2.55	3.51.E-04
fipr-22	5.84	-2.24	-4.72	1.27.E-05
fkb-5	33.91	-1.63	-3.10	3.67.E-08
fkb-7	6.11	-2.18	-4.52	3.00.E-08
fkh-2	3.33	-2.25	-4.75	1.25.E-03
flh-2	24.02	-1.73	-3.31	5.74.E-11
fmi-1	3.34	-1.76	-3.39	1.68.E-10
fmil-1	1.90	-1.34	-2.52	5.84.E-04
fmo-2	3.19	-1.22	-2.34	8.68.E-03
gadr-5	2.49	-1.08	-2.12	4.06.E-03
gap-1	2.62	-1.26	-2.40	4.00.E-03
gcy-18	0.62	-1.72	-3.29	4.51.E-03

gei-13	9.56	-2.19	-4.56	1.59.E-10
glf-1	15.42	-2.55	-5.86	3.89.E-08
gly-12	3.61	-1.04	-2.05	8.00.E-03
gon-1	4.78	-1.11	-2.16	7.91.E-05
grdn-1	8.23	-1.48	-2.79	5.21.E-09
grh-1	4.40	-1.19	-2.28	3.72.E-03
grl-15	13.39	-1.62	-3.06	5.19.E-03
grl-16	56.90	-1.58	-2.98	7.96.E-03
grl-5	13.08	-1.92	-3.78	6.47.E-04
grl-7	29.40	-2.08	-4.23	2.72.E-04
H03E18.1	6.20	-1.57	-2.98	1.68.E-04
H37A05.2	1.78	-1.81	-3.50	3.14.E-03
H42K12.3	18.98	-2.38	-5.21	6.42.E-07
ham-1	15.04	-1.36	-2.57	2.52.E-05
hbl-1	12.32	-2.29	-4.90	7.87.E-29
hch-1	11.55	-3.57	-11.87	1.25.E-20
hhat-1	5.00	-2.17	-4.51	5.57.E-05
hil-2	235.26	-2.05	-4.14	2.84.E-11
hil-3	70.22	-1.50	-2.83	2.23.E-10
hil-6	1.62	-2.71	-6.53	9.20.E-03
hil-7	32.88	-2.82	-7.05	9.00.E-21
his-24	389.00	-2.30	-4.92	5.23.E-13
his-46	4.57	-2.51	-5.71	2.39.E-03
his-55	5.95	-2.04	-4.11	1.64.E-03
his-58	7.94	-1.83	-3.55	3.03.E-04
his-60	19.49	-1.21	-2.31	2.75.E-04
his-63	6.15	-1.55	-2.92	1.85.E-03
hlh-1	7.18	-1.53	-2.88	8.26.E-05
hlh-16	2.61	-2.68	-6.40	4.71.E-03
hmg-11	139.40	-1.40	-2.63	1.59.E-07
hphd-1	88.08	-1.11	-2.16	9.80.E-08
hst-3.2	3.38	-1.72	-3.29	1.48.E-03
icl-1	115.02	-1.54	-2.91	1.29.E-17
ifa-3	3.17	-3.73	-13.25	4.91.E-11
ifa-4	36.30	-1.43	-2.69	6.05.E-16
ifc-1	14.33	-1.35	-2.55	2.07.E-03
ifp-1	7.86	-1.29	-2.44	3.96.E-03
igcm-1	5.02	-1.87	-3.67	1.26.E-06

igcm-3	35.46	-1.63	-3.09	4.07.E-13
igdb-1	3.47	-6.13	-70.17	2.52.E-09
igdb-3	0.62	-2.54	-5.81	4.05.E-03
ikb-1	3.88	-1.03	-2.04	6.91.E-03
ins-19	6.10	-5.15	-35.58	1.72.E-04
ins-2	2.56	-2.83	-7.11	1.51.E-03
ins-34	13.59	-1.12	-2.17	9.86.E-03
irx-1	8.23	-1.00	-2.01	1.16.E-03
jmjc-1	8.70	-1.19	-2.29	7.05.E-05
jmjd-3.1	7.41	-1.52	-2.86	4.45.E-09
jmjd-3.2	4.81	-1.80	-3.47	4.15.E-05
jph-1	12.89	-1.01	-2.02	7.11.E-04
K01A2.4	2.77	-1.52	-2.88	2.71.E-04
K01D12.1	1.19	-2.63	-6.19	6.83.E-03
K02B12.2	12.96	-1.16	-2.24	7.09.E-03
K02E10.4	5.85	-1.79	-3.47	2.33.E-05
K02E11.10	11.78	-1.48	-2.79	9.54.E-03
K04H4.2	9.54	-2.68	-6.42	3.78.E-09
K05C4.9	3.53	-1.20	-2.29	1.64.E-03
K07C11.7	58.50	-1.90	-3.72	3.78.E-17
K08B12.1	8.00	-2.58	-5.96	1.91.E-07
K08B4.2	7.22	-1.83	-3.54	5.43.E-04
K08E7.5	3.33	-1.81	-3.50	4.25.E-04
K10D3.4	15.35	-1.89	-3.71	1.26.E-08
K10G6.4	1.02	-2.07	-4.19	3.79.E-03
K11G9.5	5.16	-1.48	-2.79	3.52.E-04
kal-1	9.62	-1.18	-2.27	6.86.E-05
kcc-2	12.75	-1.01	-2.01	1.01.E-05
kin-33	2.99	-1.33	-2.52	6.14.E-03
klp-11	3.77	-1.10	-2.14	1.45.E-03
lad-2	1.32	-1.07	-2.10	6.38.E-03
lam-1	36.71	-1.23	-2.35	1.47.E-09
lam-2	22.86	-1.07	-2.10	2.92.E-08
lam-3	5.43	-1.54	-2.91	1.40.E-09
ldb-1	26.02	-1.09	-2.13	4.25.E-09
lect-2	13.06	-1.13	-2.18	3.00.E-04
let-4	6.54	-2.00	-4.01	9.83.E-08
let-653	6.62	-1.38	-2.61	2.48.E-05

let-805	11.69	-1.31	-2.49	1.29.E-06
lgc-27	8.29	-1.57	-2.96	5.48.E-04
lgx-1	2.02	-1.75	-3.37	3.18.E-06
lin-12	4.42	-1.23	-2.34	1.05.E-04
lin-17	8.08	-1.17	-2.25	1.98.E-04
lin-32	2.75	-3.09	-8.52	3.41.E-03
lin-39	6.72	-1.11	-2.15	4.42.E-03
lin-42	15.70	-1.63	-3.10	1.44.E-04
lips-3	5.99	-1.29	-2.45	5.94.E-03
lips-9	3.17	-1.90	-3.72	2.55.E-03
lon-3	19.02	-1.58	-2.98	8.28.E-03
lpr-3	32.31	-2.24	-4.72	1.23.E-08
lpr-4	20.58	-2.31	-4.97	7.14.E-07
lpr-5	17.49	-2.38	-5.22	6.19.E-08
lpr-6	19.15	-2.31	-4.95	4.19.E-08
lron-2	3.56	-1.38	-2.60	4.86.E-03
lrp-1	7.26	-1.37	-2.59	2.82.E-09
M01D1.12	1.03	-2.51	-5.69	6.98.E-03
M02G9.1	1.31	-1.57	-2.98	6.85.E-04
M03B6.3	4.90	-2.45	-5.45	5.40.E-07
M03D4.4	5.42	-2.33	-5.03	1.08.E-08
M116.1	9.71	-1.42	-2.68	3.01.E-04
madd-2	7.23	-1.45	-2.73	2.13.E-05
madd-4	3.92	-1.17	-2.26	4.04.E-03
magu-3	3.14	-1.17	-2.25	8.08.E-03
mam-1	0.88	-1.56	-2.95	5.82.E-03
mca-1	36.16	-1.05	-2.07	1.40.E-09
mec-7	14.50	-1.52	-2.87	2.00.E-05
mec-8	23.29	-1.17	-2.25	3.99.E-06
mig-6	46.11	-1.11	-2.16	3.91.E-09
mls-2	3.14	-2.11	-4.33	1.83.E-05
mlt-10	20.99	-1.70	-3.26	4.27.E-07
mlt-11	14.43	-2.78	-6.87	5.80.E-11
mlt-7	9.66	-1.36	-2.58	1.02.E-06
mlt-8	41.62	-1.48	-2.78	9.24.E-05
mlt-9	10.32	-1.64	-3.11	9.25.E-04
mltn-9	5.44	-1.64	-3.11	4.99.E-07
mnp-1	14.63	-1.80	-3.49	7.21.E-10

moe-3	12.82	-1.17	-2.26	4.61.E-04
mps-2	9.28	-1.59	-3.01	1.53.E-06
mpz-1	2.99	-1.02	-2.02	2.58.E-04
myo-3	24.82	-1.10	-2.15	1.33.E-04
myo-6	3.06	-1.05	-2.07	6.95.E-03
nab-1	12.27	-1.52	-2.87	5.50.E-09
nas-37	8.71	-1.15	-2.22	2.89.E-03
ncam-1	9.35	-1.31	-2.48	4.79.E-07
ncx-9	1.47	-2.13	-4.39	6.41.E-04
ngn-1	2.90	-3.66	-12.65	3.50.E-04
nhr-114	12.41	-1.32	-2.49	5.35.E-05
nhr-25	7.60	-1.56	-2.96	1.08.E-06
nhr-85	4.03	-1.05	-2.07	5.12.E-03
nlp-24	98.05	-1.18	-2.26	1.71.E-04
nlp-28	64.04	-1.81	-3.51	5.13.E-07
nlp-29	56.40	-1.75	-3.36	2.72.E-07
nlp-31	39.76	-1.36	-2.57	5.13.E-04
nlp-34	44.57	-1.36	-2.57	7.61.E-03
nlp-39	15.05	-3.13	-8.78	1.03.E-06
nlr-1	2.75	-1.03	-2.04	4.23.E-03
noah-1	40.59	-2.26	-4.79	7.44.E-16
noah-2	48.97	-2.46	-5.52	2.28.E-17
nob-1	3.38	-1.48	-2.80	1.88.E-04
nrx-1	1.61	-1.18	-2.26	1.71.E-03
nstp-3	1.91	-1.87	-3.65	4.16.E-03
nsy-4	12.51	-1.04	-2.06	1.25.E-03
oac-1	1.07	-2.31	-4.96	3.62.E-03
oac-51	2.19	-1.93	-3.82	1.53.E-04
odr-2	1.96	-1.70	-3.24	9.30.E-03
ora-1	17.59	-1.50	-2.83	3.30.E-03
osr-1	1.74	-1.90	-3.73	1.08.E-03
pat-12	17.76	-1.03	-2.04	8.05.E-08
pat-9	7.45	-1.53	-2.89	1.95.E-07
pax-3	3.00	-3.20	-9.21	5.44.E-06
pbo-5	3.81	-1.57	-2.97	7.33.E-04
peb-1	4.74	-1.11	-2.16	2.94.E-03
pes-2.2	29.96	-1.11	-2.16	3.33.E-06
pha-4	14.57	-1.18	-2.26	4.04.E-07

phat-1	1.91	-2.94	-7.66	1.52.E-04
phat-4	11.38	-1.63	-3.09	3.57.E-03
phat-5	5.84	-3.27	-9.62	1.15.E-08
pitr-3	4.96	-1.20	-2.29	3.56.E-04
plpr-1	6.24	-1.60	-3.02	7.10.E-04
plx-2	2.82	-1.43	-2.69	4.57.E-06
poml-4	0.84	-3.19	-9.14	4.76.E-03
pqn-13	5.09	-1.57	-2.98	7.25.E-05
pqn-32	15.39	-1.91	-3.75	2.53.E-05
pqn-36	3.12	-1.33	-2.51	1.68.E-04
pqn-71	2.78	-1.94	-3.82	7.72.E-04
pqn-73	3.34	-1.42	-2.67	1.51.E-03
prkl-1	6.39	-1.16	-2.23	2.97.E-04
pros-1	3.38	-1.67	-3.19	3.74.E-05
ptp-3	13.96	-1.02	-2.03	3.15.E-09
ptr-10	1.36	-2.22	-4.65	7.81.E-05
ptr-16	3.28	-1.96	-3.89	1.85.E-04
ptr-18	7.20	-2.15	-4.44	3.66.E-06
ptr-4	10.94	-1.84	-3.59	7.25.E-05
qua-1	15.35	-1.51	-2.85	1.20.E-03
R02D5.1	15.29	-1.16	-2.24	6.86.E-04
R02F11.1	23.67	-2.02	-4.05	3.64.E-05
R03G8.6	6.80	-1.11	-2.16	2.54.E-04
R03H10.11	6.41	-1.44	-2.71	7.73.E-03
R03H10.2	9.25	-2.03	-4.08	6.64.E-08
R04A9.7	4.10	-1.38	-2.60	5.15.E-03
R05A10.1	1.90	-4.24	-18.85	1.71.E-04
R05G9R.1	0.68	-1.83	-3.56	4.99.E-03
R07E3.6	5.68	-1.22	-2.33	2.79.E-03
R09E10.5	3.32	-3.31	-9.90	2.64.E-15
R10E12.2	9.11	-1.02	-2.02	1.65.E-03
R166.6	10.91	-7.53	-184.53	5.69.E-10
R173.3	0.97	-2.03	-4.07	9.47.E-04
R193.2	6.09	-1.13	-2.19	2.48.E-04
ref-2	3.67	-2.16	-4.46	1.21.E-05
rga-2	4.06	-1.37	-2.58	4.45.E-05
rga-9	16.02	-1.07	-2.10	2.11.E-06
rgs-1	4.40	-1.52	-2.87	6.16.E-04

rhgf-2	5.70	-1.34	-2.53	4.75.E-08
rocf-1	0.79	-2.56	-5.89	1.03.E-05
rol-1	8.47	-2.35	-5.10	6.62.E-04
rol-6	46.57	-1.86	-3.63	8.64.E-03
rol-8	78.72	-2.02	-4.05	7.95.E-04
sad-1	3.36	-1.20	-2.30	7.14.E-04
sams-5	2.87	-1.49	-2.81	2.80.E-03
sdz-24	18.39	-1.63	-3.09	5.85.E-04
sdz-25	9.57	-1.62	-3.07	4.57.E-05
sdz-28	16.75	-1.80	-3.48	3.58.E-06
sdz-30	13.32	-2.16	-4.46	2.03.E-06
sdz-33	5.02	-1.77	-3.42	1.45.E-04
sdz-4	7.98	-1.75	-3.37	1.28.E-04
sdz-5	3.56	-1.36	-2.56	4.77.E-03
sdz-9	5.32	-1.85	-3.61	4.79.E-05
sem-2	9.62	-1.11	-2.15	4.07.E-04
sepa-1	20.08	-1.79	-3.46	1.57.E-13
shn-1	10.36	-1.38	-2.60	2.02.E-09
shw-3	0.92	-1.64	-3.12	8.82.E-03
skpo-2	5.50	-1.53	-2.89	6.05.E-03
skr-10	44.37	-1.46	-2.74	1.60.E-06
skr-13	26.35	-1.40	-2.64	6.43.E-05
skr-21	23.30	-1.20	-2.30	1.52.E-04
skr-7	100.06	-1.02	-2.03	1.67.E-05
skr-8	58.31	-1.63	-3.10	7.36.E-09
skr-9	29.03	-1.79	-3.47	5.87.E-07
slo-1	3.43	-1.21	-2.32	7.03.E-04
slt-1	2.45	-1.23	-2.34	9.15.E-04
snf-11	4.78	-1.12	-2.17	4.51.E-03
somi-1	17.81	-1.16	-2.24	5.12.E-06
sox-2	11.23	-1.09	-2.12	2.01.E-05
sptf-1	4.98	-2.27	-4.84	8.71.E-06
sqt-1	55.96	-2.02	-4.06	3.08.E-03
sqt-2	78.65	-2.17	-4.49	3.44.E-04
sqt-3	297.18	-2.54	-5.83	3.23.E-13
srap-1	6.39	-1.54	-2.90	3.57.E-07
ssp-9	2.93	-4.23	-18.80	6.13.E-03
sto-1	39.27	-1.08	-2.12	5.40.E-07

subs-4	3.31	-1.86	-3.63	9.28.E-04
sup-26	91.60	-1.34	-2.54	1.19.E-08
sup-37	17.27	-1.26	-2.39	1.87.E-11
sups-1	6.00	-1.50	-2.83	3.36.E-04
suro-1	11.33	-1.36	-2.56	2.39.E-03
swt-5	1.82	-1.88	-3.69	6.11.E-03
syd-9	8.12	-2.17	-4.51	5.35.E-14
sym-1	39.19	-2.24	-4.71	4.46.E-12
sym-2	9.91	-1.14	-2.20	1.47.E-04
T01D3.3	1.54	-1.65	-3.14	7.02.E-05
T02E9.5	44.34	-2.61	-6.09	9.73.E-15
T03F1.6	13.71	-1.30	-2.46	8.07.E-04
T03G6.1	11.90	-2.20	-4.58	6.74.E-07
T04C12.1	2.44	-3.80	-13.91	4.69.E-03
T05D4.2	15.09	-1.34	-2.54	1.15.E-04
T05H10.3	14.03	-3.63	-12.35	1.08.E-14
T06D8.10	2.74	-1.40	-2.63	7.55.E-03
T08G5.3	5.06	-1.48	-2.79	7.85.E-03
T09B4.5	76.36	-1.65	-3.15	1.61.E-12
T14B4.19	21.42	-2.05	-4.14	2.75.E-06
T14E8.4	0.67	-2.79	-6.89	5.82.E-03
T16G1.2	2.17	-1.57	-2.96	7.52.E-03
T19A5.3	7.79	-1.98	-3.94	1.16.E-05
T19C3.2	5.92	-1.51	-2.84	3.29.E-03
T19C3.3	27.34	-1.92	-3.80	4.39.E-06
T19C4.1	7.01	-1.32	-2.50	1.90.E-03
T20F5.4	4.74	-2.01	-4.04	2.87.E-07
T23C6.4	21.08	-1.13	-2.18	2.18.E-05
T23F2.13	7.91	-1.86	-3.62	5.01.E-04
T24B8.3	197.69	-1.42	-2.68	2.39.E-13
T24E12.13	1.31	-2.31	-4.96	8.67.E-03
T24F1.7	4.47	-2.51	-5.70	1.90.E-03
T25E12.6	16.49	-1.23	-2.35	1.86.E-03
T26C12.3	5.64	-1.49	-2.80	2.03.E-03
T26C5.2	4.93	-1.95	-3.87	3.78.E-04
T26E3.8	4.62	-1.31	-2.48	8.68.E-03
T27A1.3	2.05	-2.45	-5.46	4.18.E-04
T28B8.1	39.31	-1.24	-2.36	1.71.E-08

T28F4.1	18.06	-1.00	-2.00	2.73.E-05
tag-275	5.43	-1.05	-2.08	2.96.E-03
tba-8	3.55	-2.55	-5.86	1.91.E-04
tbb-4	4.14	-2.13	-4.38	2.97.E-06
tbx-8	3.88	-2.00	-4.00	1.65.E-04
tep-1	31.44	-1.13	-2.18	9.30.E-09
timm-17B.2	32.40	-1.24	-2.36	9.11.E-04
tsp-14	7.23	-1.29	-2.45	4.50.E-04
ttn-1	1.24	-1.58	-2.98	5.54.E-07
ttr-50	159.20	-1.32	-2.49	4.41.E-05
tts-1	383.50	-1.69	-3.23	1.74.E-13
tts-2	44.58	-1.68	-3.21	2.70.E-05
ttx-1	3.76	-1.27	-2.41	4.14.E-03
twk-11	0.64	-2.15	-4.45	3.86.E-05
ugt-57	3.40	-2.60	-6.08	2.62.E-06
unc-120	16.37	-1.21	-2.32	4.90.E-05
unc-13	3.94	-1.19	-2.28	4.07.E-04
unc-130	5.61	-1.91	-3.75	1.08.E-05
unc-2	2.51	-1.13	-2.18	1.51.E-04
unc-22	6.89	-1.22	-2.34	3.91.E-05
unc-3	1.87	-1.72	-3.29	4.92.E-04
unc-30	2.49	-1.95	-3.85	3.75.E-04
unc-39	3.49	-3.39	-10.47	6.13.E-06
unc-41	2.07	-1.12	-2.18	2.28.E-03
unc-42	2.45	-1.93	-3.80	1.48.E-03
unc-53	7.45	-1.12	-2.18	1.06.E-05
unc-6	6.86	-1.40	-2.65	1.68.E-05
unc-7	2.90	-1.23	-2.34	1.43.E-03
unc-75	2.74	-1.96	-3.90	4.31.E-06
unc-77	1.16	-1.36	-2.57	1.21.E-03
unc-8	0.58	-1.88	-3.69	8.80.E-03
unc-82	5.63	-1.03	-2.04	4.15.E-05
unc-86	3.35	-1.60	-3.04	5.93.E-04
vab-2	13.98	-1.12	-2.17	3.54.E-04
vab-23	10.23	-1.48	-2.79	2.96.E-04
vab-7	1.95	-1.78	-3.42	8.87.E-03
vet-2	19.32	-1.19	-2.27	4.48.E-06
vet-6	15.18	-1.63	-3.10	1.23.E-06

W02F12.8	5.12	-2.11	-4.32	7.29.E-03
W03F9.4	2.67	-1.69	-3.22	1.87.E-04
W03G11.4	8.86	-1.06	-2.08	4.63.E-04
W04A8.4	34.41	-1.41	-2.65	1.24.E-06
W06H8.5	7.96	-2.53	-5.76	4.75.E-04
W08G11.1	1.48	-2.38	-5.20	1.70.E-03
W10G11.1	1.52	-3.93	-15.27	3.86.E-03
W10G11.2	5.27	-1.36	-2.57	7.76.E-03
wrt-1	14.46	-2.46	-5.49	3.15.E-08
wrt-10	45.17	-2.31	-4.97	1.20.E-09
wrt-2	11.76	-2.66	-6.33	4.78.E-09
wrt-4	5.07	-2.06	-4.16	3.86.E-05
wrt-6	2.43	-1.52	-2.87	8.27.E-03
wrt-9	1.93	-1.38	-2.61	8.05.E-03
Y102A11A.5	17.77	-1.41	-2.66	4.80.E-03
Y105C5B.3	0.71	-3.07	-8.39	6.23.E-03
Y105E8B.9	3.32	-1.21	-2.32	1.95.E-03
Y106G6D.2	9.43	-1.28	-2.43	8.86.E-04
Y110A2AL.4	10.70	-1.48	-2.80	4.59.E-05
Y116F11A.1	1.65	-2.56	-5.90	5.59.E-03
Y11D7A.5	4.31	-1.71	-3.26	5.31.E-03
Y17D7C.6	2.88	-3.78	-13.78	1.17.E-03
Y18H1A.9	3.59	-1.33	-2.52	5.40.E-03
Y22D7AR.10	251.16	-1.15	-2.22	5.89.E-06
Y37A1B.7	10.03	-2.14	-4.40	3.93.E-05
Y38A10A.2	1.53	-1.76	-3.39	3.43.E-03
Y38H6A.5	2.20	-2.12	-4.35	8.20.E-03
Y38H6C.16	7.90	-1.50	-2.84	5.36.E-04
Y39G10AL.1	13.57	-1.16	-2.24	5.65.E-04
Y41D4B.14	8.04	-1.38	-2.60	1.91.E-03
Y41D4B.26	8.97	-2.16	-4.45	2.75.E-05
Y41D4B.6	2.23	-3.30	-9.85	3.83.E-06
Y43B11AL.1	4.05	-2.08	-4.24	3.95.E-05
Y43F8B.3	2.62	-1.94	-3.83	5.06.E-05
Y45F10C.4	93.84	-1.11	-2.16	3.15.E-06
Y45G5AM.5	15.20	-1.67	-3.19	2.23.E-04
Y46G5A.7	9.06	-1.24	-2.37	6.23.E-03
Y47D3B.6	12.86	-1.32	-2.50	4.23.E-03

Y48E1B.8	7.99	-3.18	-9.06	1.49.E-06
Y53F4B.27	2.77	-1.35	-2.54	8.75.E-03
Y54G2A.76	7.57	-2.09	-4.26	7.43.E-05
Y55B1BR.6	2.90	-1.56	-2.94	9.09.E-03
Y55D5A.1	5.23	-1.09	-2.13	6.05.E-03
Y57G11C.42	3.51	-1.32	-2.50	6.32.E-03
Y64G10A.7	3.00	-2.07	-4.19	9.01.E-09
Y65A5A.1	13.66	-1.45	-2.73	1.64.E-03
Y65B4BL.1	12.09	-1.65	-3.14	1.68.E-03
Y65B4BM.3	29.77	-1.90	-3.74	3.04.E-09
Y71A12B.11	25.73	-1.71	-3.28	1.18.E-04
Y71F9AL.7	8.43	-1.40	-2.65	1.17.E-03
Y71G12B.25	14.83	-1.02	-2.02	5.59.E-04
Y71H2AM.15	4.32	-1.30	-2.47	4.51.E-03
Y73E7A.8	4.29	-1.80	-3.49	9.86.E-05
Y75B7AR.1	14.42	-1.68	-3.20	8.16.E-03
Y75B8A.6	10.26	-2.17	-4.50	2.31.E-08
Y7A9D.1	18.00	-1.24	-2.37	1.40.E-03
Y82E9BR.1	4.06	-1.53	-2.88	1.95.E-04
Y82E9BR.17	19.92	-2.51	-5.71	4.45.E-10
Y95B8A.6	5.43	-1.10	-2.14	7.28.E-04
Y97E10AR.1	3.61	-1.39	-2.63	1.14.E-04
Y97E10C.1	36.25	-1.33	-2.51	2.92.E-05
Y9C2UA.1	3.59	-1.24	-2.36	3.21.E-03
Y9C9A.13	1.02	-1.83	-3.55	3.98.E-03
zag-1	5.16	-1.97	-3.90	2.38.E-08
ZC116.3	1.22	-1.05	-2.08	1.01.E-03
ZC123.1	6.40	-2.38	-5.19	6.13.E-07
ZC247.1	3.02	-1.49	-2.82	3.74.E-07
ZC376.1	0.79	-1.68	-3.20	9.45.E-03
ZC84.1	0.30	-2.73	-6.64	8.22.E-04
ZC84.6	0.42	-2.41	-5.31	8.83.E-04
zeel-1	2.01	-1.63	-3.09	1.38.E-04
zig-10	2.35	-1.57	-2.98	3.13.E-03
zip-7	11.78	-2.42	-5.34	6.81.E-07
zip-8	33.51	-1.62	-3.07	6.58.E-07
ZK1025.2	8.67	-2.61	-6.09	5.01.E-05
ZK1025.3	25.11	-2.61	-6.09	6.85.E-05

ZK1025.8	8.01	-2.59	-6.04	2.50.E-04
ZK1053.4	23.49	-1.14	-2.21	1.02.E-04
ZK180.6	18.82	-2.10	-4.29	3.37.E-04
ZK287.1	6.14	-2.02	-4.07	6.63.E-09
ZK430.5	2.04	-1.26	-2.40	1.65.E-03
ZK6.6	2.58	-1.26	-2.40	6.98.E-03
ZK930.6	2.71	-1.78	-3.43	3.38.E-03
ZK970.7	34.41	-1.35	-2.54	7.34.E-04
zmp-2	3.85	-1.93	-3.81	9.37.E-05
zoo-1	12.23	-1.09	-2.13	8.52.E-06
ztf-11	12.76	-1.13	-2.19	9.12.E-05
ztf-16	6.46	-1.32	-2.49	8.09.E-05
ztf-22	28.59	-1.02	-2.03	3.83.E-06
ztf-30	1.17	-1.79	-3.45	6.13.E-03
ztf-6	37.33	-1.12	-2.18	6.36.E-08

Supplementary Table9

Genes whose expression is altered by LMB exposure that overlap with genes reported as XPO1 cargo in Kirli et al, *eLife* 2015 and Kumar et al, *Sci. Adv.*, 2022.

LMB_increase_2°C (3 h)		LMB_decrease_2°C (3 h)		LMB_increase_25°C (20 h)		LMB_decrease_25°C (20 h)	
Wormbase ID	Gene name	Wormbase ID	Gene name	Wormbase ID	Gene name	Wormbase ID	Gene name
WBGene00003999	*pgp-5*	WBGene00000164	*apm-3*	WBGene00003903	*pab-2*	WBGene00002244	*laf-1*
WBGene00004000	*pgp-6*	WBGene00000479	*cgh-1*	WBGene00008034	*C39E9.11*	WBGene00004471	*rps-2*
WBGene00004001	*pgp-7*	WBGene00004007	*pgp-13*	WBGene00018321	*sand-1*	WBGene00007836	*C31C9.2*
WBGene00004002	*pgp-8*	WBGene00004132	*ifet-1*			WBGene00013674	*Y105E8A.14*
WBGene00004004	*pgp-10*	WBGene00012484	*car-1*				
WBGene00004008	*pgp-14*	WBGene00012911	*acl-7*				
WBGene00006533	*tba-7*						

Supplementally Table10

WormCat Category 2	RGS	AC	PValue
Stress response: pathogen	67	192	8.52E-50
Stress response: detoxification	35	206	2.32E-18
Stress response: C-type Lectin	35	256	8.97E-16
Proteolysis proteasome: E3	47	590	1.92E-12
Metabolism: lipid	38	526	2.51E-09
Metabolism: short chain dehydrogenase	9	42	1.48E-06
Stress response: heavy metal	5	16	7.40E-05
Extracellular material: matrix	7	67	0.001163363
Proteolysis general: aspartate	5	33	0.001341009
Proteolysis general: serine	5	34	0.001510757
Development: apoptosis	6	64	0.004227894
Stress response: unassigned	5	49	0.006342435
Metabolism: unassigned	10	192	0.013777749
Signaling: heteromeric G protein	9	170	0.01719488
Transmembrane transport: major facilitator	6	89	0.017766493
Proteolysis general: unassigned	5	68	0.021515326
Protein modification: methyltransferase	5	76	0.031895621
Metabolism: carbohydrate	5	83	0.043163515
Proteolysis general: cysteine	3	41	0.070217825
Transmembrane transport: solute carrier	8	197	0.080689022
Stress response: signaling	1	4	0.103851085
Transmembrane transport: ABC	3	50	0.107968113
Extracellular material: galectin	2	26	0.122945409
Peroxisome: transporter	1	5	0.123287659
Transmembrane protein: unassigned	45	1692	0.137818101
Transmembrane transport: potassium channel	5	121	0.140343741
Transmembrane transport: ammonium	1	6	0.14230208
Proteolysis general: metallopeptidase	5	123	0.146999574
Lysosome: acid phosphatase	2	31	0.160271613
Neuronal function: transcription factor	1	7	0.160903531
Signaling: MAPK	2	32	0.167967084
Proteolysis general: inhibitor	3	66	0.189081482
Extracellular material: chitinase	2	37	0.207235941
Transmembrane transport: acid sensing	1	10	0.214318903
Transmembrane transport: sugar	1	10	0.214318903
Proteolysis general: lysozyme	1	11	0.231356355
Transcription factor: GATA	1	11	0.231356355
Transcription factor: ETS	1	12	0.248023831
Cytoskeleton: claudin	1	15	0.295885988
Proteolysis general: carboxypeptidase	1	16	0.311152284
Transmembrane transport: unassigned	2	51	0.319669452
Protein modification: acetyltransferase	1	17	0.326087117
Signaling: lipid	3	102	0.398731004
DNA: replication	2	69	0.457287164
Signaling: unassigned	2	70	0.464474933
Metabolism: 1CC	1	29	0.481923886

Transcription factor: NHR	6	259	0.513989298
Transmembrane transport: sodium channel	1	32	0.514881582
Globin	1	36	0.555582775
Metabolism: insulin	1	39	0.583848594
Cytoskeleton: motor protein	1	41	0.601685131
Neuronal function: unassigned	1	42	0.610314233
Signaling: phosphatase	4	196	0.631599757
Transcription factor: ZF	1	53	0.693731328
Extracellular material: secreted protein	1	54	0.700363862
Metabolism: nucleotide	2	111	0.705371131
Ribosome: EIF	1	60	0.737245497
Muscle function	1	62	0.74850006
Cell cycle: chromosome dynamics	1	65	0.764483302
Signaling: S/T kinase	1	66	0.769581732
Extracellular material: collagen	3	184	0.77241365
Cytoskeleton: microtubule	2	128	0.775037264
Protein modification: carbohydrate	2	129	0.778647081
DNA: repair	1	68	0.779449415
Neuronal function: synaptic function	4	244	0.786316886
Signaling: Y kinase	1	73	0.802306812
Cytoskeleton: unassigned	1	77	0.818872716
Development: general	1	81	0.834048637
Trafficking: ER/Golgi	1	87	0.85445905
Development: somatic	1	122	0.932278776
Nucleic acid: binding	2	229	0.960915361
Non-coding RNA: linc	1	174	0.978235223
Signaling: small GTPase	1	183	0.98211397
Metabolism: mitochondria	1	366	0.999665429
Transmembrane protein: seven transmembrane receptor	6	1473	0.999999995
Non-coding RNA: ncRNA	5	7850	1
Pseudogene	1	2081	1
Unassigned	230	6343	5.60E-10
